# Evidence-based medical procedures to optimise caesarean outcomes: an overview of systematic reviews

**DOI:** 10.1016/j.eclinm.2025.103212

**Published:** 2025-04-30

**Authors:** Virginia Diaz, Celina Gialdini, Mónica Chamillard, Julia Pasquale, Guillermo Carroli, Maria Regina Torloni, Ana Pilar Betran

**Affiliations:** a Centro Rosarino de Estudios Perinatales (CREP), Rosario, Argentina; b Facultat de Ciències de la Salut Blanquerna, Universitat Ramon Llull, Barcelona, Spain; c Obstetrics Post-Graduate Program, Department of Obstetrics, São Paulo Federal University, São Paulo, Brazil; d UNDP/UNFPA/UNICEF/WHO/World Bank Special Programme of Research, Development and Research Training in Human Reproduction, Department of Sexual and Reproductive Health and Research, World Health Organization, Geneva, Switzerland

**Keywords:** Caesarean section, Medical, Public health, Systematic review, Maternal health

## Abstract

**Background:**

The use of caesarean sections (CS) is increasing to unprecedented levels worldwide. As with any surgery, it has risks, and understanding the evidence base for interventions involved in a CS is essential to optimise outcomes and inform recommendations. We conducted an overview of systematic reviews (SRs) of randomised controlled trials (RCTs) to summarise the evidence on medical procedures used in CS.

**Methods:**

Searches were conducted in Cochrane Database of Systematic Reviews, PubMed, EMBASE, LILACSs and CINAHL without date or language restrictions from database inception to January 31, 2024, with an updated search performed on January 24, 2025. We included SRs of RCTs that examined the effectiveness and safety of medical procedures used in CS. AMSTAR 2 and GRADE were used to assess the methodological quality of the SRs and the certainty of evidence at outcome level, respectively. We classified each procedure-outcome pair into one of eight categories according to effect estimates and certainty of evidence. The overview was registered at PROSPERO (CRD 42023208306).

**Findings:**

We identified 29 SRs (15 Cochrane and 14 non-Cochrane) published between 2002 and 2024 involving 408 unique RCTs including over 116,000 participants. Most reviews included trials from low- and middle-income countries (n = 21, 72.4%), combined both elective and emergency CS (n = 19, 65.5%), and were of high quality (n = 18, 62%), while 24.3% (n = 7) were of low and 13.7% (n = 4) were of critically low quality. The SRs presented 512 procedure-outcome comparisons (271 procedure versus procedure, 241 procedure versus no treatment/placebo). There was insufficient or inconclusive evidence for 350 comparisons (68.4%), clear evidence of benefit for 97 (18.9%), possible benefit for 48 (9.3%), clear or possible no difference of effect for 9 (1.8%), clear evidence of harm for 4 (0.8%) and possible harm for 4 (0.8%). We found no SRs for 13 pre-specified procedures. Indwelling bladder catheter and its immediate removal, vaginal preparation with antiseptic solution, antibiotic prophylaxis, early oral intake, and abdominal binders are associated with benefits for some outcomes. There are no SRs on post-CS wound care, stitch removal, or time to resume sexual or physical activity, among others.

**Interpretation:**

There are numerous gaps in the available evidence on medical procedures used in CS that require additional research. There is an urgent need for international recommendations to guide healthcare providers and policymakers in ensuring safer, evidence-based, care for women undergoing CS.

**Funding:**

UNDP-UNFPA-UNICEF-WHO-World Bank Special Programme of Research, Development and Research Training in Human Reproduction (HRP), a cosponsored programme executed by the 10.13039/100004423World Health Organization (WHO).

**Research in context**

**Evidence before this study**

Caesarean sections (CS) are among the most common major surgeries worldwide. In addition to the surgical technique, numerous medical procedures have been incorporated over time and continue to be tested to improve maternal and perinatal outcomes. Many systematic reviews have analysed various medical procedures involved in a CS, however, in a search conducted in Pubmed, Cochcrane Database, EMBASE, LILACS and CINAHL (from inception to 31st January 2025) using the terms “caesarean section” AND “medical procedures” AND “overview”, without language restrictions, we only identify an overview of SRs on surgical interventions published by our group in 2024. We could not find any other overview of SRs on this topic. This gap makes it challenging to identify and implement evidence-based interventions before, during, and after the operation to optimise outcomes. We therefore performed an overview of systematic reviews with meta-analysis (SRMAs) of randomised controlled trials (RCTs) of medical procedures for women during CS.

**Added value of this study**

To our knowledge, this is the first overview of systematic reviews of randomised controlled trials on medical procedures used before, during and after a caesarean section. We report results by specific “procedure-outcome” pairs (e.g., early post-operative oral intake as a procedure and abdominal distention as an outcome). We classified each procedure-outcome pair in one of eight categories according to the effectiveness of the procedure for the specific outcome and the quality of the evidence. This approach provides a useful and more granular understanding of the value of each procedure to inform decision-making in clinical practice.

**Implications of all the available evidence**

This overview identified several medical procedures used in caesareans that have clear evidence of benefit and should be recommended to optimise outcomes. It also identified numerous widely used procedures that lack conclusive evidence from systematic reviews for several important outcomes or have no systematic reviews. Our results reveal the need for rigorous and rational research in this area, and the development of international recommendations for standardised, evidence-based, caesarean section practices.

## Introduction

Over the past three decades, the average worldwide proportion of women giving birth by caesarean section (CS) has more than tripled, increasing from approximately 6% in 1990 to 21% in 2018.^1^^,^^2^ Caesareans are the most frequent major surgery globally, and projections show that by 2030, almost 30% of women worldwide will give birth by CS.^1^^,^^3^ Although a caesarean is a valuable intervention in cases of pregnancy and childbirth complications, data suggests that CS are often performed without medical reasons, a tendency attributed to many factors, including healthcare providers’ lowered thresholds for indicating the surgery, fear of litigation, convenience, and women’s preferences.^4^

As with any surgery, a CS involves multiple procedures created to optimise maternal and perinatal outcomes. The “medical procedures” involved in a CS refer to non-surgical interventions used in women undergoing a caesarean, such as pre-operative assessment, fasting, post-operative analgesia and mobilization, wound care, and drugs to prevent post-partum infection and haemorrhage.^5^, ^6^, ^7^, ^8^ Currently, there is no internationally accepted standardization for the medical procedures used in CS, and their number continues to increase in parallel with scientific advances and innovation. However, new medical procedures for women having a caesarean are often proposed without clear evidence of their effectiveness due to either insufficient and rigorous evaluation in randomised controlled trials, or the lack of meta-analyses of the results of individual trials.

The unprecedented increase in the use of CS has raised questions and concerns about its short- and long-term effects on maternal and child health, as well as financial, and societal consequences.^9^ According to current projections, 38 million annual caesareans are expected in 2030, of which 33.5 million will occur in low- and middle-income countries (LMIC).^1^ In these countries, maternal and infant health risks associated with CS are higher because access to comprehensive obstetric care is limited.^10^ Although the unnecessary use of clinical procedures can negatively impact health systems in all types of economies, the effects can be more pronounced in weaker economies with less robust systems. Therefore, there is a pressing need to identify and adopt evidence-based practices for CS that reduce risks and optimise patient outcomes, especially for low-resource settings. By prioritizing the implementation of such practices, healthcare systems can effectively reduce preventable morbidity and mortality associated with CS while alleviating strain on limited and overburdened healthcare resources. Therefore, it is essential to assess the evidence-base behind medical procedures used in women who give birth by CS.

There are no previous overviews that compiled the evidence from systematic reviews (SRs) on all procedures involved in a CS (surgical, medical, anaesthetic). This gap led us to perform a series of overviews to summarise the most up-to-date evidence on these procedures. We also aimed to identify evidence gaps to guide future research. The overview of SRs on surgical procedures has been previously published,^11^ and an overview of SRs on anaesthetic and analgesic procedures will be presented in an upcoming publication. This manuscript summarises the findings of SRs of randomised controlled trials (RCT) on medical procedures in a CS. The findings of this overview of SRs will be useful to inform evidence-based clinical practice and guidelines on CS.

## Methods

We conducted this overview of SRs according to the recommendations proposed by the Cochrane Handbook for Systematic Reviews of Interventions^12^ and present it according to the PRIOR reporting guideline).^13^ The protocol of this overview was registered at Prospero (CRD 42023208306, https://www.crd.york.ac.uk/prospero/display_record.php?RecordID=208306).

### Types of studies

We included all published SRs of RCTs that examined the effectiveness and/or safety of patient-focused medical procedures related to CS in humans. We excluded SRs of studies with other designs (e.g., cohorts, case-controls or before-and-after), and SR protocols.

### Type of participants

We included SRs of trials involving women of any age, race, socioeconomic condition, or parity, with a singleton or multiple pregnancy at any gestational age, with any foetal presentation, undergoing primary or repeat, elective or emergency CS in the first or second stage of a spontaneous or induced labour, under any type of anaesthesia. SRs that only included trials that assessed procedures in patients with specific health conditions (e.g., diabetes, obesity, HIV) were excluded.

### Type of interventions and comparators

We included SRs that assessed at least one of a list of pre-specified CS-related medical procedures conducted before, during, or after CS by health care providers or their recommendations for patients after the surgery. This list was developed based on the Coronis Trial,^14^ international guidelines,^15^, ^16^, ^17^, ^18^ a review and overview of systematic reviews,^7^^,^^8^^,^^19^ and the clinical and research experience of the overview authors, and informal consultation with international health professionals working in the field. The list included medical procedures related to: pre-operative preparation (cardiovascular evaluation, laboratory tests, pre-operative washing/bathing, shaving, fasting, intravenous (IV) fluids), bladder emptying, infection prevention (vaginal preparation, prophylactic antibiotics), post-operative recovery (time to oral intake, time to mobilization, wound care in the hospital, use of abdominal binders), discharge and post-discharge care (time of discharge, wound care at home, time for stich removal, post-discharge visits, time to resume physical activity and time to resume sexual activity), and other aspects (antenatal corticosteroids for term CS, skin to skin contact, companionship during CS, thromboprophylaxis, education/information for women, and use of protocols for medical procedures). The comparators were the alternative interventions reported in the original SRs, i.e., no treatment, usual care, or another treatment/intervention (Supplementary material 1).

Procedures to prevent postpartum haemorrhage (PPH) at CS and relevant outcomes will be presented in a separate manuscript.

### Type of outcomes

Although there are core outcome sets for some aspects of a caesarean, such as enhanced recovery and infectious morbidity,^20^, ^21^, ^22^ there is no published core outcome set for CS as a whole. Therefore, we created a list of pre-specified maternal and perinatal outcomes based on the same sources described for the list of intervention.

Pre-specified maternal outcomes were: febrile morbidity (fever, wound infection, endometritis, thrombophlebitis, peritonitis, urinary tract infection, need for antibiotics other than prophylaxis, sepsis), haemorrhagic morbidity (postpartum haemorrhage, anaemia, blood transfusion, need for additional uterotonic other than prophylaxis), pain (wound pain, pelvic pain, dysuria, headache, need for additional analgesics), short and medium-term recovery (length of hospital stay, prolonged hospital stay, ambulation, breastfeeding, self-care and ability to care for the baby without help, bonding, wound dehiscence, maternal depression), long-term complications (chronic pain, incisional hernia, intra-abdominal adhesions, sub-fertility, dyspareunia, future pregnancy complications), satisfaction with care (women and providers), acceptability, severe morbidity (hysterectomy, visceral damage, intensive care unit admission, deep vein thrombosis, pulmonary embolism, shock, cardiac arrest, pulmonary oedema, central venous access, respiratory failure, cardiopulmonary reanimation, seizures, encephalopathy, non-anaesthetic intubation, additional surgical procedures or return to operating room —e.g., re-laparotomy, arterial ligation, B-Lynch, curettage—), maternal near-miss, maternal death, and other outcomes (nausea, vomiting, operating time, readmission to hospital after discharge). The pre-specified neonatal outcomes were: respiratory distress syndrome, transient tachypnoea, low Apgar scores, infections/HIV, severe morbidity, neonatal intensive care unit admission, neonatal trauma, long-term outcomes, stillbirth, neonatal death, and perinatal death.

### Search method for identification of reviews

We created a comprehensive search strategy (Supplementary material 2) using appropriate key words (and synonyms) for CS and the list of interventions. We ran the search in five electronic databases (Cochrane Database of Systematic Reviews, PubMed, EMBASE, LILACS and CINAHL) from database inception to 31 January 2024, with an updated search performed on January 24, 2025, without language restrictions. The citations were uploaded in Covidence (https://www.covidence.org/) and duplicates deleted. We complemented the search by screening the reference lists of WHO guidelines.

### Process of review selection and data extraction

The titles and abstracts of all retrieved citations were screened to select potentially relevant studies for full-text reading; the SRs that fulfilled the aforementioned selection criteria were included in the overview. Reasons for exclusion of the studies selected for full-text reading were recorded. Screening and full-text evaluation were conducted independently by two reviewers working in pairs (MC, VD, CG, JP, APB). Conflicts were resolved through discussion with a third overview author.

For each included SR, we extracted data pertaining to all pre-specified procedures and outcomes as reported in the original SR. The data was extracted into a data collection form specifically created for this overview. One overview author extracted data and a second author checked it for accuracy. Disagreements were resolved through discussion.

### Methodological quality of systematic reviews

Two independent reviewers used the AMSTAR 2 tool^23^ to assess the overall quality of each included SR. The following critical domains were assessed, when reported, using the online tool (https://amstar.ca/Amstar_Checklist.php): (1) Protocol registered before commencement of the review; (2) Adequacy of the literature search; (3) Justification for excluding individual studies; (4) Risk of bias of individual studies included in the review; (5) Appropriateness of meta-analytical methods; (6) Consideration of risk of bias when interpreting the results of the review; (7) Assessment of presence and likely impact of publication bias.

### Certainty of the evidence for outcomes reported in the SRs

For each procedure-outcome pair, we used the GRADE assessment provided in the SRs.^24^ If it was not provided, two overview authors conducted the GRADE assessment independently. Disagreements were resolved through discussion until consensus was reached; if needed, a third overview author was called to arbitrate.

### Selection in case of duplicate comparisons and outcomes

The selection of reviews was conducted at outcome level. If more than one SR reported evidence for the same procedure-outcome pair, we applied the following selection rules:

1. We prioritised direct over indirect evidence. For example, if one SR reported outcomes specifically for women undergoing a CS and another SR reported outcomes for patients undergoing any abdominal surgery (including CS), we selected the SR that provided evidence for CS.
2. When evidence was available from a Cochrane review (CR) and a non-Cochrane review (NCR) and the search dates of the reviews were <24 months apart, we selected the CR.
3. When evidence was available from a CR and a NCR and the NCR was more recent than the CR (search date difference ≥24 months), we selected the NCR.
4. When evidence was available from two or more NCRs:
  4.1 We selected the most recent review (search date ≥24 months apart).
  4.2 If search dates were <24 months apart, we selected the NCR with the highest GRADE assessment for the outcomes of interest.
  4.3 If no GRADE assessment was reported in one of the reviews, we selected the NCR that provided GRADE assessments for the outcomes of interest.
  4.4 If the outcome had the same GRADE assessment in both reviews, we selected the SR with the highest AMSTAR 2 score.
5. When evidence was available from two or more CRs, we applied the same rules described for NCR.
6. For reviews with different search dates (≥24 months apart), but including the same studies, we selected the review with the highest AMSTAR 2 score.
7. For SRs with network meta-analysis, only direct comparisons were included and the same selections rules were applied.

### Data synthesis

We defined our unit of analysis as the “procedure-outcome” pair. For example, vaginal preparation with antiseptic solution (procedure) and surgical wound infection (outcome), or early oral intake (procedure) and abdominal distention (outcome).

We structured data synthesis as in other overviews.^25^, ^26^, ^27^, ^28^ We classified each procedure-outcome pair into one of eight mutually exclusive categories according to the pooled effect estimate and the certainty of the evidence. Box 1 presents the definitions of each category and corresponding standardised statements used in the text based on the recommendations of the Cochrane Effective Practice and Organisation of Care (EPOC)^29^ and GRADE working group.^30^ Categorization was conducted independently by two reviewers. Disagreements were resolved through discussion; when consensus was not reached, a third reviewer was called to arbitrate.

Box 1 **Categories for classification of each procedure-outcome pair in this overview.1**

**Table:** 

CI: Confidence interval; OR: Odds ratio; RR: relative risk; WMD: weighted mean difference.

^1^This box was adapted from the previously published article “Gialdini C, Chamillard M, Diaz V, Pasquale J, Thangaratinam S, Abalos E, Torloni MR, Betran AP. Evidence-based surgical procedures to optimize caesarean outcomes: an overview of systematic reviews. EClinicalMedicine. 2024 May 19; 72:102632” under the Creative Commons license: CC BY-NC-ND 4.0.”^11^

^2^For WMD, since there is no standard definition of narrow CI, in these situations we categorised the evidence as insufficient evidence.

^3^Substantially different: a large enough difference that it might affect a decision.

We extracted the meta-analysed data from the authors of the original reviews, but we did not extract the authors’ conclusions based on that data. This decision was made because systematic review authors may interpret the same data differently. Our aim is to compile the extensive body of available literature in a transparent manner, focusing on the numerical data itself, assessing its quality according to scientific standards for systematic reviews and randomised controlled trials, and interpreting the meta-analysed data based on these standards.

### Ethics

Ethical approval was not required because all data included are available in the public domain.

### Statistics

No statistical analyses were conducted in this overview.

### Role of the funding source

The funders of this study had no role in the overview design, data collection, data analysis, data interpretation, or writing of the manuscript.

All authors had full access to the data in the study; VD and APB had final responsibility for the decision to submit for publication.

## Results

**Fig. 1:**
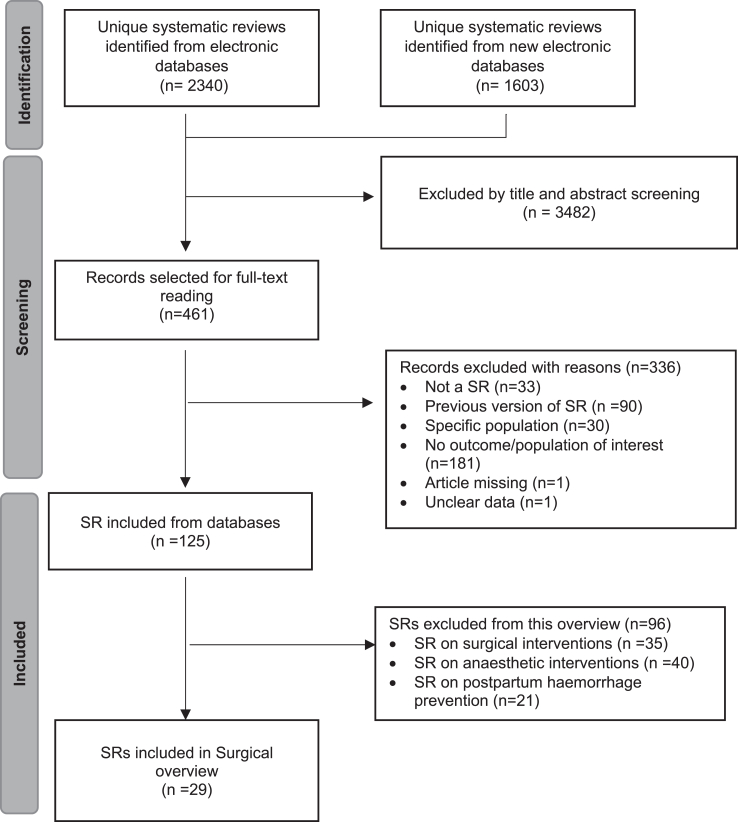
Flow diagram of the process of identification and selection of systematic reviews on medical procedures for caesarean section.

We identified 2340 unique records from the electronic databases in the original search (March 2020) and 1603 unique records in the updated search in January 2025. We excluded 3482 records by screening titles and abstracts and selected 461 for full text evaluation. After exclusions (Supplementary material 3), we included 29 SRs assessing different medical procedures at CS (Fig. 1).

### Description of included reviews

The 29 SRs^5^^,^^6^^,^^31^, ^32^, ^33^, ^34^, ^35^, ^36^, ^37^, ^38^, ^39^, ^40^, ^41^, ^42^, ^43^, ^44^, ^45^, ^46^, ^47^, ^48^, ^49^, ^50^, ^51^, ^52^, ^53^, ^54^, ^55^, ^56^, ^57^ (15 Cochrane and 14 non-Cochrane) were published in 2002–2024 and involved 408 unique RCTs with over 116,000 participants. Table 1 summarises the main characteristics of the included SRs (see Supplementary material 4 for details). Most of the SRs were conducted in the last six years (17/29), and 72.4% included studies conducted in LMICs (21/29). About two-thirds of the SRs (19/29) included both emergency or elective CSs and were rated as being of high methodological quality according to the AMSTAR 2 tool (18/29) (see Supplementary material 5).

**Table 1:** Main characteristic of included SRs.

Characteristic	N (%)	References
**Type of systematic review**		
Cochrane	15 (51.7%)	Abdel-Aleem 2014, Haas 2020, Jones 2021, Mackeen 2014, Mangesi 2002, Middleton 2021, Moore 2016, Motaze 2013, Nabhan 2016, Pereira Gomes Morais 2016, Smaill 2014, Sotiriadis 2021, Wetterslev 2015, Williams 2021, Yonemoto 2021
Non-Cochrane	14 (48.3%)	Abd-ElGawad 2020, Abuzaid 2024, Bolling 2018, Chaarani 2024, Chen 2024, Guo 2015, Hsu 2013, Kim 2021, Liu 2023, Markewi 2021, Menshawi 2018, Pinto Lopes 2016, Yang 2022, Zeng 2023
**Year of publication**		
Prior to 2013	1 (3.4%)	Mangesi 2002
2013–2017	11 (38%)	Abdel-Aleem 2014, Guo 2015, Hsu 2013, Mackeen 2014, Moore 2016, Motaze 2013, Nabhan 2016, Pereira Gomes Morais 2016, Pinto Lopes 2016, Smaill 2014, Wetterslev 2015
2018–2025	17 (58.6%)	Abd-ElGawad 2020, Abuzaid 2024, Bolling 2018, Chaarani 2024, Chen 2024, Haas 2020, Jones 2021, Kim 2021, Liu 2023, Markewi 2021, Menshawi 2018, Middleton 2021, Sotiriadis 2021, Williams 2021, Yang 2022, Yonemoto 2021, Zeng 2023
**Number of trials**		
0–5	4 (13.8%)	Abdel-Aleem 2014, Markewi 2021, Motaze 2013, Sotiriadis 2021
6–10	9 (31%)	Abd-ElGawad 2020, Abuzaid 2024, Albazee 2023, Chaarani 2024, Chen 2024, Kim 2021, Mackeen 2014, Mangesi 2002, Nabhan 2016
>10	16 (55.2%)	Bolling 2018, Guo 2015, Haas 2020, Hsu 2013, Jones 2021, Liu 2023, Middleton 2021, Moore 2016, Pereira Gomes Morais 2016, Pinto Lopes 2016, Smaill 2014, Wetterslev 2015, Williams 2021, Yang 2022, Yonemoto 2021, Zeng 2023
**Total N participants**		
<5000	19 (65.6%)	Abdel-Aleem 2014, Abd-ElGawad 2020, Abuzaid 2024, Chaarani 2024, Chen 2024, Guo 2015, Haas 2020, Hsu 2013, Kim 2021, Markewi 2021, Menshawi 2018, Middleton 2021, Moore 2016, Motaze 2013, Nabhan 2016, Pereira Gomes Morais 2016, Pinto Lopes 2016, Sotiriadis 2021, Yang 2022
5001–10,000	6 (20.7%)	Haas 2020, Jones 2021, Mackeen 2014, Wetterslev 2015, Williams 2021, Zeng 2023
>10,000	3 (10.3%)	Liu 2023, Smaill 2014, Yonemoto 2021
Unclear	1 (3.4%)	Bolling 2018
**Included trials from LMICs**		
Yes	21 (72.4%)	Abdel-Aleem 2014, Abd-ElGawad 2020, Abuzaid 2024, Bolling 2018, Chaarani 2024, Chen 2024, Haas 2020, Hsu 2013, Jones 2021, Liu 2023, Mackeen 2014, Markewi 2021, Moore 2016, Nabhan 2016, Pereira Gomes Morais 2016, Pinto Lopes 2016, Smaill 2014, Wetterslev 2015, Williams 2021, Yonemoto 2021, Zeng 2023
No	3 (10.3%)	Middleton 2021, Motaze 2013, Sotiriadis 2021
Unclear	5 (17.3%)	Guo 2015, Kim 2021, Mangesi 2002, Menshawi 2018, Yang 2022
**Type of caesarean section included in the systematic review**		
Elective only	3 (10.3%)	Menshawi 2018, Motaze 2013, Sotiriadis 2021
Elective and emergency	19 (65.5%)	Abdel-Aleem 2014, Abd-ElGawad 2020, Bolling 2018, Chaarani 2024, Chen 2024, Haas 2020, Jones 2021, Mackeen 2014, Mangesi 2002, Markewi 2021, Middleton 2021, Moore 2016, Nabhan 2016, Pereira Gomes Morais 2016, Pinto Lopes 2016, Smaill 2014, Williams 2021, Yang 2022, Zeng 2023
Unclear	7 (24.2%)	Abuzaid 2024, Guo 2015, Hsu 2013, Kim 2021, Liu 2023, Wetterslev 2015, Yonemoto 2021
**Quality of systematic review (AMSTAR 2 score)**		
Critically low	4 (13.7%)	Abuzaid 2024, Bolling 2018, Chen 2024, Hsu 2013
Low	7 (24.3%)	Guo 2015, Kim 2021, Liu 2023, Mangesi 2002, Menshawi 2018, Pinto Lopes 2016, Zeng 2023
Moderate	0 (0%)	
High	18 (62%)	Abdel-Aleem 2014, Abd-ElGawad 2020, Chaarani 2024, Haas 2020, Jones 2021, Mackeen 2014, Markewi 2021, Middleton 2021, Moore 2016, Motaze 2013, Nabhan 2016, Pereira Gomez Morais 2016, Smaill 2014, Sotiriadis 2021, Wetterslev 2015, Williams 2021, Yang 2022, Yonemoto 2021

### Summary of effects

We identified 512 procedure-outcome comparisons from the included SRs: 271 compared two different procedures, and 241 compared a procedure versus no treatment or placebo (NT/P). Among the 512 comparisons, there was insufficient or inconclusive evidence of any effect for 350 comparisons (68.3%). We found 97 comparisons (18.9%) with clear evidence of benefit (37 procedure versus procedure, 60 procedure versus NT/P), and 48 comparisons (9.3%) with evidence of a possible benefit (25 procedure versus procedure, 23 procedure versus NT/P). For 3 comparisons (0.6%), there was clear evidence of no difference of effect, and for 6 comparisons (1.2%), there was evidence of possible no difference of effect. Finally, for 4 comparisons (0.8%), there was clear evidence of harm (1 procedure versus procedure, 3 procedures versus NT/P), and for 4 comparisons (0.8%), there was evidence of possible harm (2 procedure versus procedure, 2 procedure versus NT/P) (Supplementary material 6).

Table 2, Table 3, Table 4, Table 5, Table 6 summarise the results for procedure-outcome comparisons with clear evidence of benefit, possible benefit, clear evidence of harm, possible harm, clear evidence of no difference, and possible evidence of no difference. Procedure-outcome comparisons with insufficient evidence or without SRs are presented in Supplementary material 7. For pre-specified 13 procedures, there were no SRs. We grouped the types of procedures into five major categories: pre-operative procedures, infection prevention, post-operative recovery, discharge and post discharge care, and other procedures. Supplementary material 8 provides the detailed list for all comparisons, outcomes, estimates, and references included in this overview.

**Table 2:** Preoperative preparation.

CI: Confidence interval; RCT: Randomised controlled trial.

**Table 3:** Procedures related to infection prevention.

CI: Confidence interval; RCT: Randomised controlled trial; CS: caesarean section.

**Table 4:** Procedures related to postoperative recovery.

CI: Confidence interval; MD: Mean difference; RCT: Randomised controlled trial; RR: Relative risk.

∗Lower scores indicate less pain and less distress.

**Table 5:** Discharge and post-discharge care.

CI: Confidence interval; RCT: Randomised controlled trial.

**Table 6:** Other medical procedures related to caesarean section.

CI: Confidence interval; MD: Mean difference; NICU: Neonatal intensive care unit; RCT: Randomised controlled trial; RR: Relative risk; SMD: Standardized mean difference.

### Pre-operative preparation

We could not find systematic reviews that included any of the pre-specified interventions related to pre-operative preparation (i.e., cardiovascular evaluation, laboratory tests, washing/bathing, pubic shaving, fasting, IV fluids administration) except for bladder emptying procedures (Table 2).

#### Bladder emptying and catheter removal procedures

The use of an indwelling bladder catheter, compared to no catheter, probably reduces the need for re-catheterization and may reduce bladder distension at the end of the operation, and urine retention. However, an indwelling catheter, compared to no catheter, probably increases the time of first voiding after CS and may increase pain or discomfort due to catheterization, and the time to patient ambulation. The use of an indwelling bladder catheter, compared to in–out urethral catheter before the operation, may reduce the need for re-catheterization. Immediate, compared to delayed, removal of urinary catheter after a CS probably reduces dysuria, urinary frequency, and significant bacteriuria. The reviews supporting evidence for procedures related to pre-operative preparation were of high quality according to the AMSTAR score tool. All comparisons and outcomes related to pre-operative preparation, with insufficient or uncertain evidence are listed in Supplementary material 7.

### Infection prevention procedures

#### Vaginal preparation

Several SRs assessed the effects of vaginal preparation with antiseptic solutions, compared to no preparation or saline preparation, on infectious outcomes in women undergoing any type of CS or only in women undergoing intrapartum CS (Table 3). Vaginal cleansing with a guanidine-based solution reduces post-caesarean endometritis, while preparation with a chlorhexidine-based solution may have a similar effect. Pre-operative vaginal cleansing with an iodine-based solution probably reduces post-caesarean endometritis and may reduce post-operative fever and SSI. Vaginal preparation with unspecified types of antiseptic solutions, compared to no preparation or saline preparation, probably reduces post-caesarean endometritis (any type of CS and only intrapartum CS), post-operative fever (any type of CS), and wound complications or endometritis (intrapartum CS), and it may reduce SSI and wound complications or endometritis (any type of CS). Most reviews supporting evidence for procedures related to vaginal preparation were of low quality according to the AMSTAR score tool. These results should be interpreted with caution. For all other comparisons and outcomes, the evidence is insufficient or uncertain (Supplementary material 7).

#### Prophylactic antibiotics

Evidence shows that antibiotic prophylaxis in general (all types), compared with no antibiotic prophylaxis, probably reduces post-caesarean endometritis, post-operative fever, SSI, serious maternal infectious complications, urinary tract infections, and duration of hospital stay. Cefazolin plus adjunctive prophylaxis, compared to cefazolin alone, reduce SSI. Minimally anti-staphylococcal cephalosporins C3 (3rd generation), compared with non-anti-staphylococcal penicillins P1 and P2 (natural and broad spectrum), probably increase endometritis. Antistaphylococcal penicillin P3 plus aminoglycaside A may increase post-operative fever (Table 3). Broad spectrum penicillin P2 plus antistaphylococcal penicillin P3 plus aminoglycaside A plus Nitroimidazol N, may increase costs when compared to Cephalosporin C3. Other specific types of antibiotics, compared to no antibiotic prophylaxis, probably reduce, or may reduce endometritis, post-operative fever, SSI, serious maternal infectious complications, maternal urinary tract infection, and duration of hospital stay.

Several SRs assessed timing of drug use on infectious morbidity and other outcomes. The administration of any antibiotics before cord clamping, compared to after cord clamping, reduces endometritis, SSI, serious maternal infectious complications, and duration of hospital stay, while it has no effect on NICU admission. Antibiotic prophylaxis in general (all types) after cord clamping, compared to no prophylaxis, probably reduces post-caesarean endometritis, post-operative fever, SSI, serious maternal infectious complications, urinary tract infections, and hospital stay.

Some SRs assessed the effects of prophylactic antibiotics in women undergoing different types of CS. Antibiotic prophylaxis in general (any type of antibiotic), compared to no prophylaxis, probably reduces, or may reduce post-caesarean endometritis, post-operative fever, SSI, serious maternal infectious complications, urinary tract infections, and hospital stay in both elective and non-elective caesareans.

Most reviews supporting evidence for procedures related to prophylactic antibiotics were of high quality according to the AMSTAR score tool. The effect of the procedures reported by systematic reviews with low or critically low quality AMSTAR score should be interpreted with caution.

All identified comparisons and outcomes related to infection prevention procedures, with insufficient or uncertain evidence are listed in Supplementary material 7.

### Post-operative recovery

Early oral intake, compared to delayed intake, shortens the time to initiation of ambulation, and probably shortens the time for bowel sounds to return, time to stop IV fluids, time to urinary catheter removal, time to first food ingestion, and time to first breastfeeding. Stratified by type of anaesthesia there are no changes in results showing early oral intake clear evidence of benefits in caesarean section all types of anaesthesia (Table 4). Chewing gum in the post-operative period probably reduces the risk of ileus and may shorten the time for bowel sounds to return, the time to passing flatus, and duration of hospital stay. The use of abdominal binders reduces pain scores after 48 h and probably reduces pain scores after 24 h and distress scores after 24 and 48 h (Table 4).

The reviews supporting evidence for procedures related to post-operative recovery varied from critically low to high quality according to the AMSTAR score tool. The effect of the procedures reported by systematic reviews with low or critically low quality AMSTAR score should be interpreted with caution.

All identified comparisons and outcomes related to post-operative recovery, with insufficient or uncertain evidence are listed in Supplementary material 7.

### Discharge and post-discharge care

#### Time of discharge

Early compared to standard discharge may not have an effect on breastfeeding at 6 weeks but probably increases exclusive or partial breastfeeding at 12 weeks and may decrease women reporting health problems in the first 6 weeks postpartum. However, early discharge probably increases readmission for neonatal morbidity within 28 days, in particular when discharge occurs less than 24 h after birth (Table 5). The reviews supporting evidence for procedures related to time of discharge were of high quality according to the AMSTAR score tool.

#### Post-discharge visits

Home visits compared to facility visits probably have no effect on the prevalence of exclusive breastfeeding up to 6 weeks but may increase maternal satisfaction with postnatal care. Home visits, compared to no home visits, may increase exclusive breastfeeding up to 6 weeks and may have no effect on severe maternal morbidity and back pain up to 6 weeks. Home visits, compared to telephone consultations, probably have no effect on neonatal morbidity up to 28 days (Table 5).

Schedules involving more, compared to fewer, home visits may increase exclusive breastfeeding up to 6 weeks and up to 6 months, but may have no effect on infant respiratory tract infection up to 6 weeks, infant diarrhoea or maternal satisfaction with postpartum care. Schedules involving four or more home visits, compared with less than four visits, may increase exclusive breastfeeding at 6 weeks and maternal satisfaction with postpartum care (Table 5). The reviews supporting evidence for procedures related to post-discharge visits were of high quality according to the AMSTAR score tool.

All identified comparisons and outcomes related to discharge and post-discharge care, with insufficient or uncertain evidence are listed in Supplementary material 7. We could not find any SR on other pre-specified interventions related to discharge and post-discharge care (wound care, time to stich removal, time to resume physical activity, time to resume sexual activity).

### Other procedures

Pre-operative informative videos probably reduce maternal post-operative anxiety (Table 6). These reviews were of critically low quality according to the AMSTAR score tool and results should be interpreted with caution. For term pregnancies, the administration of antenatal corticosteroids (betamethasone), compared with usual care, probably reduces neonatal admission for respiratory morbidity and transient neonatal tachypnoea, and may reduce NICU admission for respiratory morbidity and NICU length of stay. These reviews were of high quality according to the AMSTAR score tool (Table 6).

All identified comparisons and outcomes related to other procedures, with insufficient or uncertain evidence are listed in Supplementary material 7.

## Discussion

This overview of systematic reviews of RCT synthesises the scientific literature on the effects of multiple medical procedures commonly used in caesarean births. We identified 29 SRs encompassing evidence from 408 unique RCT including more than 116,000 women undergoing a CS. Using a pre-specified list of procedures and outcomes, we were able to assess 512 procedure-outcome pair comparisons which were classified according to their effectiveness and certainty of evidence. These simple categories are useful for clinicians and policy-makers to inform practices and policies.

An important concern emerging from this overview is that for 68.3% of the procedure-outcome comparisons identified (350/512), conclusions cannot be drawn due to insufficient evidence or very low certainty of evidence. These included critical outcomes such as maternal or neonatal sepsis for several interventions. Similarly, there is insufficient evidence to draw conclusions on several outcomes related to the administration of corticosteroids in term caesarean sections, thromboprophylaxis, and the timing of fluids and food intake. The lack of SRs summarizing the effectiveness of pre-operative evaluations, including cardiovascular assessment, and laboratory tests before conducting a CS is an important gap in the literature that can have a considerable impact on costs. Additionally, there is a lack of SRs to inform recommendations on some procedures that may not be viewed as critical for healthcare providers but are important to a woman as she prepares for a CS (e.g., pre-operative bathing, hair shaving, fasting), after she goes home (wound care, timing of stitch removal) or when she returns to her normal activities after giving birth (e.g., time to resume physical activity or sexual intercourse). The importance of making evidence-based recommendations for these aspects should not be underestimated, as they may have a significant impact on women’s quality of life and mental health.^58^, ^59^, ^60^ The lack of SRs on these topics means that clinicians must base their recommendations on personal experience and preferences. These gaps underscore the need for rigorous SRs to support guidelines to standardise care and improve safety and health in caesarean births.

For some procedures, such as the optimal time of discharge and post-partum visits, evidence shows variable effects depending on the outcome. Drawing conclusions about these procedures is challenging because of methodological issues, such as the low certainty of the evidence for many comparisons, and the heterogeneity of interventions included, as well as ethical considerations regarding the potentially competing needs of the mother and baby.

Limitations of the available evidence may help to explain the lack of international recommendations in this area, despite the large volume of studies assessing medical procedures to improve safety and enhance the experience of and recovery after a CS. For example, WHO has only two recommendations on medical procedures related to CS included in this overview, namely, vaginal preparation with chlorhexidine gluconate or povidone iodine immediately before CS, and the administration of antibiotics for infection prevention at CS.^61^^,^^62^ Two additional recommendations by WHO, namely, choice of antiseptic agent and method of application for pre-operative skin preparation for women undergoing CS were included in the previously published surgical overview.^11^^,^^63^

A strong point of this overview is its novelty. To the best of our knowledge, this is the first overview of SRs of procedures related to the medical aspects of CS. In 2018, Cochrane published an overview of systematic reviews on intraoperative interventions to prevent surgical site infections across various types of surgery.^64^ The conclusions of this overview regarding the use of prophylactic antibiotics and vaginal preparation during caesarean section align with the findings of our own overview. Additionally, the review followed rigorous methodological standards, including protocol registration with pre-specified procedures and outcomes before commencing the overview, and the involvement of two independent reviewers in study selection, data extraction, quality assessment, and synthesis of the evidence. We also developed a sensitive search strategy which was run on several electronic databases without date or language restrictions to retrieve relevant Cochrane and non-Cochrane reviews, and we applied pre-specified rules to handle duplicate reviews and comparisons to avoid double inclusion of studies. Finally, by presenting the effectiveness of each procedure for each outcome independently along with the AMSTAR score assessment of the SR, we offer readers the possibility of making more tailored and context-specific decisions according to their priorities.

Our overview has some limitations. Since we did not conduct a comprehensive search for grey literature, we may have missed SRs not reported in scientific publications. Limitations pertaining to the SRs included in this overview should be kept in mind by the readers when interpreting our main results. Also, while we acknowledge that a trustworthiness screening tool for trials proposed by Cochrane would improve the credibility of the review findings, it is a relatively recent tool, and therefore it was not used in most systematic reviews included in this overview. However, its use should be encouraged in future reviews to enhance the reliability and trustworthiness of the evidence. Except for antibiotic prophylaxis, and here to a limited extent, we could not provide results based on the type of CS, as most SRs included trials with a mix of women undergoing both elective and emergency caesareans and provided pooled estimates without subgroup analyses. Additionally, due to insufficient information in the SRs, we were unable to evaluate the differential effects of interventions based on the number of previous CS, gestational age, indications, or maternal health status.

This review focuses on women undergoing a CS in the absence of major complications or risk factors (e.g., placenta previa or accreta, obesity, diabetes) that may require special procedures. This is an area for future overviews of SRs. The evidence related to PPH prevention in women undergoing a CS will be reported in a separate manuscript.

Future research should address the gaps identified in this overview on the effects of currently used medical procedures on relevant maternal and perinatal outcomes to ensure that evidence-based CS are accessible to all women who need this operation, meeting their specific needs while optimizing health system resources. It is also essential to assess the value of new medical procedures for CS that will probably be devised and tested in the future, to ensure their effectiveness, cost-effectiveness, risks, acceptability and added value, before their introduction into practice.

The results of this overview highlight the urgent need for the development of internationally accepted guidelines for medical procedures used in CS. Standardised and evidence-based medical procedures play a crucial role in reducing the risk of CS complications and maintaining a high standard of care for all patients managed in various settings and by different types of healthcare providers. These procedures streamline the operation, making it more efficient. This efficiency can lead to quicker recovery for patients, and more effective use of hospital resources. Clear guidelines also facilitate the training and education of medical staff, ensuring that all team members are well-versed in best practices and can perform their roles effectively, particularly in emergency situations. By implementing and adhering to standardised procedures and guidelines, healthcare providers can ensure safer, more effective, and more equitable care for women undergoing CS.

## Contributors

VD, CG, MC, JP, MRT and APB conceived the study. VD, CG, MC, JP and APB developed the data extraction forms and performed screening and data collection. VD, CG, MC, JP, MRT and APB drafted the outline of the manuscript and wrote the first draft. VD, CG, MRT and APB contributed substantially to the writing of the final version of the manuscript. GC, AP, MRT contributed to the revision of the final manuscript. VD, CG, MC, APB and MRT developed the Supplementary materials, tables, and figures. All authors read and approved the final manuscript. VC and CG have access to and verified the underlying study data.

## Data sharing statement

The authors confirm that the data supporting the findings of this study are available within the article and its Supplementary materials.

## Declaration of interests

All authors declare no competing interests.

## Appendix ASupplementary data

Supplementary materials 1 to 8

Abstract in Spanish

Abstract in Portuguese

Abstract in French
