# Supplementary material for: Evidence-based medical procedures to optimise caesarean outcomes: an overview of systematic reviews
Source: eClinicalMedicine. 2025 Apr 30;83:103212. doi: 10.1016/j.eclinm.2025.103212 (PMC12076788; doi:10.1016/j.eclinm.2025.103212)
Supplement: Supplementary materials 1 to 8 [file mmc1.pdf]

Supplementary material 1. Prespecified interventions

Categories	Pre-specified interventions
Pre-operative evaluation	1. Preoperative Cardiovascular evaluation<br>2. Preoperative Laboratory tests<br>3. Preoperative Washing/ Bathing<br>4. Preoperative Shaving<br>5. Preoperative Fasting<br>6. Preoperative IV fluids
Pre-operative preparation	7. Bladder emptying
Infection prevention	8. Pre-operative vaginal preparation<br>9. Prophylactic antibiotics
Post-operative recovery	10. Time to fluids and food<br>11. Time to mobilization<br>12. Wound care in hospital<br>13. Use of abdominal binders
Discharge and post-discharge care	14. Time of hospital discharge<br>15. Time to stich removal<br>16. Wound care at home<br>17. Post-discharge controls<br>18. Time to resume physical activity<br>19. Time to resume sexual activity
Other aspects	20. Corticosteroids for preventing. neonatal distress syndrome<br>21. Skin to skin contact<br>22. Companionship during caesarean section<br>23. Education/ information<br>24. Thromboprophylaxis<br>25. Use of protocols for caesarean section

Supplementary material 2

OVERVIEW OF INTERVENTIONS FOR SAFE CAESAREAN SECTION SEARCH SATRATEGY

cesarean OR caesarean OR c-section OR "C section" OR "abdominal deliver" OR "surgical deliver*" OR "Cesarean Section"[Mesh] OR C-section*[TW] OR Postcesarean*[TW] OR Postcaesarean*[TW]*
AND
(Systematic[ti] and review[ti]) OR Systematic overview*[ti] OR Cochrane review*[ti] OR Systemic review*[ti] OR Scoping review[ti] OR scoping literature review [ti] OR mapping review [ti] OR Umbrella review [ti] OR Review of reviews [ti] OR overview of reviews [ti] OR meta-review[ti] OR meta-synthesis [ti] OR metasynthesis [ti] OR meta-ethnography [ti] OR integrative review [ti] OR integrated review [ti] OR integrative overview[ti] OR quantitative review[ti] OR quantitative synthesis [ti] OR research synthesis[ti] OR systematic literature search[ti] OR systematic literature research[ti] OR meta-analyses [ti] OR metaanalyses [ti] OR metaanalysis [ti] OR meta-analysis [ti] OR meta-analytic review [ti] OR meta-analytical review [ti] OR meta-analysis[pt] OR (search* [tiab] OR medline [tiab] OR pubmed [tiab] OR embase [tiab] OR Cochrane [tiab] OR scopus [tiab] or web of science [tiab] OR sources of information [tiab] OR data sources [tiab] OR following databases [tiab]) AND (study selection [tiab] OR selection criteria [tiab] OR eligibility criteria [tiab] OR inclusion criteria [tiab] OR exclusion criteria [tiab]) OR Systematic review [pt]*

Supplementary material 3

REFERENCES FOR EXCLUDED SRs AND INDIVIDUAL REASON FOR EXCLUSION

TITLE	AUTHORS	REASON FOR EXCLUSION
Transversus abdominis plane block for postoperative analgesia after Caesarean delivery performed under spinal anaesthesia? A systematic review and meta-analysis.	Abdallah FW; Halpern SH; Margarido CB	SR on anaesthetic interventions
Regional versus general anaesthesia for caesarean section.	Afolabi BB; Lesi FE; Merah NA	SR on anaesthetic interventions
Barbed vs conventional suture at cesarean delivery: A systematic review and meta-analysis	Agarwal	SR on surgical interventions
Techniques and materials for skin closure in caesarean section.	Alderdice F; McKenna D; Dornan J	Previous version of SR
Different classes of antibiotics given to women routinely for preventing infection at caesarean section.	Alfirevic Z; Gyte GM; Dou L	Previous version of SR
P6 stimulation for the prevention of nausea and vomiting associated with cesarean delivery under neuraxial anesthesia: a systematic review of randomized controlled trials.	Allen TK; Habib AS	Not a SR
Efficacy of perioperative ce**furoxime** as a prophylactic antibiotic in women requiring caesarean section: A systematic review.	Alrammaal HH; Batchelor HK; Morris RK; Chong HP	Not a SR
Techniques and materials for closure of the abdominal wall in caesarean section	Elizabeth R Anderson, Simon Gates	SR on surgical interventions
Local anaesthetics and regional anaesthesia for preventing chronic pain after surgery.	Andreae MH; Andreae DA	SR on anaesthetic interventions
Methods of delivering the placenta at caesarean section	Rose I Anorlu , Babalwa Maholwana, G Justus Hofmeyr	SR on surgical interventions
Timing of administration of prophylactic antibiotics for caesarean section: a systematic review and meta-analysis.	Baaqeel H; Baaqeel R	Previous version of SR
Prophylactic Dose of Oxytocin for Uterine Atony during Caesarean Delivery: A Systematic Review.	Baliuliene V; Vitartaite M; Rimaitis K	SR on postpartum haemorrhage prevention
Effect of Perioperative Active Body Surface Warming Systems on Analgesic and Clinical Outcomes: A Systematic Review and Meta-analysis of Randomized Controlled Trials.	Balki I; Khan JS; Staibano P; Duceppe E; Bessissow A; Sloan EN; Morley EE; Thompson AN; Devereaux B; Rojas C; Siddiqui N; Sessler DI; Devereaux PJ	Specific population
Caesarean section wound infiltration with local anaesthesia for postoperative pain relief - any benefit?	Bamigboye AA; Hofmeyr GJ	SR on anaesthetic interventions
Closure versus non-closure of the peritoneum at caesarean section.	Bamigboye AA; Hofmeyr GJ	SR on surgical interventions
Non-closure of peritoneal surfaces at caesarean section--a systematic review.	Bamigboye AA; Hofmeyr GJ	Previous version of SR
Uterine externalization versus in situ repair of hysterotomy during cesarean delivery: a systematic review, equivalence meta-analysis, and trial sequential analysis.	Bhat A; Jaffer D; Keasler P; Kamath K; Kelly J; Singh PM	Previous version of SR

[Antimicrobial prophylaxis for caesarean delivery: before or after cord clamping? A meta-analysis].	Boselli E; Bouvet L; RimmelÃ© T; Chassard D; Allaouchiche B	Previous version of SR
Intraoperative prophylactic and therapeutic non-invasive ventilation: a systematic review.	Cabrini L; Nobile L; Plumari VP; Landoni G; Borghi G; Mucchetti M; Zangrillo A	No outcomes/population of interest
Vaginal Cleansing Before Cesarean Delivery: A Systematic Review and Meta-analysis.	Caissutti C; Saccone G; Zullo F; Quist-Nelson J; Felder L; Ciardulli A; Berghella V	Previous version of SR
Evidence-Based Bundles and Cesarean Delivery Surgical Site Infections: A Systematic Review and Meta-analysis.	Carter EB; Temming LA; Fowler S; Eppes C; Gross G; Srinivas SK; Macones GA; Colditz GA; Tuuli MG	Not a SR
Guidelines for intraoperative care in cesarean delivery: Enhanced Recovery After Surgery Society Recommendations (Part 2).	Caughey AB; Wood SL; Macones GA; Wrench IJ; Huang J; Norman M; Pettersson K; Fawcett WJ; Shalabi MM; Metcalfe A; Gramlich L; Nelson G; Wilson RD	Not a SR
Scalpel versus electrosurgery for major abdominal incisions	Charoenkwan	SR on surgical interventions
Supplemental oxygen for caesarean section during regional anaesthesia.	Chatmongkolchart S; Prathep S	SR on anaesthetic interventions
Suture closure of subcutaneous fat and wound disruption after cesarean delivery: a meta-analysis.	Chelmow D; Rodriguez EJ; Sabatini MM	Previous version of SR
To close or not to close? A systematic review and a meta-analysis of peritoneal non-closure and adhesion formation after caesarean section.	Cheong YC; Premkumar G; Metwally M; Peacock JL; Li TC	Previous version of SR
Techniques for preventing hypotension during spinal anaesthesia for caesarean section.	Chooi C; Cox JJ; Lumb RS; Middleton P; Chemali M; Emmett RS; Simmons SW; Cyna AM	Previous version of SR
Chewing gum improves postoperative recovery of gastrointestinal function after cesarean delivery: a systematic review and meta-analysis of randomized trials.	Ciardulli A; Saccone G; Di Mascio D; Caissutti C; Berghella V	Previous version of SR
Maternal position during caesarean section for preventing maternal and neonatal complications.	Cluver C; Novikova N; Hofmeyr GJ; Hall DR	Previous version of SR
Outcomes of second stage cesarean section following the use of a fetal head elevation device: A systematic review and meta-analysis.	Conde-Agudelo A; Nieto A; Rosas-Bermudez A; Romero R	Previous version of SR
A systematic review of the effects of adding neostigmine to local anesthetics for neuraxial administration in obstetric anesthesia and analgesia.	Cossu AP; De Giudici LM; Piras D; Mura P; Scanu M; Cossu M; Saba M; Finco G; Brazzi L	SR on anaesthetic interventions
Management of Impacted Fetal Head at Caesarean Birth	Katie R Cornthwaite a,⁎, Rachna Bahl b, Katherine Lattey c, Tim Draycott d	SR on surgical interventions
Chewing gum in preventing postoperative ileus in women undergoing caesarean section: a systematic review and meta-analysis of randomised controlled trials.	Craciunas L; Sajid MS; Ahmed AS	Previous version of SR
Intrathecal clonidine as an adjuvant for neuraxial anaesthesia during caesarean delivery: a systematic review and meta-analysis of randomised trials.	Crespo S; Dangelser G; Haller G	SR on anaesthetic interventions
Techniques for preventing hypotension during spinal anaesthesia for caesarean section.	Cyna AM; Andrew M; Emmett RS; Middleton P; Simmons SW	SR on anaesthetic interventions
Intraoperative and postoperative analgesic efficacy and adverse	Dahl JB; Jeppesen IS; JÃ¸rgensen H; Wetterslev J; MÃ¸iniche S	SR on anaesthetic interventions

effects of intrathecal opioids in patients undergoing cesarean section with spinal anesthesia: a qualitative and quantitative systematic review of randomized controlled trials.		
Evidence-based surgery for cesarean delivery: an updated systematic review.	Dahlke JD; Mendez-Figueroa H; Rouse DJ; Berghella V; Baxter JK; Chauhan SP	Not a SR
Preventing Hypothermia during Cesarean Birth: An Integrative Review.	Dendis M; Hooven K	SR on anaesthetic interventions
Outcomes of second stage cesarean section following the use of a fetal head elevation device: A systematic review and meta-analysis.	Di Girolamo R; Galliani C; Buca D; Liberati M; D'Antonio F	Not a SR
The efficacy of abdominal binders in reducing postoperative pain and distress after cesarean delivery: A meta-analysis of randomized controlled trials.	Di Mascio D; Caruso G; Prata G; Saccone G; Terrin G; Giancotti A; Brunelli R; Muzii L; Benedetti Panici P; Di Donato V	Previous version of SR
Risk of Cesarean scar defect following single- vs double-layer uterine closure: systematic review and meta-analysis of randomized controlled trials.	Di Spiezio Sardo A; Saccone G; McCurdy R; Bujold E; Bifulco G; Berghella V	Previous version of SR
Surgical techniques for uterine incision and uterine closure at the time of caesarean section.	Dodd JM; Anderson ER; Gates S	Previous version of SR
Tocolysis for assisting delivery at caesarean section.	Dodd JM; Reid K	Previous version of SR
Dressings for the prevention of surgical site infection	Jo C Dumville, Trish A Gray, Catherine J Walter, Catherine A Sharp , Tamara Page , Rhiannon Macefield , Natalie Blencowe , Thomas Kg Milne , Barnaby C Reeves, Jane Blazeb	SR on surgical interventions
Intra-abdominal saline irrigation at cesarean section: a systematic review and meta-analysis review and meta-analysis	Ahizechukwu Chigoziem Eke, Ghadear Hussein Shukr, Tina Taissir Chaalan, Sereen Khaled Nashif, George Uchenna Eleje	SR on surgical interventions
Techniques for preventing hypotension during spinal anaesthesia for caesarean section.	Emmett RS; Cyna AM; Andrew M; Simmons SW	SR on anaesthetic interventions
Techniques for preventing hypotension during spinal anaesthesia for caesarean section.	Emmett RS; Cyna AM; Andrew M; Simmons SW	SR on postpartum haemorrhage prevention
Single versus two layer suturing for closing the uterine incision at caesarean section.	Enkin MW; Wilkinson C	Previous version of SR
[Efficacy and safety of tranexamic acid administration for the prevention and/or the treatment of post-partum haemorrhage: a systematic review with meta-analysis].	Faraoni D; Carlier C; Samama CM; Levy JH; Ducloy-Bouthors AS	SR on postpartum haemorrhage prevention
Are prophylactic adjunctive macrolides efficacious against caesarean section surgical site infection: A systematic review and meta-analysis.	Farmer N; Hodgetts-Morton V; Morris RK	Previous version of SR
Epidemiology, prevention and management of early postpartum hemorrhage - a systematic review.	Feduniw S; Warzecha D; Szymusik I; Wielgos M	SR on postpartum haemorrhage prevention
Prevention of hypotension after spinal anaesthesia for caesarean section: a systematic review and network meta-analysis of randomised controlled trials.	Fitzgerald JP; Fedoruk KA; Jadin SM; Carvalho B; Halpern SH	SR on postpartum haemorrhage prevention

Safety and efficacy of tranexamic acid for prevention of obstetric haemorrhage: an updated systematic review and meta-analysis.	Franchini M; Mengoli C; Cruciani M; Bergamini V; Presti F; Marano G; Pupella S; Vaglio S; Masiello F; Veropalumbo E; Piccinini V; Pati I; Liumbruno GM	Previous version of SR
[Continuous infusion of local anesthetic at the site of the abdominal surgical wound for postoperative analgesia: a systematic review].	Fustran Guerrero N; Dalmau LlitjÃ³s A; SabatÃ© Pes A	Not a SR
Wound drainage for caesarean section.	Gates S; Anderson ER	Previous version of SR
Prophylaxis for venous thromboembolic disease in pregnancy and the early postnatal period.	Gates S; Brocklehurst P; Davis LJ	Previous version of SR
Effect of negative-pressure wound therapy on wound complications in obese women after caesarean birth: a systematic review and meta-analysis.	Gillespie BM; Thalib L; Ellwood D; Kang E; Mahomed K; Kumar S; Chaboyer W	Specific population
Effects of colloid preload on the incidence of hypotension in spinal anesthesia for cesarean section: a systematic review and meta-analysis.	Gong RS; Liu XW; Li WX; Zhao J	Previous version of SR
Interventions for preventing nausea and vomiting in women undergoing regional anaesthesia for caesarean section.	Griffiths JD; Gyte GM; Paranjothy S; Brown HC; Broughton HK; Thomas J	SR on anaesthetic interventions
The safety of early postpartum discharge: a review and critique.	Grullon KE; Grimes DA	Previous version of SR
Prophylactic negative pressure wound therapy on surgical site infection in obese women after cesarean section: A systematic review and meta-analysis.	Guo C; Cheng T; Li J	Specific population
Different classes of antibiotics given to women routinely for preventing infection at caesarean section.	Gyte GM; Dou L; Vazquez JC	Previous version of SR
Vaginal preparation with antiseptic solution before cesarean section for preventing postoperative infections.	Haas DM; Morgan Al Darei S; Contreras K	Previous version of SR
Vaginal preparation with antiseptic solution before cesarean section for preventing postoperative infections.	Haas DM; Morgan S; Contreras K	Previous version of SR
Vaginal preparation with antiseptic solution before cesarean section for preventing postoperative infections.	Haas DM; Morgan S; Contreras K	Previous version of SR
Vaginal preparation with antiseptic solution before cesarean section for preventing postoperative infections.	Haas DM; Morgan S; Contreras K; Enders S	Previous version of SR
Skin preparation for preventing infection following caesarean section.	Hadiati DR; Hakimi M; Nurdiati DS	SR on surgical interventions
Skin preparation for preventing infection following caesarean section.	Hadiati DR; Hakimi M; Nurdiati DS; da Silva Lopes K; Ota E	Previous version of SR
Skin preparation for preventing infection following caesarean section.	Hadiati DR; Hakimi M; Nurdiati DS; Ota E	Previous version of SR
Staples versus subcuticular suture for cesarean skin closure in obese women: A systematic review and meta-analysis.	Han D; Feng L; Xu L; Li C; Zhang Q	Specific population
Intravenous ketamine during spinal and general anaesthesia for caesarean section: systematic review and meta-analysis.	Heesen M; BÃ¶hmer J; Brinck EC; Kontinen VK; KlÃ¶hr S; Rossaint R; Straube S	Previous version of SR

Is general anaesthesia for caesarean section associated with postpartum haemorrhage? Systematic review and meta-analysis.	Heesen M; Hofmann T; KlÃ¶hr S; Rossaint R; van de Velde M; Deprest J; Straube S	Previous version of SR
Prophylactic phenylephrine for caesarean section under spinal anaesthesia: systematic review and meta-analysis.	Heesen M; KÃ¶hr S; Rossaint R; Straube S	No outcomes/population of interest
Maternal and foetal effects of remifentanil for general anaesthesia in parturients undergoing caesarean section: a systematic review and meta-analysis.	Heesen M; KlÃ¶hr S; Hofmann T; Rossaint R; Devroe S; Straube S; Van de Velde M	Specific population
Concerning the timing of antibiotic administration in women undergoing caesarean section: a systematic review and meta-analysis.	Heesen M; KlÃ¶hr S; Rossaint R; Allegaert K; Allegeaert K; Deprest J; Van de Velde M; Straube S	Previous version of SR
Prevention of Spinal Anesthesia-Induced Hypotension During Cesarean Delivery by 5-Hydroxytryptamine-3 Receptor Antagonists: A Systematic Review and Meta-analysis and Meta-regression.	Heesen M; Klimek M; Hoeks SE; Rossaint R	Previous version of SR
Prophylactic subcutaneous drainage for prevention of wound complications after cesarean delivery--a metaanalysis.	Hellums EK; Lin MG; Ramsey PS	Previous version of SR
Hyperbaric versus plain bupivacaine for spinal anesthesia for cesarean delivery.	Heng Sia AT; Tan KH; Sng BL; Lim Y; Chan ESY; Siddiqui FJ	Previous version of SR
Gum chewing and gastrointestinal function following caesarean delivery: a systematic review and meta-analysis.	Hochner H; Tenfelde SM; Abu Ahmad W; Liebergall-Wischnitzer M	Previous version of SR
Techniques for caesarean section	Hofmeyr	
Antibiotic prophylaxis regimens and drugs for cesarean section.	Hopkins L; Smaill F	Previous version of SR
Information for pregnant women about caesarean birth.	Horey D; Weaver J; Russell H	No outcomes/population of interest
Hypnotic agents for induction of general anesthesia in cesarean section patients: A systematic review and meta-analysis of randomized controlled trials.	Houthoff Khemlani K; Weibel S; Kranke P; Schreiber JU	Specific population
Effect of misoprostol versus oxytocin during caesarean section: a systematic review and meta-analysis.	Hua J; Chen G; Xing F; Scott M; Li Q	Previous version of SR
Usefulness of chewing gum for recovering intestinal function after cesarean delivery: A systematic review and meta-analysis of randomized controlled trials.	Huang HP; He M	Previous version of SR
Extra-abdominal versus intra-abdominal repair of the uterine incision at caesarean section	Jacobs	SR on surgical interventions
Implementation of vaginal preparation prior to caesarean section.	Jakes AD; Bell A; Chiwera L; Lloyd J	Not a SR
Carbetocin for the prevention of postpartum hemorrhage: a systematic review and meta-analysis of randomized controlled trials.	Jin B; Du Y; Zhang F; Zhang K; Wang L; Cui L	Previous version of SR
Single injection Quadratus Lumborum block for postoperative analgesia in adult surgical population: A systematic review and meta-analysis.	Jin Z; Liu J; Li R; Gan TJ; He Y; Lin J	SR on anaesthetic interventions

[Early post-partum discharge: systematic review of the literature].	Jonguitud-Aguilar A; Tomasso G; Cafferatta ML	Not a SR
A scoping review of maternal antibiotic prophylaxis in low- and middle-income countries: Comparison to WHO recommendations for prevention and treatment of maternal peripartum infection.	Jury I; Thompson K; Hirst JE	Not a SR
Efficacy of carbetocin in the prevention of postpartum hemorrhage: a systematic review and Bayesian meta-analysis of randomized trials.	Kalafat E; Gokce A; O'Brien P; Benlioglu C; Koc A; Karaaslan O; Khalil A	SR on postpartum haemorrhage prevention
Does tranexamic acid prevent postpartum haemorrhage? A systematic review of randomised controlled trials.	Ker K; Shakur H; Roberts I	SR on postpartum haemorrhage prevention
Suture type for hysterotomy closure: a systematic review and meta-analysis of randomized controlled trials	Kavisha Khanuja , Julia Burd, Pinar Ozcan , David Peleg, Gabriele Saccone , Vincenzo Berghella	SR on surgical interventions
Combined spinal-epidural vs. spinal anaesthesia for caesarean section: meta-analysis and trial-sequential analysis.	Klimek M; Rossaint R; van de Velde M; Heesen M	SR on anaesthetic interventions
A meta-analysis of the effect of inspired oxygen concentration on the incidence of surgical site infection following cesarean section.	Klingel ML; Patel SV	No outcomes/population of interest
Evidence-based value of subcutaneous surgical wound drainage: the largest systematic review and meta-analysis.	Kosins AM; Scholz T; Cetinkaya M; Evans GRD	Previous version of SR
A systematic review of the cost-effectiveness of uterotonic agents for the prevention of postpartum hemorrhage.	Lawrie TA; RogoziÅ„ska E; Sobiesuo P; Vogel JP; Ternent L; Oladapo OT	No outcomes/population of interest
A dose-response meta-analysis of prophylactic intravenous ephedrine for the prevention of hypotension during spinal anesthesia for elective cesarean delivery.	Lee A; Ngan Kee WD; Gin T	Previous version of SR
A quantitative, systematic review of randomized controlled trials of ephedrine versus phenylephrine for the management of hypotension during spinal anesthesia for cesarean delivery.	Lee A; Ngan Kee WD; Gin T	Previous version of SR
Prophylactic ephedrine prevents hypotension during spinal anesthesia for Cesarean delivery but does not improve neonatal outcome: a quantitative systematic review.	Lee A; Ngan Kee WD; Gin T	Previous version of SR
Effect of intravenous dexmedetomidine and remifentanil on neonatal outcomes after caesarean section under general anaesthesia: A systematic review and meta-analysis.	Lee M; Kim H; Lee C; Kang H	Specific population
Mechanical dilatation of the cervix during elective caesarean section before the onset of labour for reducing postoperative morbidity	Liabsuetrakul	SR on surgical interventions
Is prophylactic tranexamic acid administration effective and safe for postpartum hemorrhage prevention?: A systematic review and meta-analysis.	Li C; Gong Y; Dong L; Xie B; Dai Z	Previous version of SR

Is routine indwelling catheterisation of the bladder for caesarean section necessary? A systematic review.	Li L; Wen J; Wang L; Li YP; Li Y	Previous version of SR
Comparing prophylactic use of cefazolin for SSI in cesarean section: a systematic review and meta-analysis.	Li M; Shi B; Ma J; Peng X; Shi J	Previous version of SR
Subarachnoid and epidural dexmedetomidine for the prevention of post-anesthetic shivering: a meta-analysis and systematic review.	Li YZ; Jiang Y; Lin H; Yang XP	SR on anaesthetic interventions
Mechanical dilatation of the cervix at non-labour caesarean section for reducing postoperative morbidity.	Liabsuetrakul T; Peeyananjarassri K	Previous version of SR
Effect of Intravenous Ketamine on Hypocranial Pressure Symptoms in Patients with Spinal Anesthetic Cesarean Sections: A Systematic Review and Meta-Analysis.	Liang X; Yang X; Liang S; Zhang Y; Ding Z; Guo Q; Huang C	No outcomes/population of interest
Shivering prevention and treatment during cesarean delivery under neuraxial anesthesia: a systematic review.	Liu J; Wang Y; Ma W	SR on anaesthetic interventions
Intraoperative interventions for preventing surgical site infection: an overview of Cochrane Reviews.	Liu Z; Dumville JC; Norman G; Westby MJ; Blazeby J; McFarlane E; Welton NJ; O'Connor L; Cawthorne J; George RP; Crosbie EJ; Rithalia AD; Cheng HY	Not a SR
Techniques and materials for skin closure in caesarean section	A Dhanya Mackeen, Vincenzo Berghella, Mie-Louise Larsen	SR on surgical interventions
Suture Compared with Staples for Skin Closure After Cesarean Delivery	A Dhanya Mackeen, Maranda V Sullivan, Meike Schuster, Vincenzo Berghella	SR on surgical interventions
Guidelines for postoperative care in cesarean delivery: Enhanced Recovery After Surgery (ERAS) Society recommendations (part 3).	Macones GA; Caughey AB; Wood SL; Wrench IJ; Huang J; Norman M; Pettersson K; Fawcett WJ; Shalabi MM; Metcalfe A; Gramlich L; Nelson G; Wilson RD	Not a SR
Preincision adjunctive prophylaxis for cesarean deliveries a systematic review and meta-analysis.	Markwei MT; Babatunde I; Rathi N; Fan C; Prah MA; Joo J; Hackett L; Soper DE; Goje O	Previous version of SR
Best practice perioperative strategies and surgical techniques for preventing caesarean section surgical site infections: a systematic review of reviews and meta-analyses.	Martin EK; Beckmann MM; Barnsbee LN; Halton KA; Merollini K; Graves N	Not a SR
Abdominal surgical incisions for caesarean section.	Mathai M; Hofmeyr GJ	SR on surgical interventions
The association of skin incision placement during cesarean delivery with wound complications in obese women: a systematic review and meta-analysis.	Mccurdy RJ; Felder LA; Saccone G; Edwards RK; Thornburg LL; Marrs C; Conner SN; Strauss R; Berghella V	Specific population
Intravenous Magnesium Sulphate for Analgesia after Caesarean Section: A Systematic Review.	McKeown A; Seppi V; Hodgson R	Not a SR
Practices to Reduce Surgical Site Infections Among Women Undergoing Cesarean Section: A Review.	McKibben RA; Pitts SI; Suarez-Cuervo C; Perl TM; Bass EB	Not a SR
The Clinical Efficacy and Safety of Enhanced Recovery After Surgery for Cesarean Section: A Systematic Review and Meta-Analysis of Randomized Controlled Trials and Observational Studies.	Meng X; Chen K; Yang C; Li H; Wang X	Not a SR
Transversus abdominis plane block for analgesia after Cesarean	Mishriky BM; George RB; Habib AS	Previous version of SR

delivery: a systematic review and meta-analysis.		
Metoclopramide for nausea and vomiting prophylaxis during and after Caesarean delivery: a systematic review and meta-analysis.	Mishriky BM; Habib AS	Previous version of SR
Effectiveness of strategies for the management and/or prevention of hypothermia within the adult perioperative environment.	Moola S; Lockwood C	No outcomes/population of interest
The effects of an increase of central blood volume before spinal anesthesia for cesarean delivery: a qualitative systematic review.	Morgan PJ; Halpern SH; Tarshis J	Not a SR
Impact of changing gloves during cesarean section on postoperative infective complications: A systematic review and meta-analysis	Brenda F Narice, Joana R Almeida , Tom Farrell, Priya Madhuvrata	SR on surgical interventions
Codeine-acetaminophen versus nonsteroidal anti-inflammatory drugs in the treatment of post-abdominal surgery pain: a systematic review of randomized trials.	Nauta M; Landsmeer ML; Koren G	Specific population
Negative pressure wound therapy for surgical wounds healing by primary closure.	Norman G; Goh EL; Dumville JC; Shi C; Liu Z; Chiverton L; Stankiewicz M; Reid A	SR on surgical interventions
Negative pressure wound therapy for surgical wounds healing by primary closure.	Norman G; Goh EL; Dumville JC; Shi C; Liu Z; Chiverton L; Stankiewicz M; Reid A	Previous version of SR
Intracavity lavage and wound irrigation for prevention of surgical site infection	Howard Thom , Gill Norman, Nicky J Welton , Emma J Crosbie , Jane Blazeby , Jo C Dumville	
Tranexamic acid for preventing postpartum haemorrhage.	Novikova N; Hofmeyr GJ	Previous version of SR
Effects of perioperative oxygen concentration on oxidative stress in adult surgical patients: a systematic review.	Oldman AH; Martin DS; Feelisch M; Grocott MPW; Cumpstey AF	Specific population
Omission of the bladder flap at caesarean section reduces delivery time without increased morbidity: a meta-analysis of randomized controlled trials	Heidi A O'Neill , Grace Egan , Colin A Walsh, Amanda M Cotter, Stewart R Walsh	SR on surgical interventions
Carbetocin compared with oxytocin in non-elective Cesarean delivery: a systematic review, meta-analysis, and trial sequential analysis of randomized-controlled trials.	Onwochei DN; Owolabi A; Singh PM; Monks DT	Previous version of SR
Carbetocin reduces the need for additional uterotonics in elective caesarean delivery: a systematic review, meta-analysis and trial sequential analysis of randomised controlled trials.	Onwochei DN; Van Ross J; Singh PM; Salter A; Monks DT	SR on postpartum haemorrhage prevention
Retrospective analysis on the efficacy of corticosteroid prophylaxis prior to elective caesarean section to reduce neonatal respiratory complications at term of pregnancy: review of literature.	Paganelli S; Soncini E; Gargano G; Capodanno F; Vezzani C; La Sala GB	Not a SR
Interventions at caesarean section for reducing the risk of aspiration pneumonitis.	Paranjothy S; Griffiths JD; Broughton HK; Gyte GM; Brown HC; Thomas J	Previous version of SR
Interventions at caesarean section for reducing the risk of aspiration pneumonitis.	Paranjothy S; Griffiths JD; Broughton HK; Gyte GM; Brown HC; Thomas J	Previous version of SR

The Effect of Glycopyrrolate on the Incidence of Hypotension and Vasopressor Requirement During Spinal Anesthesia for Cesarean Delivery: A Meta-analysis.	Patel SD; Habib AS; Phillips S; Carvalho B; Sultan P	Previous version of SR
The impact of subcutaneous tissue suturing at caesarean section on wound complications: a meta-analysis	V Pergialiotis, A Prodromidou, D N Perrea, S K Doumouchtsis	SR on surgical interventions
Cephalad-caudad versus transverse blunt expansion of the low transverse hysterotomy during cesarean delivery decreases maternal morbidity: a meta-analysis	Vasilios Pergialiotis, Michalis Panagiotopoulos, Lito Vogiatzi , Ioannis Bellos , Panagiotis Antsaklis , Marianna Theodora, Ekaterini Ntomali , George Daskalakis	SR on surgical interventions
Spontaneous versus manual placental delivery during cesarean delivery: a systematic review and meta-analysis	Vasilios Pergialiotis, Michalis Panagiotopoulos, Lito Vogiatzi , Ioannis Bellos , Panagiotis Antsaklis , Marianna Theodora, Ekaterini Ntomali , George Daskalakis	SR on surgical interventions
Prevention of Surgical Site Infections: A Systematic Review of Cost Analyses in the Use of Prophylactic Antibiotics.	Purba AKR; Setiawan D; Bathoorn E; Postma MJ; Dik JH; Friedrich AW	Not a SR
Single- Versus Double-Layer Uterine Closure After Cesarean Section Delivery: A Systematic Review and Meta-Analysis	Kaif Qayum, Irfan Kar, Junaid Sofi, Hari Panneerselvam	SR on surgical interventions
Knotless Barbed versus Conventional Suture for Closure of the Uterine Incision at Cesarean Delivery: A Systematic Review and Meta-analysis	Hedi Benyamini Raischer , Manal Massalha, Rula Iskander , Ido Izhaki, Raed Salim	SR on surgical interventions
Postoperative Discharge Opioid Consumption, Leftover, and Disposal after Obstetric and Gynecologic Procedures: A Systematic Review.	Raina J; Costello C; Suarthana E; Tulandi T	No outcomes/population of interest
Changing gloves during cesarean section for prevention of postoperative infections: a systematic review and meta-analysis	Siwanon Rattanakanokchai, Nuntasiri Eamudomkarn, Nampet Jampathong , Bao-Yen Luong-Thanh, Chumnan Kietpeerakool	SR on surgical interventions
Autologous amniotic membrane: An accelerator of wound healing for prevention of surgical site infections following Cesarean delivery.	Rezazadeh D; Anvari Aliabad R; Norooznezhad AH	Not a SR
Fluid loading therapy to prevent spinal hypotension in women undergoing elective caesarean section: Network meta-analysis, trial sequential analysis and meta-regression.	Rijs K; Mercier FJ; Lucas DN; Rossaint R; Klimek M; Heesen M	Previous version of SR
Analgesic efficacy of the ultrasound-guided blockade of the transversus abdominis plane - a systematic review.	Ripollaos J; Mezquita SM; Abad A; Calvo J	Previous version of SR
Colloids versus crystalloids in the prevention of hypotension induced by spinal anesthesia in elective cesarean section. A systematic review and meta-analysis.	Ripollaos Melchor J; Espinosa Ã□; MartÃnez Hurtado E; Casans FrancÃ©s R; Navarro PÃ©rez R; Abad Gurumeta A; Calvo Vecino JM	Previous version of SR
Impact of single- vs double-layer closure on adverse outcomes and uterine scar defect: a systematic review and meta-analysis	Stéphanie Roberge, Suzanne Demers, Vincenzo Berghella , Nils Chaillet , Lynne Moore, Emmanuel Bujold	SR on surgical interventions
Povidone-iodine 1% is the most effective vaginal antiseptic for preventing post-cesarean	Roeckner JT; Sanchez-Ramos L; Mitta M; Kovacs A; Kaunitz AM	Previous version of SR

endometritis: aÂ systematic review and network meta-analysis.		
PROSPECT guideline for elective caesarean section: updated systematic review and procedure-specific postoperative pain management recommendations.	Roofthooft E; Joshi GP; Rawal N; Van de Velde M	Not a SR
Blunt versus sharp uterine incision expansion during low transverse cesarean delivery: a meta-analysis	Antonio F Saad, Mahbubur Rahman, Maged M Costantine , George R Saade	SR on surgical interventions
A Systematic Review Evaluating Neuraxial Morphine and Diamorphine-Associated Respiratory Depression After Cesarean Delivery.	Sharawi N; Carvalho B; Habib AS; Blake L; Mhyre JM; Sultan P	Not a SR
Evaluation of patient-reported outcome measures of functional recovery following caesarean section: a systematic review using the consensus-based standards for the selection of health measurement instruments (COSMIN) checklist.	Sharawi N; Klima L; Shah R; Blake L; Carvalho B; Sultan P	Not a SR
Chewing gum for postoperative recovery of gastrointestinal function.	Short V; Herbert G; Perry R; Atkinson C; Ness AR; Penfold C; Thomas S; Andersen HK; Lewis SJ	Previous version of SR
Use of hyperbaric versus isobaric bupivacaine for spinal anaesthesia for caesarean section.	Sia AT; Tan KH; Sng BL; Lim Y; Chan ES; Siddiqui FJ	Previous version of SR
Tranexamic acid for preventing postpartum blood loss after cesarean delivery: a systematic review and meta-analysis of randomized controlled trials.	Simonazzi G; Bisulli M; Saccone G; Moro E; Marshall A; Berghella V	Previous version of SR
The analgesic effectiveness of ilioinguinal-iliohypogastric block for caesarean delivery: A meta-analysis and trial sequential analysis.	Singh NP; Makkar JK; Bhatia N; Singh PM	Article missing
The analgesic efficacy of quadratus lumborum block in caesarean delivery: a meta-analysis and trial sequential analysis.	Singh NP; Makkar JK; Borle A; Monks D; Goudra BG; Zorrilla-Vaca A; Singh PM	Previous version of SR
Antibiotic prophylaxis for cesarean section.	Smaill F; Hofmeyr GJ	Previous version of SR
Antibiotic prophylaxis for cesarean section.	Smaill F; Hofmeyr GJ	Previous version of SR
Antibiotic prophylaxis versus no prophylaxis for preventing infection after cesarean section.	Smaill FM; Gyte GM	Previous version of SR
Hyperbaric vs. isobaric bupivacaine for spinal anaesthesia for elective caesarean section: a Cochrane systematic review.	Sng BL; Han NLR; Leong WL; Sultana R; Siddiqui FJ; Assam PN; Chan ES; Tan KH; Sia AT	SR on anaesthetic interventions
Subcuticular Suture Type at Cesarean Delivery and Infection Risk: A Systematic Review and Meta-Analysis	Olaoluwa Sobodu, Christopher M Nash, Jocelyn Stairs	SR on surgical interventions
Single dose perioperative intrathecal ketamine as an adjuvant to intrathecal bupivacaine: A systematic review and meta-analysis of adult human randomized controlled trials.	Sohnen S; Dowling O; Shore-Lesserson L	SR on anaesthetic interventions
Corticosteroids for preventing neonatal respiratory morbidity after elective caesarean section at term.	Sotiriadis A; Makrydimas G; Papatheodorou S; Ioannidis JP	Previous version of SR
Corticosteroids for preventing neonatal respiratory morbidity after elective caesarean section at term.	Sotiriadis A; Makrydimas G; Papatheodorou S; Ioannidis JP; McGoldrick E	Previous version of SR

Antenatal steroid administration in medically uncomplicated pregnancy beyond 37 weeks of gestation for the prevention of neonatal morbidities prior to elective caesarean section: a systematic review and meta-analysis of randomised controlled trials.	Srinivasjois R; Silva D	Previous version of SR
Uterine caesarean closure techniques affect ultrasound findings and maternal outcomes: a systematic review and meta-analysis.	Stegwee SI; Jordans I; van der Voet LF; van de Ven PM; Ket J; Lambalk CB; de Groot C; Hehenkamp W; Huirne J	No outcomes/population of interest
Systematic review of oxytocin dosing at caesarean section.	Stephens LC; Bruessel T	SR on postpartum haemorrhage prevention
Carbetocin for preventing postpartum haemorrhage.	Su LL; Chong YS; Samuel M	SR on postpartum haemorrhage prevention
Oxytocin agonists for preventing postpartum haemorrhage.	Su LL; Chong YS; Samuel M	SR on postpartum haemorrhage prevention
Enhanced recovery after caesarean delivery versus standard care studies: a systematic review of interventions and outcomes.	Sultan P; Sharawi N; Blake L; Carvalho B	Not a SR
Impact of enhanced recovery after cesarean delivery on maternal outcomes: A systematic review and meta-analysis.	Sultan P; Sharawi N; Blake L; Habib AS; Brookfield KF; Carvalho B	Not a SR
Prophylactic administration of cefazolin prior to skin incision versus antibiotics at cord clamping in preventing postcesarean infectious morbidity: a systematic review and meta-analysis of randomized controlled trials.	Sun J; Ding M; Liu J; Li Y; Sun X; Liu T; Chen Y	Previous version of SR
Effects of Abdominal Binders on Postoperative Pain and Functional Recovery: A Systematic Review and Meta-Analysis.	Sun X; Wei Q; Fu C; Zhang Q; Liang Z; Peng L; Chen L; He C	Specific population
Uterine exteriorization versus in-situ repair in Cesarean delivery: a systematic review and meta-analysis	Hon Sen Tan, Cameron R Taylor, Nadir Sharawi, Rehena Sultana , Karen D Barton , Ashraf S Habib	
Paucity of evidence for the effectiveness of prophylactic low-dose oxytocin protocols (<5 IU) compared with 5 IU in women undergoing elective caesarean section: A systematic review of randomised controlled trials.	Terblanche NCS; Picone DS; Otahal P; Sharman JE	Previous version of SR
Emerging concepts in antibiotic prophylaxis for cesarean delivery: a systematic review.	Tita ATN; Rouse DJ; Blackwell S; Saade GR; Spong CY; Andrews WW	Not a SR
Chlorhexidine-Alcohol Compared with Povidone-Iodine Preoperative Skin Antisepsis for Cesarean Delivery: A Systematic Review and Meta-Analysis.	Tolcher MC; Whitham MD; El-Nashar SA; Clark SL	Previous version of SR
Prophylaxis for venous thromboembolic disease in pregnancy and the early postnatal period.	Tooher R; Gates S; Dowswell T; Davis LJ	Previous version of SR
A systematic review and meta-analysis of the effect of prophylactic tranexamic acid treatment in major benign uterine surgery.	Topsoee MF; Settnes A; Ottesen B; Bergholt T	SR on postpartum haemorrhage prevention
Effects of Ondansetron on Attenuating Spinal Anesthesiaâ€“Induced Hypotension and Bradycardia in Obstetric and Nonobstetric Subjects: A Systematic Review and Meta-Analysis.	Tubog TD; Kane TD; Pugh MA	SR on anaesthetic interventions

Minimum Effective Dose (ED(50) and ED(95)) of Intrathecal Hyperbaric Bupivacaine for Cesarean Delivery: A Systematic Review.	Tubog TD; Ramsey VL; Filler L; Bramble RS	SR on anaesthetic interventions
Staples compared with subcuticular suture for skin closure after cesarean delivery: a systematic review and meta-analysis.	Tuuli MG; Rampersad RM; Carbone JF; Stamilio D; Macones GA; Odibo AO	Previous version of SR
The Health Impact of Surgical Techniques and Assistive Methods Used in Cesarean Deliveries: A Systemic Review.	Wang LH; Seow KM; Chen LR; Chen KH	Not a SR
The use of O-ring retractors at Caesarean section A systematic review and meta-analysis	G J Waring, S Shawer, K Hinshaw	SR on surgical interventions
Techniques for assisting difficult delivery at caesarean section	Weinstein	SR on surgical interventions
Local anaesthetics and regional anaesthesia versus conventional analgesia for preventing persistent postoperative pain in adults and children.	Weinstein EJ; Levene JL; Cohen MS; Andreae DA; Chao JY; Johnson M; Hall CB; Andreae MH	SR on anaesthetic interventions
Prophylactic Negative Pressure Wound Therapy in Closed Abdominal Incisions: A Meta-analysis of Randomised Controlled Trials.	Wells CI; Ratnayake CBB; Perrin J; Pandanaboyana S	Specific population
Chewing gum for intestinal function recovery after caesarean section: a systematic review and meta-analysis.	Wen Z; Shen M; Wu C; Ding J; Mei B	Previous version of SR
Comparison of Supraglottic Airway Devices With Endotracheal Intubation in Low-Risk Patients for Cesarean Delivery: Systematic Review and Meta-analysis.	White LD; Thang C; Hodsdon A; Melhuish TM; Barron FA; Godsall MG; Vlok R	SR on anaesthetic interventions
Absorbable staples for uterine incision at caesarean section.	Wilkinson C; Enkin MW	Previous version of SR
Lateral tilt for caesarean section.	Wilkinson C; Enkin MW	Previous version of SR
Manual removal of placenta at caesarean section.	Wilkinson C; Enkin MW	Previous version of SR
Uterine exteriorization versus intraperitoneal repair at caesarean section.	Wilkinson C; Enkin MW	Previous version of SR
Peritoneal non-closure at caesarean section.	Wilkinson CS; Enkin MW	Previous version of SR
Guidelines for Antenatal and Preoperative care inÂ Cesarean Delivery: Enhanced Recovery After SurgeryÂ Society Recommendations (PartÂ 1).	Wilson RD; Caughey AB; Wood SL; Macones GA; Wrench IJ; Huang J; Norman M; Pettersson K; Fawcett WJ; Shalabi MM; Metcalfe A; Gramlich L; Nelson G	Not a SR
Advanced dressings for the prevention of surgical site infection in women post-caesarean section: A systematic review and meta-analysis	Samodani Wijetunge , Ruby Hill , R Katie Morris 3 , Victoria Hodgetts Morton	SR on surgical interventions
Phenylephrine vs ephedrine in cesarean delivery under spinal anesthesia: A systematic literature review and meta-analysis.	Xu C; Liu S; Huang Y; Guo X; Xiao H; Qi D	Previous version of SR
Blunt vs. sharp uterine expansion at lower segment cesarean section delivery: a systematic review with metaanalysis.	Xu LL; Chau AM; Zuschmann A	Previous version of SR
The efficacy and safety of pharmacologic thromboprophylaxis following caesarean section: A systematic review and meta-analysis.	Yang R; Zhao X; Yang Y; Huang X; Li H; Su L	Previous version of SR

Prophylactic negative-pressure wound therapy after cesarean is associated with reduced risk of surgical site infection: aÂ systematic review and meta-analysis.	Yu L; Kronen RJ; Simon LE; Stoll CRT; Colditz GA; Tuuli MG	Specific population
Uterine exteriorization compared with in situ repair for Cesarean delivery: a systematic review and meta-analysis.	Zaphiratos V; George RB; Boyd JC; Habib AS	Previous version of SR
Timing of Antibiotic Prophylaxis in Elective Caesarean Delivery: A Multi-Center Randomized Controlled Trial and Meta-Analysis.	Zhang C; Zhang L; Liu X; Zeng Z; Li L; Liu G; Jiang H	Not a SR
Dexmedetomidine as a neuraxial adjuvant for prevention of perioperative shivering: Meta-analysis of randomized controlled trials.	Zhang J; Zhang X; Wang H; Zhou H; Tian T; Wu A	No outcomes/population of interest
Foetal responses to dexmedetomidine in parturients undergoing caesarean section: a systematic review and meta-analysis.	Zhang J; Zhou H; Sheng K; Tian T; Wu A	No outcomes/population of interest
Effects of gum chewing on postoperative bowel motility after caesarean section: a meta-analysis of randomised controlled trials.	Zhu YP; Wang WJ; Zhang SL; Dai B; Ye DW	Previous version of SR
Sutures versus clips for skin closure following caesarean section: a systematic review, meta-analysis and trial sequential analysis of randomised controlled trials	Shafquat Zaman , Ali Yasen Y Mohamedahmed, Elizabeth Peterknecht , Reza Md Zakaria , Saeed Y Y Mohamedahmed, Shahab Hajibandeh , Shahin Hajibandeh	SR on surgical interventions
Comparison of the Joel-Cohen-based technique and the transverse Pfannenstiel for caesarean section for safety and effectiveness: A systematic review and meta-analysis.	Olyaeemanesh A; Bavandpour E; Mobinizadeh M; Ashrafinia M; Bavandpour M; Nouhi M	Unclear data

Supplementary Material 4: Characteristic of included studies

Review ID	Assessed as up to date	N° of trials	N° of participants	Includes LMIC? (Y/N)	Type of C-Section (prelabour /intrapartum /both)	CR / NCR	AMSTAR Score Online	Title
Abdel-Aleem 2014	2013	5	1090	Y	Both	CR	HIGH	Indwelling bladder catheterization as part of intraoperative and postoperative care for caesarean section
Abd-ElGawad 2020	2019	6	702	Y	Both	NCR	HIGH	The effectiveness of the abdominal binder in relieving pain after cesarean delivery: A systematic review and meta-analysis of randomized controlled trials
Abuzaid 2024	2024	7	1006	Y	Unclear	NCR	CRIT LOW	Effectiveness of preoperative multimedia educational sessions on the levels of anxiety and satisfaction among women undergoing cesarean: a systematic review and meta-analysis
Bolling 2018	2017	18	Unclear	Y	Both	NCR	CRIT LOW	Prophylactic antibiotics before cord clamping in cesarean delivery: a systematic review
Chaarani 2024	2024	7	4267	Y	Both	NCR	CRIT LOW	Early hospital discharge after cesarean delivery: a systematic review and meta-analysis of randomized controlled trials
Chen 2024	2024	8	2572	Y	Both	NCR	CRIT LOW	Early oral feeding within two hours for parturients compared with delayed oral feeding after cesarean section: systematic review and meta-analysis
Guo 2015	2015	20	4584	Unclear	Unclear	NCR	LOW	Early versus delayed oral feeding for patients after cesarean.
Haas 2020	2020	21	7038	Y	Both	CR	HIGH	Vaginal preparation with antiseptic solution before cesarean section for preventing postoperative infections
Hsu 2013	2013	17	2966	Y	Unclear	NCR	CRIT LOW	Early Oral Intake and Gastrointestinal Function After Cesarean Delivery
Jones 2021	2021	17	9409	Y	Both	CR	HIGH	Early postnatal discharge from hospital for healthy mothers and term infants
Kim 2021	2020	7	1911	Unclear	Unclear	NCR	LOW	Effects of Early Oral Feeding versus Delayed Oral Feeding on Bowel Function, Gastrointestinal Complications and Surgical Recovery after Cesarean Section under Regional Anesthesia: Systematic Review and Meta-Analysis
Liu 2023	2022	23	10026	Y	Unclear	NCR	LOW	Different methods of vaginal preparation before cesarean delivery to prevent postoperative infection: a systematic review and network meta-analysis
Mackeen 2014	2014	10	5041	Y	Both	CR	HIGH	Timing of intravenous prophylactic antibiotics for preventing postpartum infectious morbidity in women undergoing cesarean delivery.
Mangesi 2002	2002	6	809	Unclear	Both	CR	LOW	Early compared with delayed oral fluids and food after caesarean section.
Markewi 2021	2021	4	2613	Y	Non elective	NCR	HIGH	Preincision adjunctive prophylaxis for cesarean deliveries a systematic review and meta-analysis
Menshawi 2018	2017	3	609	Unclear	Elective	NCR	LOW	Early compared with delayed oral fluids and food after caesarean section.
Middleton 2021	2021	29	3839	N	Both	CR	HIGH	Venous thromboembolism prophylaxis for women at risk during pregnancy and the early postnatal period.
Moore 2016	2016	8	703	Y	Both/Elective	CR	HIGH	Early skin-to-skin contact for mothers and their healthy newborn infants

Motaze 2013	2013	1	36	N	Elective	CR	HIGH	Prostaglandins before caesarean section for preventing neonatal respiratory distress.
Nabhan 2016	2016	10	1354	Y	Both	CR	HIGH	Routes of administration of antibiotic prophylaxis for preventing infection after caesarean section.
Pereira Gomes Morais 2016	2016	17	3149	Y	Both	CR	HIGH	Chewing gum for enhancing early recovery of bowel function a er caesarean section
Pinto Lopes 2016	2015	16	2695	Y	Both	NCR	LOW	Single dose versus multiple dose of antibiotic prophylaxis in caesarean section: a systematic review and meta-analysis.
Smaill 2014	2014	95	over 15000	Y	Both	CR	HIGH	Antibiotic prophylaxis versus no prophylaxis for preventing infection after cesarean section.
Sotiriadis 2021	2022	1	942	N	Elective	CR	HIGH	Antenatal corticosteroids prior to planned caesarean at term for improving neonatal outcomes.
Wetterslev 2015	2015	28	1719	Y	Unclear	CR	HIGH	The effects of high perioperative inspiratory oxygen fraction for adult surgical patients
Williams 2021	2021	33	8073	Y	Both	CR	HIGH	Different classes of antibiotics given to women routinely for preventing infection at caesarean section.
Yang 2022	2022	3	3834	Unclear	Both	NCR	HIGH	Efficacy of adding azithromycin to antibiotic prophylaxis in caesarean delivery: a meta-analysis and systematic review
Yonemoto 2021	2021	16	12080	Y	Unclear	CR	HIGH	Schedules for home visits in the early postpartum period
Zeng 2023	2023	16	8027	Y	Both	NCR	LOW	Timing of Intravenous Prophylactic Antibiotic Agents for Cesarean Delivery: A Systematic Review and Meta-Analysis of Randomized Controlled Trials

NR: Not reported.

Supplementary material 5: AMSTAR Score of included systematic reviews

Systematic review	Q1: PICO included	Q2: Review methods	Q3: Study design	Q4: Literature search	Q5: Study selection	Q6: Data extraction	Q7: Excluded studies	Q8: Included studies	Q9: ROB assessment	Q10: Source of funding	Q11: Results	Q12: ROB assessment	Q13: ROB individual studies	Q14: Heterogeneity	Q15: Publication bias	Q16: COI	Amstar score
Abdel-aleem 2014	Y	Y	Y	Y	Y	Y	Y	Y	Y	Y	Y	Y	Y	Y	Y	Y	HIGH
Abd-elgawad 2020	Y	PY	Y	Y	Y	Y	Y	Y	Y	Y	Y	Y	Y	Y	Y	Y	HIGH
Abuzaid 2024	Y	Y	Y	Y	Y	Y	N	Y	Y	N	Y	N	N	Y	N	Y	CRIT LOW
Bolling 2018	Y	Y	Y	Y	Y	N	N	Y	N	Y	Y	Y	Y	Y	N	Y	CRIT LOW
Chaarani 2024	Y	Y	Y	Y	Y	Y	PY	Y	Y	N	Y	Y	Y	Y	Y	Y	HIGH
Chen 2024	Y	Y	Y	Y	N	Y	N	Y	Y	N	Y	N	Y	Y	N	Y	CRIT LOW
Guo 2015	Y	N	Y	Y	Y	Y	Y	Y	Y	Y	Y	Y	Y	Y	Y	Y	LOW
Haas 2020	Y	Y	Y	Y	Y	Y	Y	Y	Y	Y	Y	Y	Y	Y	Y	Y	HIGH
Hsu 2013	Y	N	N	Y	Y	Y	N	Y	N	Y	Y	N	N	Y	N	Y	CRIT LOW
Jones 2021	Y	Y	Y	Y	Y	Y	Y	Y	Y	Y	Y	Y	Y	Y	Y	Y	HIGH
Kim 2021	Y	Y	Y	Y	N	N	Y	PY	Y	Y	Y	Y	Y	Y	Y	Y	LOW
Liu 2023	Y	Y	Y	Y	Y	Y	N	Y	Y	N	Y	Y	Y	Y	Y	Y	LOW
Mackeen 2014	Y	Y	Y	Y	Y	Y	Y	Y	Y	N	Y	Y	Y	Y	Y	Y	HIGH
Mangesi 2002	Y	Y	Y	PY	Y	Y	Y	PY	Y	N	Y	Y	Y	Y	N	Y	LOW
Markewi 2021	Y	Y	Y	Y	Y	Y	Y	Y	Y	Y	Y	Y	Y	Y	Y	Y	HIGH
Menshawi 2018	Y	N	Y	Y	Y	Y	Y	Y	Y	Y	Y	Y	Y	Y	Y	Y	LOW
Middleton 2021	Y	Y	Y	Y	Y	Y	Y	Y	Y	Y	Y	Y	Y	Y	Y	Y	HIGH
Moore 2016	Y	Y	Y	Y	Y	Y	Y	Y	Y	Y	Y	Y	Y	Y	Y	Y	HIGH
Motaze 2013	Y	Y	Y	Y	Y	Y	Y	Y	Y	Y	Y	Y	Y	Y	Y	Y	HIGH

Nabhan 2016	Y	Y	Y	Y	Y	Y	Y	Y	Y	Y	Y	Y	Y	Y	Y	Y	HIGH
Pereira gomes morais 2016	Y	Y	Y	Y	Y	Y	Y	Y	Y	Y	Y	Y	Y	Y	Y	Y	HIGH
Pinto lopes 2016	Y	Y	Y	Y	Y	Y	N	Y	Y	Y	Y	Y	Y	Y	Y	Y	LOW
Smaill 2014	Y	Y	Y	Y	Y	Y	Y	Y	Y	Y	Y	Y	Y	Y	Y	Y	HIGH
Sotiriadis 2021	Y	Y	Y	Y	Y	Y	Y	Y	Y	Y	Y	Y	Y	Y	Y	Y	HIGH
Wetterslev 2015	Y	Y	Y	Y	Y	Y	Y	Y	Y	Y	Y	Y	Y	Y	Y	Y	HIGH
Williams 2021	Y	Y	Y	Y	Y	Y	Y	Y	Y	Y	Y	Y	Y	Y	Y	Y	HIGH
Yang 2022	Y	Y	Y	Y	Y	Y	Y	Y	Y	Y	Y	Y	Y	Y	Y	Y	HIGH
Yonemoto 2021	Y	Y	Y	Y	Y	Y	Y	Y	Y	Y	Y	Y	Y	Y	Y	Y	HIGH
Zeng 2023	Y	Y	Y	Y	Y	Y	N	Y	Y	N	Y	Y	Y	Y	Y	Y	LOW

Q: Question; Y: Yes; N: No; PY: Partial yes

Supplementary material 6. Summary of findings

**Procedure-outcome comparisons**				
	**Procedure versus procedure**	**Procedure versus NT*/placebo**	Total	%
Clear evidence of benefit	37	60	97	18.9
Possible benefit	25	23	48	9.3
Clear evidence no difference	3	0	3	0.6
Possible no difference	4	2	6	1.2
Clear evidence of harm	1	3	4	0.8
Possible harm	2	2	4	0.8
Insufficient evidence	199	151	350	68.4
Total	271	241	512	100

*N/T: no treatment

Supplementary material 7. Procedure outcomes pair without SRs, with insufficient evidence and not reported outcomes

Intervention	Intervention arm	Control arm	Outcomes reported by SRs
**Bladder evacuation**	Indwelling bladder catheter	No catheter	Urinary tract infection – as defined by trialists
			Postpartum haemorrhage
			Length of hospital stay (days) (d)
		In-out urethral catheter	Urine retention
			Intensive Care Unit admission– as defined by trialists
			Time to patient ambulation (hours) (h)
			Length of hospital stay (h)
**Urinary catherter removal**	Immendiate Urinary catherter removal	Delayed urinary catheter removal	Urinary retention
			Urgency
			Post-operative oral rehydration
			Length of hospital stay (d)
**Preoperative vaginal preparation**	Antiseptic solution – All women	No preparation or saline preparation	Composite wound complication
	Antiseptic solution - Women in labor	No preparation or saline preparation	Composite wound complication
			Fever
			Surgical site infection (SSI)
	Antiseptic solution - Women not in labor	No preparation or saline preparation	Composite wound complication
			Composite wound complication or endometritis
			Endometritis
			Fever
			Surgical site infection (SSI)
	Chlorhexidine-based solution	No preparation or saline preparation	Postoperative fever
			Surgical site infection (SSI)
	Guanidine base	No preparation or saline preparation	Fever
			Surgical site infection (SSI)
**Prophylactic antibiotics - Type of antibiotic**	Antibiotic prophylaxis – All types	No antibiotics prophylaxis	Maternal adverse effects
	Natural penicillins	No antibiotics prophylaxis	Surgical site infection (SSI)
	Extended spectrum penicillins	No antibiotics prophylaxis	Maternal serious infectious complications
			Maternal adverse effects
	First generation cephalosporins	No antibiotics prophylaxis	Maternal adverse effects
			Maternal days in hospital (d)
	Second generation cephalosporins	No antibiotics prophylaxis	Maternal serious infectious complications
			Maternal adverse effects
	Third generation cephalosporins	No antibiotics prophylaxis	Maternal serious infectious complications
			Maternal urinary tract infection
			Maternal adverse effects
	Cefamycins	No antibiotics prophylaxis	Maternal serious infectious complications
			Maternal urinary tract infection
			Maternal adverse effects

	Beta-lactamase inhibitor combinations	No antibiotics prophylaxis	Endometritis
			Maternal adverse effects
			Maternal days in hospital
			Endometritis
	Aminopenicillins	No antibiotics prophylaxis	Maternal serious infectious complications
	Monobactams	No antibiotics prophylaxis	Maternal febrile morbidity/fever
			Endometritis
			Surgical site infection (SSI)
			Maternal urinary tract infection
			Maternal serious infectious complications
	Lincosamides	No antibiotics prophylaxis	Maternal febrile morbidity/fever
			Surgical site infection (SSI)
	Nitroimidazoles	No antibiotics prophylaxis	Maternal serious infectious complications
			Maternal urinary tract infection
	Trimethoprim-sulfamethoxazole	No antibiotics prophylaxis	Endometritis
	Aminoglycoside-containing combination	No antibiotics prophylaxis	Maternal serious infectious complications
			Maternal urinary tract infection
			Maternal adverse effects
	Other regimens	No antibiotics prophylaxis	Maternal febrile morbidity/ fever
			Surgical site infection (SSI)
			Endometritis
			Maternal urinary tract infection
	Other antibiotics combination	No antibiotics prophylaxis	Surgical site infection (SSI)
			Maternal urinary tract infection
	Any antibiotics - elective CS	No antibiotics prophylaxis	Maternal serious infectious complications
			Maternal urinary tract infection
			Maternal adverse effects
			Maternal adverse effects
	Any antibiotics – both elective and non-elective CS	No antibiotics prophylaxis	Maternal adverse effects
	Any antibiotics - timing of administration not defined	No antibiotics prophylaxis	Maternal serious infectious complications
	Antibiotics before cord clamping	No antibiotics prophylaxis	Maternal adverse effects
	Any antibiotics after cord clamping	No antibiotics prophylaxis	Maternal adverse effects
	Any antibiotics - timing of administration not defined	No antibiotics prophylaxis	Maternal adverse effects
**Prophylactic antibiotics (Type)**	Antistaphylococcal cephalosporins C1 and C2 (1st and 2nd generation)	Broad spectrum penicillins plus betalactamase inhibitors P2+	Maternal sepsis
			Endometritis
			Maternal fever (febrile morbidity)
			Surgical site infection (SSI)
			Maternal urinary tract infection
			Maternal composite adverse effects
			Maternal allergic reactions
			Maternal skin rash

			Endometritis
			Maternal fever (febrile morbidity)
			Surgical site infection (SSI)
			Maternal urinary tract infection
			Maternal composite adverse effects
			Maternal allergic reactions
	Minimally antistaphylococcal cephalosporins C3 (3rd generation)	Non-antistaphylococcal penicillins P1 and P2 (natural and broad spectrum)	Maternal sepsis
			Maternal fever (febrile morbidity)
			Surgical site infection (SSI)
			Maternal urinary tract infection
			Maternal composite serious infectious Complication
			Maternal composite adverse effects
			Maternal allergic reactions
			Maternal nausea/vomiting
			Maternal diarrhea/skin rash
	Minimally antistaphylococcal cephalosporins C3 (3rd generation)	Broad spectrum penicillins plus betalactamase inhibitors P2+	Endometritis
			Maternal fever (febrile morbidity)
			Surgical site infection (SSI)
			Urinary tract infection
			Maternal composite serious infectious complication
			Maternal allergic reactions
			Maternal nausea/vomiting/diarrhea/skin rush
			Maternal length of hospital stay (d)
	Cephalosporins C3 (3rd generation)	Penicillins P2 and P3 (broad spectrum and antistaphyloccal)	Endometritis
			Maternal fever (febrile morbidity)
			Surgical site infection (SSI)
			Maternal vomiting
			Maternal skin rash
	Fluoroquinolones F	Broad spectrum penicillin plus betalactamase inhibitors P2+	Maternal sepsis
			Endometritis
			Surgical site infection (SSI)
			Maternal urinary tract infection
			Maternal sepsis
	Carbapenems Ca	Cephalosporins C3 (3rd generation)	Endometritis
			Maternal fever (febrile morbidity)
			Surgical site infection (SSI)
			Maternal urinary tract infection
	Macrolides M	Cephalosporins C1 (1st generation)	Maternal fever (febrile morbidity)
	Broad spectrum penicillin P2 plus antistaphylococcal penicillin P3 plus aminoglycoside A plus nitroimidazole N	Cephalosporin C3 (3rd generation)	Endometritis
			Maternal fever (febrile morbidity)
			Surgical site infection (SSI)
			Maternal urinary tract infection

			Maternal length of hospital stay (h)
	Lincosamide L plus aminoglycoside A	Natural penicillin P1	Endometritis
			Surgical site infection (SSI)
	Cephalosporin C1 (1st generation) plus nitroimidazole N	Broad spectrum penicillin P2	Endometritis
			Surgical site infection (SSI)
			Maternal fever (febrile morbidity)
		Broad spectrum penicillin plus betalactamase inhibitors P2+	Endometritis
			Surgical site infection (SSI)
			Maternal fever (febrile morbidity)
			Maternal urinary tract infection
	Cephalosporin C3 (3rd generation) plus nitroimidazole N	Natural penicillin P1 plus broad spectrum penicillin P2 plus nitroimidazole N plus amphenicol Am	Maternal sepsis
			Surgical site infection (SSI)
			Maternal fever (febrile morbidity)
	Aminoglycoside A plus nitroimidazole N	Natural penicillin P1 plus nitroimidazole N plus macrolide M	Maternal sepsis
			Surgical site infection (SSI)
	Antistaphylococcal cephalosporin C1 and C2 (1st and 2nd generation) plus nitroimidazole N	Non-antistaphylococcal penicillins P1 and P2 (natural and broad spectrum) plus nitroimidazole N	Surgical site infection (SSI)
			Maternal urinary tract infection
			Maternal length of hospital stay (h)
			Endometritis
			Maternal fever (febrile morbidity)
			Maternal composite adverse effects
			Costs
	Aminoglycoside A plus nitroimidazole N	Natural penicillin P1 plus nitroimidazole N plus macrolide M	Maternal urinary tract infection
			Maternal length of hospital stay
	Cefazolin with adjunctive prophylaxis	Cefazolin alone	Febrile morbidity
**Prophylactic antibiotics (route)**	Irrigation/lavage: Cephalosporins C2 (2nd generation)	Irrigation/lavage: Penicillins: Penicillins P2 (broad spectrum)	Endometritis
	Irrigation/lavage: cephalosporins	Irrigation/lavage: Penicillins	Maternal fever (febrile morbidity)
			Maternal endometritis
	Irrigation/lavage: cephalosporins	Irrigation/lavage: Penicillins	Surgical site infection (SSI)
	Intravenous (IV)	Irrigation	Endometritis
			Surgical site infection (SSI)
			Postpartum febrile morbidity
			Urinary tract infection
			Serious infectious complication
			Adverse events (maternal)
			Maternal length of hospital stay (d)
	Intravenous (IV) – low risk of bias	Irrigation – low risk of bias	Endometritis
			Surgical site infection (SSI)
**Prophylactic antibiotics (dose)**	Multiple doses		Postpartum infectious morbidity
			Endometritis
			Surgical site infection (SSI)

			Urinary tract infection
	Mezlocilin - Multiple doses	Mezlocilin - Single dose	Postpartum infectious morbidity
	Piperacilin - Multiple doses	Piperacilin - Single dose	Postpartum infectious morbidity
	Cefoxitin - Multiple doses	Cefoxitin - Single dose	Postpartum infectious morbidity
	Cefotaxime - Multiple doses	Cefotaxime - Single dose	Postpartum infectious morbidity
	Cefazolin - Multiple doses	Cefazolin - Single dose	Postpartum infectious morbidity
	Cefazolin + metronidazole - Multiple doses Cefazolin + metronidazole	Cefazolin + metronidazole - Single dose	Postpartum infectious morbidity
	Ampicillin + metronidazole - Multiple doses	Ampicillin + metronidazole - Single dose	Postpartum infectious morbidity
	Gentamicin + metronidazole - Multiple doses	Gentamicin + metronidazole - Single dose	Postpartum infectious morbidity
	Penicilins - Multiple doses	Penicilins -Single dose	Postpartum infectious morbidity
	Cephalosporines - Multiple doses	Cephalosporines - Single dose	Postpartum infectious morbidity
	Aminoglycosides - Multiple doses	Aminoglycosides - Single dose	Postpartum infectious morbidity
	Multiple doses -Single antibiotic	Single dose - Single antibiotic	Postpartum infectious morbidity
	Multiple doses -Combination of antibiotics	Single dose - Combination of antibiotics	Postpartum infectious morbidity
	Multiple doses - Low resource setting	Single dose - Low resource setting	Postpartum infectious morbidity
	Multiple doses - High resource setting	Single dose - High resource setting	Postpartum infectious morbidity
	Multiple doses - Emergency CS	Single dose - Emergency CS	Postpartum infectious morbidity
	Multiple doses - Elective CS	Single dose - Elective CS	Postpartum infectious morbidity
	Multiple doses - Both types of CS	Single dose - Both types of CS	Postpartum infectious morbidity
**Prophylactic antibiotics (timining)**	Antibiotics before cord clamping	After cord clamping	Urinary tract infection/cystitis/pyelonephritis
			Respiratory tract infections
			Fever
			Adverse events attributed to the antibiotic.
			Sepsis
			Suspected sepsis
			Infection with an antibiotic resistant bacterium.
			Pelvic abscess
			Febrile illness
			Neonatal sepsis
			Infection (other)
			Neonatal antibiotic treatment
			Neonatal fever
			Intensive care unit admission
			Length of intensive care unit stay
**Oxigen inspiration fraction**	High inspiration oxygen fraction: 60% to 90% oxygen perioperatively	Lower inspiration oxygen fraction: oxygen 30% to 40% oxygen perioperatively	Maternal mortality (overall)
			Surgical site infection (any type of surgeries)
			Surgical site infection (CS)
			Respiratory insufficiency
			Serious adverse events

			Length of stay after surgery
**Corticosteroids for preventing neonatal respiratory distress syndrome**	Antenatal corticosteroids (betamethasone) at term	Usual care at term	Respiratory distress syndrome
			Transient tachypnoea
			Need for mechanical ventilation
			Maternal development of postpartum infection
			Neonatal infectious morbidity
			Perinatal deaths
			Readmission for respiratory problems after initial discharge
			Cognitive impairment
			Emotional and behavioural problems measured with strengths and difficulties questionnaire
			Adverse maternal effects of therapy
	Antenatal corticosteroids (betamethasone) (Birth before 38 + 0 weeks)	Usual care (Birth before 38 + 0 weeks)	Respiratory distress syndrome
			Transient tachypnoea
			Admission to neonatal special care (all levels) for respiratory morbidity
			Admission to neonatal special care (all levels) for any indication
	Antenatal corticosteroids (betamethasone) (Birth 38 + 0 to 38 + 6 weeks)	Usual care (Birth 38 + 0 to 38 + 6 weeks)	Respiratory distress syndrome
			Admission to neonatal special care (all levels) for respiratory morbidity
			Admission to neonatal special care (all levels) for any indication
	Antenatal corticosteroids (betamethasone) (Birth at or after 39 + 0 weeks)	Usual care (Birth at or after 39 + 0 weeks)	Respiratory distress syndrome
			Transient tachypnoea
			Admission to neonatal special care (all levels) for respiratory morbidity
			Admission to neonatal special care (all levels) for any indication
**Skin to skin contact**	Immediate or Early skin-to-skin	Standard contact for healthy infants after cesarean birth	Breastfeeding 1 month to 4 months post birth
			Exclusive breastfeeding at hospital discharge to 1 month post birth
			Exclusive breastfeeding 6 weeks to 6 months post birth
			Success of the first breastfeeding (IBFAT score)
			Respiratory rate 75 minutes - 2 hours post birth
			Maternal pain 4 hours post-cesarean birth
			Maternal state anxiety 8 hours to 3 days post birth
**Time to fluids and food**	Early oral intake	Delay oral intake	Ileus symptom
			Diarrohea
			Passage of stool (h)
			Nausea
			Vomiting
			Abdominal distention
			Ileus symptom
			Fever
			Wound infection
			Urinary tract infection
			Readmission

			Initiation of ambulation (h)
			Score for patient satisfaction
			Analgesic doses postoperatively
			Length of hospital stay
	Early oral intake - Regional Analgesia	Delay oral intake - Regional Analgesia	Abdominal distension
			Analgesic doses postoperatively
			Time to passing flatus (h)
			Time to bowel movement (h)
			Score for patient satisfaction
	Early oral intake - Anaesthesia mixed or not defined.	Delay oral intake - Anaesthesia mixed or not defined	Postoperative nausea
			Abdominal distension
			Analgesic doses postoperatively
	Chewing gum	Control	Time to first passage of flatus (h)
	Chewing gum - More than 1 hour/day	Control	Time to first passage of flatus (h)
	Chewing gum - Up to 1 hour/day	Control	Time to first passage of flatus (h)
	Chewing gum - No information on time chewing per day	Control	Time to first passage of flatus (h)
	Chewing gum	Early feeding	Time to first passage of flatus (h)
	Chewing gum	Conventional feeding	Time to first passage of flatus (h)
	Chewing gum – Elective CS	Control	Time to first passage of flatus (h
	Chewing gum – Type of CS not defined	Control	Time to first passage of flatus (h)
	Chewing gum - Imediately after CS	Control	Time to first passage of flatus (h)
	Chewing gum - 5 hours after CD	Control	Time to first passage of flatus (h)
	Chewing gum - 6 hours or more after CS		Time to first passage of flatus (h)
	Chewing gum - Elective CS	Control	Proportion of participants with ileus
	Chewing gum	Control	Proportion of participants with ileus immediately after CS
	Chewing gum	Control	Proportion of participants with ileus 2 to 5 hours after CS
	Chewing gum	Control	Proportion of participants with ileus 6 hours or more after CS
	Chewing gum	Control	Time to passage of faeces (h)
	Chewing gum	Control	Need for additional analgesics/antiemetics.
**Hospital discharge**	Early discharge	Standard discharge	Infants readmitted for neonatal morbidity within 7 days
			Infants readmitted for neonatal morbidity within 28 days
			Infants readmitted for neonatal morbidity within 28 days: subgroups < 24h vs >24h (Early discharge more than 24 hours)
			Infants readmitted for neonatal morbidity within 28 days: subgroups < 24h and >24h
			Women readmitted within six weeks
			Women readmitted within six weeks: Vaginal and CS
			Women readmitted within six weeks: (Early discharge less than 24-28hs, standard discharge >=48hs)
			Women probably depressed within six months: CS
			Women probably depressed within six months: subgroups < 24 h

			Women breastfeeding (exclusively or partially) at six weeks postpartum: subgroups < 24hr vs > 24 hs
			Women breastfeeding (partially or exclusively) at six months postpartum (Early discharge < 24 hours)
			Women breastfeeding (partially or exclusively) at six months postpartum (Early discharge > 24 hours)
			Women breastfeeding (partially or exclusively) at six months postpartum (Early discharge range < 24 hours and > 24 hours)
			Women breastfeeding (partially or exclusively) at six months postpartum
			Infant mortality within 28 days
			Infant mortality within one year
			Number of contacts with healthcare professionals regarding infant health issues within four weeks of birth
			Number of contacts with healthcare professionals regarding maternal health issues within six weeks of birth
			Women reporting infant feeding problems
			Women satisfied with postnatal care - dichotomous data
			Satisfaction with postnatal care - continuous data
			Women who perceive their length of hospital stay as too short
			Women perceive their length of hospital stay as too long
**Post-discharge controls**	Schedules involving more visits	Fewer home visits	Neonatal mortality
			Severe maternal morbidity
			Dyspareunia
	Schedules involving more visits	Fewer home visits- More vs fewer visits (both groups had more than 4 visits)	Maternal mortality within 42 days
			Exclusive breastfeeding (last assessment up to 6 weeks)
			Exclusive breastfeeding (last assessment up to 6 months)
	Schedules involving more visits	Fewer home visits (4 or more visits vs less than 4)	Neonatal mortality
			Severe maternal morbidity
			Infant respiratory tract infection within 42 days
	Schedules involving more visits	Fewer home visits (Home visits vs no home visits)	Neonatal mortality
			Post Partum Haemorrhage
			Abdominal pain up to 42 days
			Urinary tract complications up to 42
			Maternal fever up to 42 days postpartum
			Infant respiratory tract infection within 42 days
			Exclusive breastfeeding (last assessment up to 6 weeks)
	Schedules involving home visits	No home visits	Infant jaundice
	Flexible schedule	Routine visits	Neonatal mortality
	Breastfeeding promotion	Routine visits	Exclusive breastfeeding (last assessment up to 6 months)
	Home visit	Facility postnatal care	Severe maternal morbidity (emergency health care visits)
			Severe maternal morbidity (hospital readmissions)
			Mean satisfaction score with postnatal care
			Non prespecified - Infant emergency health care visits
**Education/ information**	Information video	Control	Postoperative satisfaction
**Thromboprophylaxis**	Heparin (LMWH or UFH)	No treatment/placebo	Maternal death

			Symptomatic thromboembolic events
			Symptomatic pulmonary embolism
			Symptomatic deep vein thrombosis
			Blood transfusion
			Major bleeding
			Major bruising
			Bleeding complications
			Bleeding/bruising reported at discharge
			Blood loss < 500 mL
			Blood loss 500-1000 mL
			Blood loss 1000-1500 mL
			Blood loss 1500-2000 mL
			Major wound disruption
			Surgical site infection (SSI)
			Adverse effects sufficient to stop treatment
			Adverse effects not sufficient to stop treatment
	Hydroxyethyl starch (HES)	Heparin (UFH)	Asymptomatic thromboembolic events
			Blood transfusion
			Bleeding episodes
			Serious wound complications
	Heparin (LMWH)	Heparin (UFH)	Symptomatic thromboembolic events
			Symptomatic pulmonary embolism
			Symptomatic deep vein thrombosis
			Bleeding episodes"haemorrhagic event"
			Major bleeding
			Post surgical haemorrhage
			Adverse effects not sufficient to stop treatment
			Thrombocytopenia

Not pre-specifed comparison-outcome pairs with insufficient evidence			
**Intervention**	**Intervention arm**	**Control arm**	**Outcomes reported by SRs**
Prostaglandins	Prostaglandin E2 gel	Placebo	Respiratory distress
Oxygen fraction inspiration	Lower inspiration oxygen fraction	Lower inspiration oxygen fraction	Maternal mortality
			Surgical site infection
			Respiratory insufficiency
			Serious adverse events
			Length of stay after surgery (d)

Pre-specified interventions with no systematic review
Preoperative evaluation
Preoperative Cardiovascular evaluation

Preoperative Laboratory tests
Preoperative Washing/ Bathing
Preoperative Shaving
Preoperative Fasting
Preoperative IV fluids
Time to resume physical activity
Time to resume sexual activity
Wound care
Time to stich removal
Use of protocols for caesarean section
Companionship during CS

Pre-specified outcomes with IE in the systematic reviews
Febrile morbidity: sepsis
Haemorrhagic morbidity: blood transfusion, need for additional uterotonic
Pain: need for additional analgesics
Long-term surgical complications: dyspareunia
Severe morbidity: thrombosis, embolism
Maternal death
Neonatal health: perinatal death, respiratory distress syndrome, infections, long-term outcomes
Other: nausea, vomiting, readmission

Pre-specified outcomes not reported in the systematic reviews
Febrile morbidity: thrombophlebitis, peritonitis, need for antibiotics
Haemorrhagic morbidity: anaemia
Pain: wound pain, pelvic pain, headache
Short-term recovery: bonding, ambulation, cope with baby without help, self-care without help, maternal depression
Long-term surgical complications: incisional hernia, wound dehiscence, intra-abdominal adhesions, sub-fertility, future complications
Acceptability
Severe morbidity: hysterectomy, visceral damage, shock, cardiac arrest, pulmonary oedema, respiratory failure, need for reanimation, seizures, encephalopathy, non-anaesthetic intubation, need for additional surgery, maternal near-miss
Neonatal health: stillbirth, Apgar score, neonatal trauma, neonatal death

Supplementary material 8. All procedures and outcomes

Intervention	Intervention arm	Control arm	Outcome	Effect estimates (n°studies/n°participants)	GRADE	Author	AMSTAR	Category	
**Pre-operative preparation**									
**Pre-anaesthetist evaluation**	-	-	-	-	-	-	-	no SR	
**Cardiovascular evaluation**	-	-	-	-	-	-	-	no SR	
**Laboratory tests**	-	-	-	-	-	-	-	no SR	
**Washing/ Bathing**	-	-	-	-	-	-	-	no SR	
**Shaving**	-	-	-	-	-	-	-	no SR	
**Fasting**	-	-	-	-	-	-	-	no SR	
**IV fluids**	-	-	-	-	-	-	-	no SR	
**Bladder evacuation**	Indwelling bladder catheter	No catheter	Distension of the bladder at the end of operation	RR 0.02 [0, 0.35] (1/420)	Low	Abdel-Aleem 2014	HIGH	PB	
**Bladder evacuation**	Indwelling bladder catheter	No catheter	Retention of urine	RR 0.06 [0.01, 0.47] (2/420)	Low	Abdel-Aleem 2014	HIGH	PB	
**Bladder evacuation**	Indwelling bladder catheter	No catheter	Need for catheterization	RR 0.03 [0.01, 0.16] (3/840)	Moderate	Abdel-Aleem 2014	HIGH	CEB	
**Bladder evacuation**	Indwelling bladder catheter	No catheter	Urinary Tract Infection – as defined by trialists	RR 20.21 [0.61, 670.22] (2/570)	Very Low	Abdel-Aleem 2014	HIGH	IE	
**Bladder evacuation**	Indwelling bladder catheter	No catheter	Postpartum haemorrhage due to uterine atony	RR 0.2 [0.02, 1.69] (2/420)	Low	Abdel-Aleem 2014	HIGH	IE	
**Bladder evacuation**	Indwelling bladder catheter	No catheter	Pain/discomfort due to catheterization (and/or at first voiding).	RR 10.47 [4.71, 23.25] (2/420)	Low	Abdel-Aleem 2014	HIGH	PH	

**Bladder evacuation**	Indwelling bladder catheter	No catheter	Time to patient ambulation (hours).	MD 4.34 [1.37, 7.31] (3/840)	Low	Abdel-Aleem 2014	HIGH	PH	
**Bladder evacuation**	Indwelling bladder catheter	No catheter	Length of hospital stay (days).	MD 0.62 [0.15, 1.1] (3/840)	Very Low	Abdel-Aleem 2014	HIGH	IE	
**Bladder evacuation**	Indwelling bladder catheter	No catheter	Time of first voiding after CS (hours)	MD 16.81 [16.32, 17.3] (1/420)	Moderate	Abdel-Aleem 2014	HIGH	CEH	
**Bladder evacuation**	Indwelling bladder catheter	In-out urethral catheter	Retention of urine	RR 0.32 [0.01, 7.8] (1/175)	Very Low	Abdel-Aleem 2014	HIGH	IE	
**Bladder evacuation**	Indwelling bladder catheter	In-out urethral catheter	Need for catheterization	RR 0.04 [0.00, 0.07]	Low	Abdel-Aleem 2014	HIGH	PB	
**Bladder evacuation**	Indwelling bladder catheter	In-out urethral catheter	UTI – as defined by trialists	RR 1.27 [0.58, 2.77] (2/225)	Very Low	Abdel-Aleem 2014	HIGH	IE	
**Bladder evacuation**	Indwelling bladder catheter	In-out urethral catheter	Time to patient ambulation (h)	MD 0.9 [0.25, 1.55] (1/175)	Very Low	Abdel-Aleem 2014	HIGH	IE	
**Bladder evacuation**	Indwelling bladder catheter	In-out urethral catheter	Length of hospital stay (days).	MD 0.09 [-0.44, 0.62] (1/175)	Low	Abdel-Aleem 2014	HIGH	IE	
**Urinary catheter removal**	Immediate urinary catheter removal	Delayed urinary catheter removal	Urinary retention	RR 3.62 [0.89, 14.71] (3/609)	Very Low	Menshawy 2018	LOW	IE	
**Urinary catheter removal**	Immediate urinary catheter removal	Delayed urinary catheter removal	Dysuria	RR 0.60 [0.38, 0.95] (3/609)	Moderate	Menshawy 2018	LOW	CEB	
**Urinary catheter removal**	Immediate urinary catheter removal	Delayed urinary catheter removal	Increased urinary frequency	RR 0.32 [0.16, 0.66] (3/609)	Moderate	Menshawy 2018	LOW	CEB	
**Urinary catheter removal**	Immediate urinary catheter removal	Delayed urinary catheter removal	Urgency	RR 0.49 [0.16, 1.52] (3/609)	Very low	Menshawy 2018	LOW	IE	

**Urinary catheter removal**	urinary catheter removal	Delayed urinary catheter removal	Significant bacteriuria	RR 0.49 [0.30, 0.83] (2/434)	Moderate	Menshawy 2018	LOW	CEB	✓
**Urinary catheter removal**	Immediate urinary catheter removal	Delayed urinary catheter removal	Post-operative oral rehydration	MD 0.32 [-0.90, 0.27] (2/434)	Low	Menshawy 2018	LOW	IE	?
**Urinary catheter removal**	Immediate urinary catheter removal	Delayed urinary catheter removal	Length of hospital stay	MD -10.54 [-23.68, 2.40] (3/609)	Very low	Menshawy 2018	LOW	IE	?
**Infection prevention**									
**Preoperative vaginal preparation**	Antiseptic solution	No preparation or saline preparation	Composite wound complication	RR 0.63 [0.37, 1.07] (2/729)	Moderate	Haas 2020	HIGH	IE	?
**Preoperative vaginal preparation**	Antiseptic solution	No preparation or saline preparation	Composite wound complication or endometritis	RR 0.46 [0.26, 0.82] (2/499)	Low	Haas 2020	HIGH	PB	✓
**Preoperative vaginal preparation**	Antiseptic solution - Women in labor	No preparation or saline preparation	Composite wound complication	RR 0.77 [0.36, 1.61] (2/314)	Low	Haas 2020	HIGH	IE	?
**Preoperative vaginal preparation**	Antiseptic solution - Women not in labor	No preparation or saline preparation	Composite wound complication	RR 0.54 [0.25, 1.16] (2/415)	Very low	Haas 2020	HIGH	IE	?
**Preoperative vaginal preparation**	Antiseptic solution - Women in labor	No preparation or saline preparation	Composite wound complication or endometritis	RR 0.34 [0.13, 0.87] (2/164)	Moderate	Haas 2020	HIGH	CEB	✓
**Preoperative vaginal preparation**	Antiseptic solution - Women not in labor	No preparation or saline preparation	Composite wound complication or endometritis	RR 0.60 [0.29, 1.26] (2/335)	Very low	Haas 2020	HIGH	IE	?
**Preoperative vaginal preparation**	Chlorhexidine-based solution	No preparation or saline preparation	Post-cesarean endometritis	RR 0.22 [0.07, 0.75] (1/214)	Low	Haas 2020	HIGH	PB	✓
**Preoperative vaginal preparation**	Chlorhexidine-based solution	No preparation or saline preparation	Postoperative fever	RR 0.48 [0.09, 2.56] (1/200)	Low	Haas 2020	HIGH	IE	?
**Preoperative vaginal preparation**	Chlorhexidine-based solution	No preparation or saline preparation	Surgical site infections (SSI)	RR 0.55 [0.17, 1.82] (1/200)	Moderate	Haas 2020	HIGH	IE	?
**Preoperative vaginal preparation**	Any antiseptic solution	No preparation or saline preparation	Endometritis	RR 0.41 [0.32, 0.52] (22/8912)	Moderate	Liu 2023	LOW	CEB	✓
**Preoperative vaginal preparation**	Any antiseptic solution	No preparation or saline preparation	Fever	RR 0.58 [0.45, 0.74] (16/5786)	Moderate	Liu 2023	LOW	CEB	✓
**Preoperative vaginal preparation**	Any antiseptic solution	No preparation or saline preparation	Surgical site infections (SSI)	RR 0.73 [0.59, 0.90] (20/8335)	Low	Liu 2023	LOW	PB	✓
**Preoperative vaginal preparation**	Iodine base	No preparation or saline preparation	Endometritis	RR 0.45 [0.35, 0.37] (19/8103)	Moderate	Liu 2023	LOW	CEB	✓
**Preoperative vaginal preparation**	Iodine base	No preparation or saline preparation	Fever	RR 0.58 [0.44, 0.77] (14/5284)	Low	Liu 2023	LOW	PB	✓

**Preoperative vaginal preparation**	Iodine base	No preparation or saline preparation	Surgical site infections (SSI)	RR 0.75 [0.60, 0.94] (18/783)	Low	Liu 2023	LOW	PB
**Preoperative vaginal preparation**	Guanidine base	No preparation or saline preparation	Endometritis	RR 0.22 [0.12, 0.40] (3/809)	High	Liu 2023	LOW	CEB
**Preoperative vaginal preparation**	Guanidine base	No preparation or saline preparation	Fever	RR 0.52 [0.21, 1.34] (2/502)	Moderate	Liu 2023	LOW	IE
**Preoperative vaginal preparation**	Guanidine base	No preparation or saline preparation	Surgical site infections (SSI)	RR 0.55 [0.26, 1.14] (2/502)	Low	Liu 2023	LOW	IE
**Preoperative vaginal preparation**	Antiseptic solution - stratified by presence of labor. Women in labor	No preparation or saline preparation	Endometritis	RR 0.32 [0.17, 0.60] (4/981)	Moderate	Liu 2023	LOW	CEB
**Preoperative vaginal preparation**	Antiseptic solution - Women in labor	No preparation or saline preparation	Fever	RR 0.62 [0.39, 1.01] (4/981)	Low	Liu 2023	LOW	IE
**Preoperative vaginal preparation**	Antiseptic solution - Women in labor	No preparation or saline preparation	Surgical site infections (SSI)	RR 0.56 [0.29, 1.08] (4/981)	Moderate	Liu 2023	LOW	IE
**Preoperative vaginal preparation**	Antiseptic solution - Women not in labor	No preparation or saline preparation	Endometritis	RR 0.63 [0.32, 1.22] (3/818)	Low	Liu 2023	LOW	IE
**Preoperative vaginal preparation**	Antiseptic solution - Women not in labor	No preparation or saline preparation	Fever	RR 0.93 [0.56,1.52] (3/818)	Low	Liu 2023	LOW	IE
**Preoperative vaginal preparation**	Antiseptic solution - Women not in labor	No preparation or saline preparation	Surgical site infections (SSI)	RR 0.67 [0.33, 1.35] (3/818)	Low	Liu 2023	LOW	IE
**Prophylactic antibiotics (use Y/N)**	Antibiotic prophylaxis – All types	No antibiotics prophylaxis	Maternal febrile morbidity/fever	RR 0.45 [0.40, 0.51] (56/9046)	Moderate	Smaill 2014	HIGH	CEB
**Prophylactic antibiotics (use Y/N)**	Antibiotic prophylaxis – All types	No antibiotics prophylaxis	Surgical site infections (SSI)	RR 0.40 [0.35, 0.46] (82/14407)	Moderate	Smaill 2014	HIGH	CEB
**Prophylactic antibiotics (use Y/N)**	Antibiotic prophylaxis – All types	No antibiotics prophylaxis	Maternal endometritis	RR 0.38 [0.34, 0.42] (83/13548)	Moderate	Smaill 2014	HIGH	CEB
**Prophylactic antibiotics (use Y/N)**	Antibiotic prophylaxis – All types	No antibiotics prophylaxis	Maternal serious infectious complications	RR 0.31 [0.20, 0.49] (32/6159)	Moderate	Smaill 2014	HIGH	CEB
**Prophylactic antibiotics (use Y/N)**	Antibiotic prophylaxis – All types	No antibiotics prophylaxis	Maternal urinary tract infection	RR 0.56 [0.49, 0.65] (66/10928)	Moderate	Smaill 2014	HIGH	CEB

**Prophylactic antibiotics (use Y/N)**	Antibiotic prophylaxis – All types	No antibiotics prophylaxis	Maternal adverse effects	RR 2.43 [1.00, 5.90] (13/2131)	Low	Smaill 2014	HIGH	IE	?
**Prophylactic antibiotics (use Y/N)**	Antibiotic prophylaxis – All types	No antibiotics prophylaxis	Maternal days in hospital	MD-0.46 [-0.65, -0.28] (19/3168)	Moderate	Smaill 2014	HIGH	CEB	✓
**Prophylactic antibiotics (use Y/N)**	Aminopenicillin s	No antibiotics prophylaxis	Maternal febrile morbidity/ fever	RR 0.39 [0.26, 0.58] (7/603)	Moderate	Smaill 2014	HIGH	CEB	✓
**Prophylactic antibiotics (use Y/N)**	Extended spectrum penicillins	No antibiotics prophylaxis	Maternal febrile morbidity/ fever	RR 0.37 [0.28, 0.49] (6/736)	Moderate	Smaill 2014	HIGH	CEB	✓
**Prophylactic antibiotics (use Y/N)**	Beta-lactamase inhibitor combinations	No antibiotics prophylaxis	Maternal febrile morbidity/ fever	RR 0.48 [0.29, 0.79] (5/791)	Moderate	Smaill 2014	HIGH	CEB	✓
**Prophylactic antibiotics (use Y/N)**	First generation cephalosporins	No antibiotics prophylaxis	Maternal febrile morbidity/ fever	RR 0.54 [0.44, 0.66] (10/1436)	Moderate	Smaill 2014	HIGH	CEB	✓
**Prophylactic antibiotics (use Y/N)**	Second generation cephalosporins	No antibiotics prophylaxis	Maternal febrile morbidity/ fever	RR 0.35 [0.27, 0.46] (9/1001)	Low	Smaill 2014	HIGH	PB	✓
**Prophylactic antibiotics (use Y/N)**	Cefamycins	No antibiotics prophylaxis	Maternal febrile morbidity/ fever	RR 0.73 [0.61, 0.88] (9/1894)	Low	Smaill 2014	HIGH	PB	✓
**Prophylactic antibiotics (use Y/N)**	Third generation cephalosporins	No antibiotics prophylaxis	Maternal febrile morbidity/ fever	RR 0.44 [0.27, 0.74] (3/376)	Low	Smaill 2014	HIGH	PB	✓
**Prophylactic antibiotics (use Y/N)**	Monobactams	No antibiotics prophylaxis	Maternal febrile morbidity/ fever	RR 0.64 [0.29, 1.42] (1/98)	Low	Smaill 2014	HIGH	IE	?
**Prophylactic antibiotics (use Y/N)**	Lincosamides	No antibiotics prophylaxis	Maternal febrile morbidity/ fever	RR 0.5 [0.08, 3.05] (1/30)	Low	Smaill 2014	HIGH	IE	?
**Prophylactic antibiotics (use Y/N)**	Nitroimidazoles	No antibiotics prophylaxis	Maternal febrile morbidity/ fever	RR 0.59 [0.48, 0.71] (7/1098)	Moderate	Smaill 2014	HIGH	CEB	✓
**Prophylactic antibiotics (use Y/N)**	Aminoglycoside -containing combination	No antibiotics prophylaxis	Maternal febrile morbidity/ fever	RR 0.33 [0.24, 0.46] (5/668)	Moderate	Smaill 2014	HIGH	CEB	✓
**Prophylactic antibiotics (use Y/N)**	Other antibiotics combination	No antibiotics prophylaxis	Maternal febrile morbidity/ fever	RR 0.27 [0.17, 0.44] (4/530)	Moderate	Smaill 2014	HIGH	CEB	✓
**Prophylactic antibiotics (use Y/N)**	Other regimens	No antibiotics prophylaxis	Maternal febrile morbidity/ fever	RR 0.23 [0.07, 0.76] (1/118)	Very Low	Smaill 2014	HIGH	IE	?

**Prophylactic antibiotics (use Y/N)**	Natural penicillins	No antibiotics prophylaxis	Surgical site infections (SSI)	RR 0.43 [0.07, 2.87] (1/66)	Very Low	Smaill 2014	HIGH	IE	?
**Prophylactic antibiotics (use Y/N)**	Aminopenicillin s	No antibiotics prophylaxis	Surgical site infections (SSI)	RR 0.5 [0.35, 0.72] (12/1323)	Moderate	Smaill 2014	HIGH	CEB	✔
**Prophylactic antibiotics (use Y/N)**	Extended spectrum penicillins	No antibiotics prophylaxis	Surgical site infections (SSI)	RR 0.18 [0.09, 0.39] (7/845)	Moderate	Smaill 2014	HIGH	CEB	✔
**Prophylactic antibiotics (use Y/N)**	Beta-lactamase inhibitor combinations	No antibiotics prophylaxis	Surgical site infections (SSI)	RR 0.26 [0.13, 0.51] (6/823)	Moderate	Smaill 2014	HIGH	CEB	✔
**Prophylactic antibiotics (use Y/N)**	First generation cephalosporins	No antibiotics prophylaxis	Surgical site infections (SSI)	RR 0.38 [0.28, 0.53] (17/3371)	Moderate	Smaill 2014	HIGH	CEB	✔
**Prophylactic antibiotics (use Y/N)**	Second generation cephalosporins	No antibiotics prophylaxis	Surgical site infections (SSI)	RR 0.38 [0.19, 0.75] (9/1166)	Moderate	Smaill 2014	HIGH	CEB	✔
**Prophylactic antibiotics (use Y/N)**	Cefamycins	No antibiotics prophylaxis	Surgical site infections (SSI)	RR 0.45 [0.33, 0.6] (16/2635)	Moderate	Smaill 2014	HIGH	CEB	✔
**Prophylactic antibiotics (use Y/N)**	Third generation cephalosporins	No antibiotics prophylaxis	Surgical site infections (SSI)	RR 0.44 [0.26, 0.73] (6/1519)	Moderate	Smaill 2014	HIGH	CEB	✔
**Prophylactic antibiotics (use Y/N)**	Monobactams	No antibiotics prophylaxis	Surgical site infections (SSI)	RR 0.48 [0.05, 5.03] (1/98)	Low	Smaill 2014	HIGH	IE	?
**Prophylactic antibiotics (use Y/N)**	Lincosamides	No antibiotics prophylaxis	Surgical site infections (SSI)	RR 0.25 [0.03, 2.44] (1/30)	Very Low	Smaill 2014	HIGH	IE	?
**Prophylactic antibiotics (use Y/N)**	Nitroimidazoles	No antibiotics prophylaxis	Surgical site infections (SSI)	RR 0.49 [0.34, 0.69] (9/1074)	Moderate	Smaill 2014	HIGH	CEB	✔
**Prophylactic antibiotics (use Y/N)**	Aminoglycoside -containing combination	No antibiotics prophylaxis	Surgical site infections (SSI)	RR 0.17 [0.08, 0.34] (8/654)	Moderate	Smaill 2014	HIGH	CEB	✔
**Prophylactic antibiotics (use Y/N)**	Other antibiotics combination	No antibiotics prophylaxis	Surgical site infections (SSI)	RR 0.6 [0.36, 1.02] (4/530)	Low	Smaill 2014	HIGH	IE	?
**Prophylactic antibiotics (use Y/N)**	Other regimens	No antibiotics prophylaxis	Surgical site infections (SSI)	RR 0.58 [0.15, 2.3] (2/171)	Very Low	Smaill 2014	HIGH	IE	?
**Prophylactic antibiotics (use Y/N)**	Natural penicillins	No antibiotics prophylaxis	Maternal endometritis	RR 0.19 [0.05, 0.65] (1/66)	Low	Smaill 2014	HIGH	PB	✔

**Prophylactic antibiotics (use Y/N)**	Aminopenicillins	No antibiotics prophylaxis	Maternal endometritis	RR 0.24 [0.16, 0.38] (10/1108)	Moderate	Smaill 2014	HIGH	CEB	
**Prophylactic antibiotics (use Y/N)**	Extended spectrum penicillins	No antibiotics prophylaxis	Maternal endometritis	RR 0.46 [0.37, 0.58] (9/1164)	Moderate	Smaill 2014	HIGH	CEB	
**Prophylactic antibiotics (use Y/N)**	Beta-lactamase inhibitor combinations	No antibiotics prophylaxis	Maternal endometritis	RR 0.67 [0.27, 1.66] (5/788)	Low	Smaill 2014	HIGH	IE	
**Prophylactic antibiotics (use Y/N)**	First generation cephalosporins	No antibiotics prophylaxis	Maternal endometritis	RR 0.42 [0.33, 0.54] (18/3451)	Moderate	Smaill 2014	HIGH	CEB	
**Prophylactic antibiotics (use Y/N)**	Second generation cephalosporins	No antibiotics prophylaxis	Maternal endometritis	RR 0.27 [0.2, 0.37] (13/1563)	Moderate	Smaill 2014	HIGH	CEB	
**Prophylactic antibiotics (use Y/N)**	Cefamycins	No antibiotics prophylaxis	Maternal endometritis	RR 0.36 [0.28, 0.47] (15/2535)	Moderate	Smaill 2014	HIGH	CEB	
**Prophylactic antibiotics (use Y/N)**	Third generation cephalosporins	No antibiotics prophylaxis	Maternal endometritis	RR 0.28 [0.11, 0.69] (4/408)	Low	Smaill 2014	HIGH	PB	
**Prophylactic antibiotics (use Y/N)**	Monobactams	No antibiotics prophylaxis	Maternal endometritis	RR 0.63 [0.25, 1.54] (1/98)	Low	Smaill 2014	HIGH	IE	
**Prophylactic antibiotics (use Y/N)**	Nitroimidazoles	No antibiotics prophylaxis	Maternal endometritis	RR 0.52 [0.37, 0.73] (8/919)	Low	Smaill 2014	HIGH	PB	
**Prophylactic antibiotics (use Y/N)**	Trimethoprim-sulfamethoxazole	No antibiotics prophylaxis	Maternal endometritis	RR 0.45 [0.2, 1.01] (1/57)	Very Low	Smaill 2014	HIGH	IE	
**Prophylactic antibiotics (use Y/N)**	Aminoglycoside-containing combination	No antibiotics prophylaxis	Maternal endometritis	RR 0.29 [0.19, 0.45] (8/722)	Moderate	Smaill 2014	HIGH	CEB	
**Prophylactic antibiotics (use Y/N)**	Other antibiotics combination	No antibiotics prophylaxis	Maternal endometritis	RR 0.33 [0.14, 0.75] (4/530)	Low	Smaill 2014	HIGH	PB	
**Prophylactic antibiotics (use Y/N)**	Other regimens	No antibiotics prophylaxis	Maternal endometritis	RR 0.42 [0.17, 1.03] (2/171)	Very Low	Smaill 2014	HIGH	IE	
**Prophylactic antibiotics (use Y/N)**	Aminopenicillins	No antibiotics prophylaxis	Maternal serious infectious complications	RR 0.27 [0.06, 1.18] (4/542)	Very Low	Smaill 2014	HIGH	IE	
**Prophylactic antibiotics (use Y/N)**	Extended spectrum penicillins	No antibiotics prophylaxis	Maternal serious infectious complications	RR 0.49 [0.05, 5.33] (1/151)	Very Low	Smaill 2014	HIGH	IE	

**Prophylactic antibiotics (use Y/N)**	First generation cephalosporins	No antibiotics prophylaxis	Maternal serious infectious complications	RR 0.39 [0.16, 0.95] (10/2351)	Low	Smaill 2014	HIGH	PB	✔
**Prophylactic antibiotics (use Y/N)**	Second generation cephalosporins	No antibiotics prophylaxis	Maternal serious infectious complications	RR 0.34 [0.06, 2.13] (5/522)	Very Low	Smaill 2014	HIGH	IE	?
**Prophylactic antibiotics (use Y/N)**	Cefamycins	No antibiotics prophylaxis	Maternal serious infectious complications	RR 0.22 [0.1, 0.49] (10/1372)	Very Low	Smaill 2014	HIGH	IE	?
**Prophylactic antibiotics (use Y/N)**	Third generation cephalosporins	No antibiotics prophylaxis	Maternal serious infectious complications	RR 0.7 [0.12, 4.03] (3/376)	Very Low	Smaill 2014	HIGH	IE	?
**Prophylactic antibiotics (use Y/N)**	Monobactams	No antibiotics prophylaxis	Maternal serious infectious complications	RR 1.25 [0.06, 25.02] (1/98)	Low	Smaill 2014	HIGH	IE	?
**Prophylactic antibiotics (use Y/N)**	Nitroimidazoles	No antibiotics prophylaxis	Maternal serious infectious complications	RR 0.47 [0.13, 1.65] (3/516)	Low	Smaill 2014	HIGH	IE	?
**Prophylactic antibiotics (use Y/N)**	Aminoglycoside -containing combination	No antibiotics prophylaxis	Maternal serious infectious complications	RR 0.32 [0.06, 1.59] (3/398)	Very Low	Smaill 2014	HIGH	IE	?
**Prophylactic antibiotics (use Y/N)**	Aminopenicillin s	No antibiotics prophylaxis	Maternal urinary tract infection	RR 0.6 [0.41, 0.9] (9/1039)	Low	Smaill 2014	HIGH	PB	✔
**Prophylactic antibiotics (use Y/N)**	Extended spectrum penicillins	No antibiotics prophylaxis	Maternal urinary tract infection	RR 0.34 [0.2, 0.58] (6/571)	Low	Smaill 2014	HIGH	PB	✔
**Prophylactic antibiotics (use Y/N)**	Beta-lactamase inhibitor combinations	No antibiotics prophylaxis	Maternal urinary tract infection	RR 0.64 [0.41, 0.99] (5/788)	Low	Smaill 2014	HIGH	PB	✔
**Prophylactic antibiotics (use Y/N)**	First generation cephalosporins	No antibiotics prophylaxis	Maternal urinary tract infection	RR 0.67 [0.49, 0.93] (17/1371)	Low	Smaill 2014	HIGH	PB	✔
**Prophylactic antibiotics (use Y/N)**	Second generation cephalosporins	No antibiotics prophylaxis	Maternal urinary tract infection	RR 0.58 [0.38, 0.89] (9/1166)	Low	Smaill 2014	HIGH	PB	✔
**Prophylactic antibiotics (use Y/N)**	Cefamycins	No antibiotics prophylaxis	Maternal urinary tract infection	RR 0.73 [0.54, 1] (14/2434)	Low	Smaill 2014	HIGH	IE	?
**Prophylactic antibiotics (use Y/N)**	Third generation cephalosporins	No antibiotics prophylaxis	Maternal urinary tract infection	RR 0.29 [0.04, 2.16] (3/308)	Very Low	Smaill 2014	HIGH	IE	?
**Prophylactic antibiotics (use Y/N)**	Monobactams	No antibiotics prophylaxis	Maternal urinary tract infection	RR 1.44 [0.35, 5.91] (1/98)	Low	Smaill 2014	HIGH	IE	?

**Prophylactic antibiotics (use Y/N)**	Nitroimidazoles	No antibiotics prophylaxis	Maternal urinary tract infection	RR 1.03 [0.53, 2.01] (5/622)	Low	Smaill 2014	HIGH	IE	?
**Prophylactic antibiotics (use Y/N)**	Aminoglycoside -containing combination	No antibiotics prophylaxis	Maternal urinary tract infection	RR 0.3 [0.15, 0.6] (5/266)	Moderate	Smaill 2014	HIGH	CEB	✔
**Prophylactic antibiotics (use Y/N)**	Other antibiotics combination	No antibiotics prophylaxis	Maternal urinary tract infection	RR 0.57 [0.32, 1.03] (2/237)	Low	Smaill 2014	HIGH	IE	?
**Prophylactic antibiotics (use Y/N)**	Other regimens	No antibiotics prophylaxis	Maternal urinary tract infection	RR 0.16 [0.02, 1.29] (2/171)	Very Low	Smaill 2014	HIGH	IE	?
**Prophylactic antibiotics (use Y/N)**	Extended spectrum penicillins	No antibiotics prophylaxis	Maternal adverse effects	RR 2.96 [0.12, 71.38] (1/139)	Very Low	Smaill 2014	HIGH	IE	?
**Prophylactic antibiotics (use Y/N)**	Beta-lactamase inhibitor combinations	No antibiotics prophylaxis	Maternal adverse effects	Not estimable (2/235)	Very Low	Smaill 2014	HIGH	IE	?
**Prophylactic antibiotics (use Y/N)**	First generation cephalosporins	No antibiotics prophylaxis	Maternal adverse effects	RR 0.37 [0.15, 0.9] (3/507)	Very Low	Smaill 2014	HIGH	IE	?
**Prophylactic antibiotics (use Y/N)**	Second generation cephalosporins)	No antibiotics prophylaxis	Maternal adverse effects	RR 1.54 [0.07, 36.11] (1/60)	Very Low	Smaill 2014	HIGH	IE	?
**Prophylactic antibiotics (use Y/N)**	Cefamycins	No antibiotics prophylaxis	Maternal adverse effects	RR 1.96 [0.41, 9.34] (5/654)	Very Low	Smaill 2014	HIGH	IE	?
**Prophylactic antibiotics (use Y/N)**	Third generation cephalosporins	No antibiotics prophylaxis	Maternal adverse effects	RR 2.94 [0.12, 70.5] (1/99)	Very Low	Smaill 2014	HIGH	IE	?
**Prophylactic antibiotics (use Y/N)**	Aminoglycoside -containing combination	Aminoglycoside-containing combination	Maternal adverse effects	RR 3 [0.13, 71.51] (1/80)	Very Low	Smaill 2014	HIGH	IE	?
**Prophylactic antibiotics (use Y/N)**	Aminopenicillin s	No antibiotics prophylaxis	Maternal days in hospital	MD -0.82 [-1.33, -0.31] (2/191)	Low	Smaill 2014	HIGH	PB	✔
**Prophylactic antibiotics (use Y/N)**	Beta-lactamase inhibitor combinations	No antibiotics prophylaxis	Maternal days in hospital	MD -0.18 [-0.46, 0.09] (3/555)	Very Low	Smaill 2014	HIGH	IE	?
**Prophylactic antibiotics (use Y/N)**	First generation cephalosporins	No antibiotics prophylaxis	Maternal days in hospital	MD 0.22 [-0.58, 0.14] (2/325)	Very Low	Smaill 2014	HIGH	IE	?
**Prophylactic antibiotics (use Y/N)**	Second generation cephalosporins	No antibiotics prophylaxis	Maternal days in hospital	MD -0.38 [-0.69, -0.08] (2/220)	Low	Smaill 2014	HIGH	PB	✔

**Prophylactic antibiotics (use Y/N)**	Cefamycins	No antibiotics prophylaxis	Maternal days in hospital	MD -0.37 [-0.6, -0.15] (6/1392)	Low	Smaill 2014	HIGH	PB	✓
**Prophylactic antibiotics (use Y/N)**	Nitroimidazoles	No antibiotics prophylaxis	Maternal days in hospital	MD -0.91 [-1.37, -0.45] (4/485)	Moderate	Smaill 2014	HIGH	CEB	✓
**Prophylactic antibiotics (use Y/N)**	Antibiotics prophylaxis - Elective CS	No antibiotics prophylaxis	Maternal febrile morbidity/fever	RR 0.48 [0.38, 0.61] (16/2537)	Moderate	Smaill 2014	HIGH	CEB	✓
**Prophylactic antibiotics (use Y/N)**	Antibiotics prophylaxis - Non-elective CS	No antibiotics prophylaxis	Maternal febrile morbidity/fever	RR 0.44 [0.37, 0.51] (15/1784)	Moderate	Smaill 2014	HIGH	CEB	✓
**Prophylactic antibiotics (use Y/N)**	Antibiotics prophylaxis – Elective and non-elective CS	No antibiotics prophylaxis	Maternal febrile morbidity/fever	RR 0.45 [0.4, 0.5] (29/4725)	Moderate	Smaill 2014	HIGH	CEB	✓
**Prophylactic antibiotics (use Y/N)**	Antibiotics prophylaxis - Elective CS	No antibiotics prophylaxis	Surgical site infections (SSI)	RR 0.62 [0.47, 0.82] (17/3537)	Moderate	Smaill 2014	HIGH	CEB	✓
**Prophylactic antibiotics (use Y/N)**	Antibiotics prophylaxis – Elective and non-elective CS	No antibiotics prophylaxis	Surgical site infections (SSI)	RR 0.39 [0.27, 0.58] (20/2291)	Low	Smaill 2014	HIGH	PB	✓
**Prophylactic antibiotics (use Y/N)**	Antibiotics prophylaxis – Elective and non-elective CS	No antibiotics prophylaxis	Surgical site infections (SSI)	RR 0.34 [0.28, 0.4] (49/8579)	Moderate	Smaill 2014	HIGH	CEB	✓
**Prophylactic antibiotics (use Y/N)**	Antibiotics prophylaxis - Elective CS	No antibiotics prophylaxis	Maternal endometritis	RR 0.38 [0.24, 0.61] (15/2502)	Moderate	Smaill 2014	HIGH	CEB	✓
**Prophylactic antibiotics (use Y/N)**	Antibiotics prophylaxis - Non-elective CS	No antibiotics prophylaxis	Maternal endometritis	RR 0.39 [0.33, 0.47] (20/2310)	Moderate	Smaill 2014	HIGH	CEB	✓
**Prophylactic antibiotics (use Y/N)**	Antibiotics prophylaxis – Elective and non-elective CS	No antibiotics prophylaxis	Maternal endometritis	RR 0.37 [0.32, 0.42] (52/8776)	Moderate	Smaill 2014	HIGH	CEB	✓
**Prophylactic antibiotics (use Y/N)**	Antibiotics prophylaxis - Elective CS	No antibiotics prophylaxis	Maternal serious infectious complications	RR 1.01 [0.04, 24.21] (4/545)	Low	Smaill 2014	HIGH	IE	?
**Prophylactic antibiotics (use Y/N)**	Antibiotics prophylaxis - Non-elective CS	No antibiotics prophylaxis	Maternal serious infectious complications	RR 0.27 [0.12, 0.65] (6/696)	Moderate	Smaill 2014	HIGH	CEB	✓
**Prophylactic antibiotics (use Y/N)**	Antibiotics prophylaxis – Elective and non-elective CS	No antibiotics prophylaxis	Maternal serious infectious complications	RR 0.32 [0.19, 0.54] (24/4918)	Moderate	Smaill 2014	HIGH	CEB	✓

**Prophylactic antibiotics (use Y/N)**	Antibiotics prophylaxis - Elective CS	No antibiotics prophylaxis	Maternal urinary tract infection	RR 0.92 [0.57, 1.5] (12/1936)	Very Low	Smaill 2014	HIGH	IE	?
**Prophylactic antibiotics (use Y/N)**	Antibiotics prophylaxis - Non-elective CS	No antibiotics prophylaxis	Maternal urinary tract infection	RR 0.44 [0.31, 0.6] (17/1981)	Moderate	Smaill 2014	HIGH	CEB	✓
**Prophylactic antibiotics (use Y/N)**	Antibiotics prophylaxis – Elective and non-elective CS	No antibiotics prophylaxis	Maternal urinary tract infection	RR 0.59 [0.49, 0.7] (41/7043)	Moderate	Smaill 2014	HIGH	CEB	✓
**Prophylactic antibiotics (use Y/N)**	Antibiotics prophylaxis - Elective CS	No antibiotics prophylaxis	Maternal adverse effects	Not estimable (2/235)	Very Low	Smaill 2014	HIGH	IE	?
**Prophylactic antibiotics (use Y/N)**	Antibiotics prophylaxis - Non-elective CS	No antibiotics prophylaxis	Maternal adverse effects	RR 2.86 [0.61, 13.31] (5/808)	Very Low	Smaill 2014	HIGH	IE	?
**Prophylactic antibiotics (use Y/N)**	Antibiotics prophylaxis – Elective and non-elective CS	No antibiotics prophylaxis	Maternal adverse effects	RR 2.23 [0.75, 6.63] (6/1088)	Very Low	Smaill 2014	HIGH	IE	?
**Prophylactic antibiotics (use Y/N)**	Antibiotics prophylaxis - Elective CS	No antibiotics prophylaxis	Maternal days in hospital	MD -0.41 [-0.62, -0.21] (5/1065)	Moderate	Smaill 2014	HIGH	CEB	✓
**Prophylactic antibiotics (use Y/N)**	Antibiotics prophylaxis - Non-elective CS	No antibiotics prophylaxis	Maternal days in hospital	MD -0.46 [-0.78, -0.14] (4/646)	Moderate	Smaill 2014	HIGH	CEB	✓
**Prophylactic antibiotics (use Y/N)**	Antibiotics prophylaxis – Elective and non-elective CS	No antibiotics prophylaxis	Maternal days in hospital	MD -0.39 [-0.57, -0.21] (11/1668)	Low	Smaill 2014	HIGH	PB	✓
**Prophylactic antibiotics (use Y/N)**	Any antibiotics before cord clamping	No antibiotics prophylaxis	Maternal febrile morbidity/ fever	RR 0.49 [0.42, 0.56] (26/3560)	Moderate	Smaill 2014	HIGH	CEB	✓
**Prophylactic antibiotics (use Y/N)**	Any antibiotics before cord clamping	No antibiotics prophylaxis	Maternal febrile morbidity/ fever	RR 0.44 [0.39, 0.50] (25/5095)	Moderate	Smaill 2014	HIGH	CEB	✓
**Prophylactic antibiotics (use Y/N)**	Any antibiotics - timing of administration not define	No antibiotics prophylaxis	Maternal febrile morbidity/ fever	RR 0.24 [0.15, 0.38] (5/391)	Moderate	Smaill 2014	HIGH	CEB	✓

**Prophylactic antibiotics (use Y/N)**	Any antibiotics before cord clamping	No antibiotics prophylaxis	Surgical site infections (SSI)	RR 0.39 [0.32, 0.47] (37/5593)	Moderate	Smaill 2014	HIGH	CEB	
**Prophylactic antibiotics (use Y/N)**	Any antibiotics - after cord clamping	No antibiotics prophylaxis	Surgical site infections (SSI)	RR 0.41 [0.34, 0.50] (42/8428)	Moderate	Smaill 2014	HIGH	CEB	
**Prophylactic antibiotics (use Y/N)**	Any antibiotics timing of administration not define	No antibiotics prophylaxis	Surgical site infections (SSI)	RR 0.30 [0.11, 0.85] (5/385)	Low	Smaill 2014	HIGH	PB	
**Prophylactic antibiotics (use Y/N)**	Any antibiotics - before cord clamping	No antibiotics prophylaxis	Maternal endometritis	RR 0.33 [0.26, 0.40] (32/4965)	Moderate	Smaill 2014	HIGH	CEB	
**Prophylactic antibiotics (use Y/N)**	Any antibiotics - after cord clamping	No antibiotics prophylaxis	Maternal endometritis	RR 0.40 [0.36, 0.46] (48/8213)	Moderate	Smaill 2014	HIGH	CEB	
**Prophylactic antibiotics (use Y/N)**	Any antibiotics timing of administration not define	No antibiotics prophylaxis	Maternal endometritis	RR 0.30 [0.18, 0.50] (5/409)	Moderate	Smaill 2014	HIGH	CEB	
**Prophylactic antibiotics (use Y/N)**	Any antibiotics before cord clamping	No antibiotics prophylaxis	Maternal serious infectious complications	RR 0.27 [0.12, 0.64] (13/2194)	Low	Smaill 2014	HIGH	PB	
**Prophylactic antibiotics (use Y/N)**	Any antibiotics - after cord clamping	No antibiotics prophylaxis	Maternal serious infectious complications	RR 0.32 [0.19, 0.55] (19/3893)	Moderate	Smaill 2014	HIGH	CEB	
**Prophylactic antibiotics (use Y/N)**	Any antibiotics timing of administration not define	No antibiotics prophylaxis	Maternal serious infectious complications	RR 0.36 [0.04, 3.32] (1/71)	Very Low	Smaill 2014	HIGH	IE	
**Prophylactic antibiotics (use Y/N)**	Any antibiotics before cord clamping	No antibiotics prophylaxis	Maternal urinary tract infections	RR 0.61 [0.50, 0.74] (30/4443)	Moderate	Smaill 2014	HIGH	CEB	
**Prophylactic antibiotics (use Y/N)**	Any antibiotics - after cord clamping	No antibiotics prophylaxis	Maternal urinary tract infections	RR 0.54 [0.43, 0.68] (34/6166)	Moderate	Smaill 2014	HIGH	CEB	
**Prophylactic antibiotics (use Y/N)**	Any antibiotics timing of administration not define	No antibiotics prophylaxis	Maternal urinary tract infections	RR 0.36 [0.18, 0.75] (4/350)	Moderate	Smaill 2014	HIGH	CEB	
**Prophylactic antibiotics (use Y/N)**	Any antibiotics before cord clamping	No antibiotics prophylaxis	Maternal adverse effects	RR 2.96 [0.12, 71.38] (2/339)	Very Low	Smaill 2014	HIGH	IE	

**Prophylactic antibiotics (use Y/N)**	Any antibiotics - after cord clamping	No antibiotics prophylaxis	Maternal adverse effects	RR 2.44 [0.88, 6.75] (8/1617)	Very Low	Smaill 2014	HIGH	IE	
**Prophylactic antibiotics (use Y/N)**	Any antibiotics timing of administration not define	No antibiotics prophylaxis	Maternal adverse effects	RR 2.17 [0.24, 19.70] (3/175)	Very Low	Smaill 2014	HIGH	IE	
**Prophylactic antibiotics (use Y/N)**	Any antibiotics before cord clamping	No antibiotics prophylaxis	Maternal days in hospital	MD -0.33 [-0.59, -0.08] (7/1060)	Moderate	Smaill 2014	HIGH	CEB	
**Prophylactic antibiotics (use Y/N)**	Any antibiotics - after cord clamping	No antibiotics prophylaxis	Maternal days in hospital	MD -0.40 [-0.55, -0.25] (10/2213)	Moderate	Smaill 2014	HIGH	CEB	
**Prophylactic antibiotics (use Y/N)**	Any antibiotics timing of administration not define	No antibiotics prophylaxis	Maternal days in hospital	MD -0.82 [-1.34, -0.31] (2/106)	Low	Smaill 2014	HIGH	PB	
**Prophylactic antibiotics (Type)**	Antistaphylococcal cephalosporins C1 and C2 (1st and 2nd generation)	Broad spectrum penicillins plus betalactamase inhibitors P2+	Maternal sepsis	RR 2.37 [0.10, 56.41] (1/75)	Very low	Williams 2021	HIGH	IE	
**Prophylactic antibiotics (Type)**	Antistaphylococcal cephalosporins C1 and C2 (1st and 2nd generation)	Broad spectrum penicillins plus betalactamase inhibitors P2+	Maternal endometritis	RR 1.10 [0.76, 1.60] (7/1161)	Low	Williams 2021	HIGH	IE	
**Prophylactic antibiotics (Type)**	Antistaphylococcal cephalosporins C1 and C2 (1st and 2nd generation)	Broad spectrum penicillins plus betalactamase inhibitors P2+	Maternal fever (febrile morbidity)	RR 1.07 [0.65, 1.75] (3/678)	Low	Williams 2021	HIGH	IE	
**Prophylactic antibiotics (Type)**	Antistaphylococcal cephalosporins C1 and C2 (1st and 2nd generation)	Broad spectrum penicillins plus betalactamase inhibitors P2+	Surgical site infections (SSI)	RR 0.78 [0.32, 1.90] (4/543)	Very low	Williams 2021	HIGH	IE	
**Prophylactic antibiotics (Type)**	Antistaphylococcal cephalosporins C1 and C2 (1st and 2nd generation)	Broad spectrum penicillins plus betalactamase inhibitors P2+	Maternal urinary tract infection	RR 0.64 [0.11, 3.73] (4/496)	Very low	Williams 2021	HIGH	IE	

**Prophylactic antibiotics (Type)**	Antistaphylococcal cephalosporins C1 and C2 (1st and 2nd generation)	Broad spectrum penicillins plus betalactamase inhibitors P2+	Maternal composite adverse effects	RR 0.96 [0.09, 10.50] (2/468)	Very low	Williams 2021	HIGH	IE	
**Prophylactic antibiotics (Type)**	Antistaphylococcal cephalosporins C1 and C2 (1st and 2nd generation)	Broad spectrum penicillins plus betalactamase inhibitors P2+	Maternal allergic reactions	Not estimable (2/373)	Very low	Williams 2021	HIGH	IE	
**Prophylactic antibiotics (Type)**	Antistaphylococcal cephalosporins C1 and C2 (1st and 2nd generation)	Broad spectrum penicillins plus betalactamase inhibitors P2+	Maternal skin rash	RR 1.08 [0.28, 4.11] (3/591)	Low	Williams 2021	HIGH	IE	
**Prophylactic antibiotics (Type)**	Antistaphylococcal cephalosporins C1 and C2 (1st and 2nd generation)	Broad spectrum penicillins plus betalactamase inhibitors P2+	Maternal endometritis	RR 0.91 [0.49, 1.66] (6/2147)	Low	Williams 2021	HIGH	IE	
**Prophylactic antibiotics (Type)**	Antistaphylococcal cephalosporins C1 and C2 (1st and 2nd generation)	Broad spectrum penicillins plus betalactamase inhibitors P2+	Maternal fever (febrile morbidity)	RR 0.74 [0.39, 1.41] (5/798)	Low	Williams 2021	HIGH	IE	
**Prophylactic antibiotics (Type)**	Antistaphylococcal cephalosporins C1 and C2 (1st and 2nd generation)	Broad spectrum penicillins plus betalactamase inhibitors P2+	Surgical site infections (SSI)	RR 1.15 [0.59, 2.26] (5/915)	Low	Williams 2021	HIGH	IE	
**Prophylactic antibiotics (Type)**	Antistaphylococcal cephalosporins C1 and C2 (1st and 2nd generation)	Broad spectrum penicillins plus betalactamase inhibitors P2+	Maternal urinary tract infection	RR 1.36 [0.59, 3.14] (4/515)	Low	Williams 2021	HIGH	IE	
**Prophylactic antibiotics (Type)**	Antistaphylococcal cephalosporins C1 and C2 (1st	Broad spectrum penicillins plus betalactamase inhibitors P2+	Maternal composite adverse effects	RR 2.02 [0.18, 21.96] (2/1698)	Very low	Williams 2021	HIGH	IE	

	and 2nd generation)								
**Prophylactic antibiotics (Type)**	Antistaphylococcal cephalosporins C1 and C2 (1st and 2nd generation)	Broad spectrum penicillins plus betalactamase inhibitors P2+	Maternal allergic reactions	Not estimable (2/329)	Very low	Williams 2021	HIGH	IE	
**Prophylactic antibiotics (Type)**	Antistaphylococcal cephalosporins C1 and C2 (1st and 2nd generation)	Broad spectrum penicillins plus betalactamase inhibitors P2+	Maternal length of hospital stay (days)	MD -1.50 [-2.46, -0.54] (1/132)	Low	Williams 2021	HIGH	PB	
**Prophylactic antibiotics (Type)**	Minimally antistaphylococcal cephalosporins C3 (3rd generation)	Non-antistaphylococcal penicillins P1 and P2 (natural and broad spectrum)	Maternal sepsis	Not estimable (1/59)	Very low	Williams 2021	HIGH	IE	
**Prophylactic antibiotics (Type)**	Minimally antistaphylococcal cephalosporins C3 (3rd generation)	Non-antistaphylococcal penicillins P1 and P2 (natural and broad spectrum)	Maternal endometritis	RR 1.74 [1.10, 2.75] (2/562)	Moderate	Williams 2021	HIGH	CEH	
**Prophylactic antibiotics (Type)**	Minimally antistaphylococcal cephalosporins C3 (3rd generation)	Non-antistaphylococcal penicillins P1 and P2 (natural and broad spectrum)	Maternal fever (febrile morbidity)	RR 0.89 [0.29, 2.76] (1/114)	Very low	Williams 2021	HIGH	IE	
**Prophylactic antibiotics (Type)**	Minimally antistaphylococcal cephalosporins C3 (3rd generation)	Non-antistaphylococcal penicillins P1 and P2 (natural and broad spectrum)	Surgical site infections (SSI)	RR 0.41 [0.13, 1.28] (3/406)	Very low	Williams 2021	HIGH	IE	
**Prophylactic antibiotics (Type)**	Minimally antistaphylococcal cephalosporins C3 (3rd generation)	Non-antistaphylococcal penicillins P1 and P2 (natural and broad spectrum)	Maternal urinary tract infection	RR 0.54 [0.05, 5.75] (2/173)	Very low	Williams 2021	HIGH	IE	
**Prophylactic antibiotics (Type)**	Minimally antistaphylococcal	Non-antistaphylococcal penicillins P1 and	Maternal composite serious infectious complication	Not estimable (1/59)	Very low	Williams 2021	HIGH	IE	

	cephalosporins C3 (3rd generation)	P2 (natural and broad spectrum)							
**Prophylactic antibiotics (Type)**	Minimally antistaphylococcal cephalosporins C3 (3rd generation)	Non-antistaphylococcal penicillins P1 and P2 (natural and broad spectrum)	Maternal composite adverse effects	Not estimable (2/507)	Very low	Williams 2021	HIGH	IE	
**Prophylactic antibiotics (Type)**	Minimally antistaphylococcal cephalosporins C3 (3rd generation)	Non-antistaphylococcal penicillins P1 and P2 (natural and broad spectrum)	Maternal allergic reactions	Not estimable (1/59)	Very low	Williams 2021	HIGH	IE	
**Prophylactic antibiotics (Type)**	Minimally antistaphylococcal cephalosporins C3 (3rd generation)	Non-antistaphylococcal penicillins P1 and P2 (natural and broad spectrum)	Maternal nausea/vomiting	Not estimable (1/59)	Very low	Williams 2021	HIGH	IE	
**Prophylactic antibiotics (Type)**	Minimally antistaphylococcal cephalosporins C3 (3rd generation)	Non-antistaphylococcal penicillins P1 and P2 (natural and broad spectrum)	Maternal diarrhea/skin rush	Not estimable (1/59)	Very low	Williams 2021	HIGH	IE	
**Prophylactic antibiotics (Type)**	Minimally antistaphylococcal cephalosporins C3 (3rd generation)	Broad spectrum penicillins plus betalactamase inhibitors P2+	Maternal endometritis	RR 1.02 [0.07, 15.88] (2/865)	Very low	Williams 2021	HIGH	IE	
**Prophylactic antibiotics (Type)**	Minimally antistaphylococcal cephalosporins C3 (3rd generation)	Broad spectrum penicillins plus betalactamase inhibitors P2+	Maternal fever (febrile morbidity)	RR 1.18 [0.63, 2.22] (1/746)	Low	Williams 2021	HIGH	IE	
**Prophylactic antibiotics (Type)**	Minimally antistaphylococcal cephalosporins C3 (3rd generation)	Broad spectrum penicillins plus betalactamase inhibitors P2+	Surgical site infections (SSI)	RR 0.67 [0.10, 4.58] (2/865)	Very low	Williams 2021	HIGH	IE	

**Prophylactic antibiotics (Type)**	Minimally antistaphylococcal cephalosporins C3 (3rd generation)	Broad spectrum penicillins plus betalactamase inhibitors P2+	Maternal urinary tract infection	RR 0.51 [0.05, 5.46] (2/865)	Very low	Williams 2021	HIGH	IE	
**Prophylactic antibiotics (Type)**	Minimally antistaphylococcal cephalosporins C3 (3rd generation)	Broad spectrum penicillins plus betalactamase inhibitors P2+	Maternal composite serious infectious complication	Not estimable (1/746)	Very low	Williams 2021	HIGH	IE	
**Prophylactic antibiotics (Type)**	Minimally antistaphylococcal cephalosporins C3 (3rd generation)	Broad spectrum penicillins plus betalactamase inhibitors P2+	Maternal composite serious infectious complication	Not estimable (2/865)	Very low	Williams 2021	HIGH	IE	
**Prophylactic antibiotics (Type)**	Minimally antistaphylococcal cephalosporins C3 (3rd generation)	Broad spectrum penicillins plus betalactamase inhibitors P2+	Maternal allergic reactions	Not estimable (2/865)	Very low	Williams 2021	HIGH	IE	
**Prophylactic antibiotics (Type)**	Minimally antistaphylococcal cephalosporins C3 (3rd generation)	Broad spectrum penicillins plus betalactamase inhibitors P2+	Maternal nausea/vomiting/diarrhea/skin rush	Not estimable (1/119)	Very low	Williams 2021	HIGH	IE	
**Prophylactic antibiotics (Type)**	Minimally antistaphylococcal cephalosporins C3 (3rd generation)	Broad spectrum penicillins plus betalactamase inhibitors P2+	Maternal length of hospital stay	MD -0.01 [-0.12, 0.10] (1/746)	Low	Williams 2021	HIGH	IE	
**Prophylactic antibiotics (Type)**	Cephalosporins C3 (3rd generation)	Penicillins P2 and P3 (broad spectrum and antistaphyloccal)	Maternal endometritis	RR 2.00 [0.18, 21.71] (1/200)	Very low	Williams 2021	HIGH	IE	
**Prophylactic antibiotics (Type)**	Cephalosporins C3 (3rd generation)	Penicillins P2 and P3 (broad spectrum and antistaphyloccal)	Maternal fever (febrile morbidity)	RR 1.17 [0.41, 3.35] (1/200)	Very low	Williams 2021	HIGH	IE	
**Prophylactic antibiotics (Type)**	Cephalosporins C3 (3rd generation)	Penicillins P2 and P3 (broad spectrum	Surgical site infections (SSI)	RR 0.50 [0.05, 5.43] (1/200)	Very low	Williams 2021	HIGH	IE	

		and antistaphyloccal)							
**Prophylactic antibiotics (Type)**	Cephalosporins C3 (3rd generation)	Penicillins P2 and P3 (broad spectrum and antistaphyloccal)	Maternal vomiting	RR 7.00 [0.37, 133.78] (1/200)	Very low	Williams 2021	HIGH	IE	?
**Prophylactic antibiotics (Type)**	Cephalosporins C3 (3rd generation)	Penicillins P2 and P3 (broad spectrum and antistaphyloccal)	Maternal skin rush	RR 3.00 [0.12, 72.77] (1/200)	Very low	Williams 2021	HIGH	IE	?
**Prophylactic antibiotics (Type)**	Fluoroquinolone s F	Broad spectrum penicillin plus betalactamase inhibitors P2+	Maternal sepsis	RR 2.55 [0.11, 60.57] (1/72)	Very low	Williams 2021	HIGH	IE	?
**Prophylactic antibiotics (Type)**	Fluoroquinolone s F	Broad spectrum penicillin plus betalactamase inhibitors P2+	Maternal endometritis	RR 1.17 [0.68, 2.01] (1/72)	Very low	Williams 2021	HIGH	IE	?
**Prophylactic antibiotics (Type)**	Fluoroquinolone s F	Broad spectrum penicillin plus betalactamase inhibitors P2+	Surgical site infections (SSI)	RR 4.25 [0.21, 85.51] (1/72)	Very low	Williams 2021	HIGH	IE	?
**Prophylactic antibiotics (Type)**	Fluoroquinolone s F	Broad spectrum penicillin plus betalactamase inhibitors P2+	Maternal urinary tract infection	RR 0.09 [0.01, 1.69] (1/72)	Very low	Williams 2021	HIGH	IE	?
**Prophylactic antibiotics (Type)**	Fluoroquinolone s F	Broad spectrum penicillin plus betalactamase inhibitors P2+	Maternal sepsis	RR 1.08 [0.07, 16.63] (1/81)	Very low	Williams 2021	HIGH	IE	?
**Prophylactic antibiotics (Type)**	Fluoroquinolone s F	Broad spectrum penicillin plus betalactamase inhibitors P2+	Maternal endometritis	RR 1.29 [0.76, 2.19] (1/81)	Very low	Williams 2021	HIGH	IE	?
**Prophylactic antibiotics (Type)**	Fluoroquinolone s F	Broad spectrum penicillin plus betalactamase inhibitors P2+	Surgical site infections (SSI)	RR 2.15 [0.20, 22.82] (1/81)	Very low	Williams 2021	HIGH	IE	?
**Prophylactic antibiotics (Type)**	Fluoroquinolone s F	Broad spectrum penicillin plus betalactamase inhibitors P2+	Maternal urinary tract infection	Not estimable (1/81)	Very low	Williams 2021	HIGH	IE	?
**Prophylactic antibiotics (Type)**	Carbapenems Ca	Cephalosporins C3 (3rd generation)	Maternal endometritis	RR 1.18 [0.08, 17.82] (1/48)	Very low	Williams 2021	HIGH	IE	?

**Prophylactic antibiotics (Type)**	Carbapenems Ca	Cephalosporins C3 (3rd generation)	Maternal fever (febrile morbidity)	RR 0.59 [0.06, 6.09] (1/48)	Very low	Williams 2021	HIGH	IE	
**Prophylactic antibiotics (Type)**	Carbapenems Ca	Cephalosporins C3 (3rd generation)	Surgical site infections (SSI)	RR 0.39 [0.02, 9.15] (1/48)	Very low	Williams 2021	HIGH	IE	
**Prophylactic antibiotics (Type)**	Carbapenems Ca	Cephalosporins C3 (3rd generation)	Maternal urinary tract infection	Not estimable (1/48)	Very low	Williams 2021	HIGH	IE	
**Prophylactic antibiotics (Type)**	Macrolides M	Cephalosporins C1 (1st generation)	Maternal fever (febrile morbidity)	RR 7.00 [0.37, 130.69] (1/70)	Very low	Williams 2021	HIGH	IE	
**Prophylactic antibiotics (Type)**	Broad spectrum penicillin P2 plus antistaphylococcal penicillin P3 plus aminoglycoside A plus nitroimidazole N	Cephalosporin C3 (3rd generation)	Maternal endometritis	RR 1.07 [0.55, 2.10] (1/200)	Very low	Williams 2021	HIGH	IE	
**Prophylactic antibiotics (Type)**	Broad spectrum penicillin P2 plus antistaphylococcal penicillin P3 plus aminoglycoside A plus nitroimidazole N	Cephalosporin C3 (3rd generation)	Maternal endometritis	RR 17.0 [0.99, 290.62] (1/200)	Very low	Williams 2021	HIGH	IE	
**Prophylactic antibiotics (Type)**	Broad spectrum penicillin P2 plus antistaphylococcal penicillin P3 plus aminoglycoside A plus nitroimidazole N	Cephalosporin C3 (3rd generation)	Maternal fever (febrile morbidity)	RR 0.86 [0.30, 2.46] (1/200)	Very low	Williams 2021	HIGH	IE	
**Prophylactic antibiotics (Type)**	Broad spectrum penicillin P2 plus antistaphylococcal penicillin P3 plus aminoglycosides	Cephalosporin C3 (3rd generation)	Maternal fever (febrile morbidity)	RR 8.0 [1.89, 33.89] (1/200)	Low	Williams 2021	HIGH	PH	

	A plus nitroimidazole N								
**Prophylactic antibiotics (Type)**	Broad spectrum penicillin P2 plus antistaphylococcal penicillin P3 plus aminoglycoside A plus nitroimidazole N	Cephalosporin C3 (3rd generation)	Surgical site infections (SSI)	RR 1.14 [0.43, 3.03] (1/200)	Very low	Williams 2021	HIGH	IE	?
**Prophylactic antibiotics (Type)**	Broad spectrum penicillin P2 plus antistaphylococcal penicillin P3 plus aminoglycosides A plus nitroimidazole N	Cephalosporin C3 (3rd generation)	Maternal urinary tract infection	RR 1.36 [0.66, 2.82] (1/200)	Very low	Williams 2021	HIGH	IE	?
**Prophylactic antibiotics (Type)**	Broad spectrum penicillin P2 plus antistaphylococcal penicillin P3 plus aminoglycoside A plus nitroimidazole N	Cephalosporin C3 (3rd generation)	Maternal length of hospital stay	MD -0.11 [-0.37, 0.15] (1/200)	Very low	Williams 2021	HIGH	IE	?
**Prophylactic antibiotics (Type)**	Broad spectrum penicillin P2 plus antistaphylococcal penicillin P3 plus aminoglycoside A plus nitroimidazole N	Cephalosporin C3 (3rd generation)	Costs	MD 5.98 [4.28, 7.68] (1/200)	Low	Williams 2021	HIGH	PH	✖
**Prophylactic antibiotics (Type)**	Lincosamide L plus aminoglycoside A	Natural penicillin P1	Maternal endometritis	RR 1.46 [0.35, 6.15] (1/88)	Very Low	Williams 2021	HIGH	IE	?
**Prophylactic antibiotics (Type)**	Cephalosporin C1 (1st generation) plus nitroimidazole	Natural penicillin P1	Maternal endometritis	RR 2.70 [0.63, 11.55] (1/139)	Very Low	Williams 2021	HIGH	IE	?

**Prophylactic antibiotics (Type)**	Cephalosporin C2 (2nd generation) plus nitroimidazole N	Broad spectrum penicillin plus betalactamase inhibitors P2+	Maternal endometritis	RR 0.33 [0.01, 7.77] (1/83)	Very low	Williams 2021	HIGH	IE	
**Prophylactic antibiotics (Type)**	Lincosamide L plus aminoglycoside A	Natural penicillin P1	Surgical site infections (SSI)	RR 1.10 [0.16, 7.43] (1/88)	Very low	Williams 2021	HIGH	IE	
**Prophylactic antibiotics (Type)**	Cephalosporin C1 (1st generation) plus nitroimidazole	Broad spectrum penicillin P2	Surgical site infections (SSI)	RR 2.02 [0.42, 9.63] (1/139)	Very low	Williams 2021	HIGH	IE	
**Prophylactic antibiotics (Type)**	Cephalosporin C2 (2nd generation) plus nitroimidazole N	Broad spectrum penicillin plus betalactamase inhibitors 2+	Surgical site infections (SSI)	RR 0.98 [0.06, 15.09] (1/83)	Very low	Williams 2021	HIGH	IE	
**Prophylactic antibiotics (Type)**	Cephalosporin C1 (1st generation) plus nitroimidazole	Broad spectrum penicillin P2	Maternal fever (febrile morbidity)	RR 2.36 [0.84, 6.62] (1/139)	Very low	Williams 2021	HIGH	IE	
**Prophylactic antibiotics (Type)**	Cephalosporin C2 (2nd generation) plus nitroimidazole N	Broad spectrum penicillin plus betalactamase inhibitors P2+	Maternal fever (febrile morbidity)	RR 2.93 [0.63, 13.68] (1/83)	Very low	Williams 2021	HIGH	IE	
**Prophylactic antibiotics (Type)**	Cephalosporin C2 (2nd generation) plus nitroimidazole N	Broad spectrum penicillin plus betalactamase inhibitors P2+	Maternal urinary tract infection	Not estimable (1/83)	Very low	Williams 2021	HIGH	IE	
**Prophylactic antibiotics (Type)**	Aminoglycoside A plus nitroimidazole N	Natural penicillin P1 plus nitroimidazole N plus macrolide M	Maternal sepsis	RR 0.81 [0.29, 2.26] (1/241)	Very low	Williams 2021	HIGH	IE	
**Prophylactic antibiotics (Type)**	Cephalosporin C3 (3rd generation) plus nitroimidazole N	Natural penicillin P1 plus broad spectrum penicillin P2 plus nitroimidazole N plus amphenicol Am	Maternal sepsis	RR 3.21 [0.34, 30.45] (1/232)	Very low	Williams 2021	HIGH	IE	
**Prophylactic antibiotics (Type)**	Aminoglycoside A plus nitroimidazole N	Natural penicillin P1 plus nitroimidazole N plus macrolide M	Surgical site infections (SSI)	RR 3.23 [0.34, 30.64] (1/241)	Very low	Williams 2021	HIGH	IE	
**Prophylactic antibiotics (Type)**	Antistaphylococ cal cephalosporin C1 and C2 (1st	Non-antistaphylococcal penicillins P1 and P2 (natural and	Surgical site infections (SSI)	RR 2.00 [0.19, 21.61] (2/256)	Very low	Williams 2021	HIGH	IE	

	and 2nd generation) plus nitroimidazole N	broad spectrum) plus nitroimidazole N							
**Prophylactic antibiotics (Type)**	Cephalosporin C3 (3rd generation) plus nitroimidazole N	Natural penicillin P1 plus broad spectrum penicillin P2 plus nitroimidazole N plus amphenicol Am	Surgical site infections (SSI)	RR 1.29 [0.40, 4.10] (1/232)	Very low	Williams 2021	HIGH	IE	
**Prophylactic antibiotics (Type)**	Aminoglycoside A plus nitroimidazole N	Natural penicillin P1 plus nitroimidazole N plus macrolide M	Maternal urinary tract infection	RR 1.08 [0.07, 17.03] (1/241)	Very low	Williams 2021	HIGH	IE	
**Prophylactic antibiotics (Type)**	Antistaphylococcal cephalosporin C1 and C2 (1st and 2nd generation) plus nitroimidazole N	Non-antistaphylococcal penicillins P1 and P2 (natural and broad spectrum) plus nitroimidazole N	Maternal urinary tract infection	Not estimable (1/156)	Very low	Williams 2021	HIGH	IE	
**Prophylactic antibiotics (Type)**	Aminoglycoside A plus nitroimidazole N	Natural penicillin P1 plus nitroimidazole N plus macrolide M	Maternal length of hospital stay	MD -0.30 [-0.78, 0.18] (1/241)	Very low	Williams 2021	HIGH	IE	
**Prophylactic antibiotics (Type)**	Antistaphylococcal cephalosporin C1 and C2 (1st and 2nd generation) plus nitroimidazole N	Non-antistaphylococcal penicillins P1 and P2 (natural and broad spectrum) plus nitroimidazole N	Maternal length of hospital stay	MD -0.53 [-1.36, 0.30] (1/100)	Very low	Williams 2021	HIGH	IE	
**Prophylactic antibiotics (Type)**	Antistaphylococcal cephalosporin C1 and C2 (1st and 2nd generation) plus nitroimidazole N	Non-antistaphylococcal penicillins P1 and P2 (natural and broad spectrum) plus nitroimidazole N	Maternal endometritis	Not estimable (1/156)	Very low	Williams 2021	HIGH	IE	
**Prophylactic antibiotics (Type)**	Antistaphylococcal cephalosporin C1 and C2 (1st and 2nd generation) plus nitroimidazole N	Non-antistaphylococcal penicillins P1 and P2 (natural and broad spectrum) plus nitroimidazole N	Maternal fever (febrile morbidity)	RR 0.72 [0.13, 4.14] (1/100)	Very low	Williams 2021	HIGH	IE	

**Prophylactic antibiotics (Type)**	Cephalosporin C3 (3rd generation) plus nitroimidazole N	Natural penicillin P1 plus broad spectrum penicillin P2 plus nitroimidazole N plus amphenicol Am	Maternal fever (febrile morbidity)	RR 1.22 [0.46, 3.27] (1/232)	Very low	Williams 2021	HIGH	IE	
**Prophylactic antibiotics (Type)**	Antistaphylococcal cephalosporin C1 and C2 (1st and 2nd generation) plus nitroimidazole N	Non-antistaphylococcal penicillins P1 and P2 (natural and broad spectrum) plus nitroimidazole N	Maternal composite adverse effects	Not estimable (1/100)	Very low	Williams 2021	HIGH	IE	
**Prophylactic antibiotics (Type)**	Antistaphylococcal cephalosporin C1 and C2 (1st and 2nd generation) plus nitroimidazole N	Non-antistaphylococcal penicillins P1 and P2 (natural and broad spectrum) plus nitroimidazole N	Costs	MD -136.12 [-165.73, -106.51] (1/100)	Very low	Williams 2021	HIGH	IE	
**Prophylactic antibiotics (Type)**	(Irrigation/lavage) cephalosporins	Penicillins	Maternal endometritis	RR 0.95 [0.63, 1.43] (1/383)	Low	Williams 2021	HIGH	IE	
**Prophylactic antibiotics (Type)**	(Irrigation/lavage) cephalosporins	Penicillins	Maternal fever (febrile morbidity)	RR 0.95 [0.63, 1.43] (1/383)	Low	Williams 2021	HIGH	IE	
**Prophylactic antibiotics (Type)**	(Irrigation/lavage) cephalosporins	Penicillins	Surgical site infections (SSI)	RR 1.06 [0.27, 4.17] (1/383)	Very low	Williams 2021	HIGH	IE	
**Adding azithromycin to antibiotic prophylaxis**	Standard prophylactic antibiotic + azithromycin (any mode of administration)	Standard prophylactic antibiotic ±placebo (any mode of administration)	Endometritis	RR 0.62 [0.49, 0.79] (3/3209)	Low	Yang 2022	HIGH	PB	
	Standard prophylactic antibiotic + azithromycin (any mode of administration)	Standard prophylactic antibiotic ±placebo (any mode of administration)	Surgical site infections (SSI)	RR 0.40 [0.27, 0.58] (3/3113)	Low	Yang 2022	HIGH	PB	
**Preincision adjunctive prophylaxis**	Cefazolin with adjunctive prophylaxis	Cefazolin alone	SSI	RR 0.46 [0.34, 0.63] (3/2373)	High	Markewi 2021	HIGH	CEB	

	Cefazolin with adjunctive prophylaxis	Cefazolin alone	Febrile morbidity	RR 0.38 [0.11, 1.25] (2/2213)	Low	Markewi 2021	HIGH	IE	
	Cefazolin with adjunctive prophylaxis	Cefazolin alone	Hospital length of stay	MD -1.46 [-2.21, -0.71] (2/360)	Moderate	Markewi 2021	HIGH	CEB	
**Prophylactic antibiotics (route)**	Intravenous (IV)	Irrigation	Endometritis	RR 0.95 [0.70, 1.29] (8/966)	Very low	Nabhan 2016	HIGH	IE	
**Prophylactic antibiotics (route)**	Intravenous (IV)	Irrigation	Surgical site infections (SSI)	RR 0.49 [0.17, 1.43] (7/859)	Low	Nabhan 2016	HIGH	IE	
**Prophylactic antibiotics (route)**	Intravenous (IV)	Irrigation	Postpartum febrile morbidity	RR 0.87 [0.48, 1.60] (3/364)	Very low	Nabhan 2016	HIGH	IE	
**Prophylactic antibiotics (route)**	Intravenous (IV)	Irrigation	Urinary tract infection	RR 0.74 [0.25, 2.15] (5/660)	Low	Nabhan 2016	HIGH	IE	
**Prophylactic antibiotics (route)**	Intravenous (IV)	Irrigation	Serious infectious complication	Not estimable (1/81)	Very low	Nabhan 2016	HIGH	IE	
**Prophylactic antibiotics (route)**	Intravenous (IV)	Irrigation	Adverse events (maternal)	Not estimable (3/284)	Very low	Nabhan 2016	HIGH	IE	
**Prophylactic antibiotics (route)**	Intravenous (IV)	Irrigation	Maternal length of hospital stay (days)	MD 0.28 [-0.22, 0.79] (4/512)	Low	Nabhan 2016	HIGH	IE	
**Prophylactic antibiotics (route)**	Intravenous (IV)	Irrigation (sensitivity analysis)	Endometritis	RR 0.87 [0.62, 1.23] (5/574)	Low	Nabhan 2016	HIGH	IE	
**Prophylactic antibiotics (route)**	Intravenous (IV)	Irrigation (sensitivity analysis)	Surgical site infections (SSI)	RR 0.42 [0.11, 1.61] (4/467)	Low	Nabhan 2016	HIGH	IE	
**Prophylactic antibiotics (dose)**	Multiple doses	Single dose	Postpartum infectious morbidity	RR 0.95 [0.75, 1.20] (16/2695)	Very low	Pinto Lopes 2016	LOW	IE	
**Prophylactic antibiotics (dose)**	Multiple doses	Single dose	Endometritis	RR 1.03 [0.74, 1.42] (/1271)	Very low	Pinto Lopes 2016	LOW	IE	
**Prophylactic antibiotics (dose)**	Multiple doses	Single dose	Surgical site infections (SSI)	RR 1.22 [0.72, 2.08] (9/1294)	Very low	Pinto Lopes 2016	LOW	IE	

**Prophylactic antibiotics (dose)**	Multiple doses	Single dose	Urinary tract infection	RR 0.65 [0.34, 1.24] (9/1238)	Very low	Pinto Lopes 2016	LOW	IE	?
**Prophylactic antibiotics (dose)**	Multiple doses (Mezlocilin)	Single dose (Mezlocilin)	Postpartum infectious morbidity	RR 1.08 [0.71, 1.64] (3/328)	Very low	Pinto Lopes 2016	LOW	IE	?
**Prophylactic antibiotics (dose)**	Multiple doses (Piperacilin)	Single dose (Piperacilin)	Postpartum infectious morbidity	RR 1.17 [0.64, 2.12] (3/415)	Very low	Pinto Lopes 2016	LOW	IE	?
**Prophylactic antibiotics (dose)**	Multiple doses (Cefoxitin)	Single dose (Cefoxitin)	Postpartum infectious morbidity	RR 0.55 [0.29, 1.04] (3/517)	Very low	Pinto Lopes 2016	LOW	IE	?
**Prophylactic antibiotics (dose)**	Multiple doses (Cefotaxime)	Single dose (Cefotaxime)	Postpartum infectious morbidity	RR 1.12 [0.60, 2.12] (2/200)	Very low	Pinto Lopes 2016	LOW	IE	?
**Prophylactic antibiotics (dose)**	Multiple doses (Cefazolin)	Single dose (Cefazolin)	Postpartum infectious morbidity	RR 1.03 [0.71, 1.49] (2/459)	Very low	Pinto Lopes 2016	LOW	IE	?
**Prophylactic antibiotics (dose)**	Multiple doses (Cefazolin + metronidazole)	Single dose (Cefazolin + metronidazole)	Postpartum infectious morbidity	RR 1.00 [0.21, 4.72] (1/100)	Very low	Pinto Lopes 2016	LOW	IE	?
**Prophylactic antibiotics (dose)**	Multiple doses (Ampicillin + metronidazole)	Single dose (Ampicillin + metronidazole)	Postpartum infectious morbidity	RR 1.53 [0.57, 4.13] (1/176)	Very low	Pinto Lopes 2016	LOW	IE	?
**Prophylactic antibiotics (dose)**	Multiple doses (Gentamicin + metronidazole)	Single dose (Gentamicin + metronidazole)	Postpartum infectious morbidity	RR 1.33 [0.64, 2.76] (1/500)	Very low	Pinto Lopes 2016	LOW	IE	?
**Prophylactic antibiotics (dose)**	Multiple doses (Penicillins)	Single dose (Penicillins)	Postpartum infectious morbidity	RR 1.15 [0.83, 1.59] (7/919)	Very low	Pinto Lopes 2016	LOW	IE	?
**Prophylactic antibiotics (dose)**	Multiple doses (Cephalosporine s)	Single dose (Cephalosporines)	Postpartum infectious morbidity	RR 0.79 [0.54, 1.17] (8/1276)	Very low	Pinto Lopes 2016	LOW	IE	?

**Prophylactic antibiotics (dose)**	Multiple doses (Aminoglycoside)	Single dose (Aminoglycosides)	Postpartum infectious morbidity	RR 1.33 [0.64, 2.76] (1/500)	Very low	Pinto Lopes 2016	LOW	IE	
**Prophylactic antibiotics (dose)**	Multiple doses (Single antibiotic)	Single dose (Single antibiotic)	Postpartum infectious morbidity	RR 0.90 [0.69, 1.18] (13/1919)	Very low	Pinto Lopes 2016	LOW	IE	
**Prophylactic antibiotics (dose)**	Multiple doses (Combination of antibiotics)	Single dose (Combination of antibiotics)	Postpartum infectious morbidity	RR 1.34 [0.78, 2.32] (3/776)	Very low	Pinto Lopes 2016	LOW	IE	
**Prophylactic antibiotics (dose)**	Multiple doses (Low resource setting)	Single dose (Low resource setting)	Postpartum infectious morbidity	RR 1.26 [0.82, 1.94] (5/969)	Very low	Pinto Lopes 2016	LOW	IE	
**Prophylactic antibiotics (dose)**	Multiple doses (High resource setting)	Single dose (High resource setting)	Postpartum infectious morbidity	RR 0.88 [0.65, 1.18] (11/1726)	Very low	Pinto Lopes 2016	LOW	IE	
**Prophylactic antibiotics (dose)**	Multiple doses - Emergency CS	Single dose - Emergency CS	Postpartum infectious morbidity	RR 1.13 [0.80, 1.60] (5/1081)	Very low	Pinto Lopes 2016	LOW	IE	
**Prophylactic antibiotics (dose)**	Multiple doses - Elective CS	Single dose - Elective CS	Postpartum infectious morbidity	RR 1.11 [0.58, 2.11] (3/293)	Very low	Pinto Lopes 2016	LOW	IE	
**Prophylactic antibiotics (dose)**	Multiple doses - Both types of CS	Single dose - Both types of CS	Postpartum infectious morbidity	RR 0.86 [0.57, 1.28] (8/1321)	Very low	Pinto Lopes 2016	LOW	IE	
**Prophylactic antibiotics (Timing)**	Antibiotics before cord clamping	After cord clamping	Composite infectious morbidity	RR 0.72 [0.56, 0.92] (9/4342)	High	Bolling 2018	Critically Low	CEB	
**Prophylactic antibiotics (Timing)**	Antibiotics before cord clamping	After cord clamping	Endometritis and or endomyometritis	RR 0.52 [0.37, 0.72] (16/8027)	High	Zeng 2023	Critically Low	CEB	
**Prophylactic antibiotics (Timing)**	Antibiotics before cord clamping	After cord clamping	Urinary tract infection/cystitis/pyelonephritis	RR 1.01 [0.64, 1.59] (9/4088)	Moderate	Bolling 2018	Critically Low	IE	
**Prophylactic antibiotics (Timing)**	Antibiotics before cord clamping	After cord clamping	Respiratory tract infections	RR 1.37 [0.26, 7.07] (5/2049)	Low	Bolling 2018	Critically Low	IE	

**Prophylactic antibiotics (Timing)**	Antibiotics before cord clamping	After cord clamping	Fever	RR 0.96 [0.66, 1.39] (6/3030)	Moderate	Bolling 2018	Critically Low	IE	
**Prophylactic antibiotics (Timing)**	Antibiotics before cord clamping	After cord clamping	Surgical site infections (SSI)	RR 0.54 [0.42, 0.69] (16/8027)	High	Zeng 2023	LOW	CEB	
**Prophylactic antibiotics (Timing)**	Antibiotics before cord clamping	After cord clamping	Adverse events attributed to the antibiotic.	RR 0.33 [0.01, 8.08] (5/1517)	Low	Bolling 2018	Critically Low	IE	
**Prophylactic antibiotics (Timing)**	Antibiotics before cord clamping	After cord clamping	Hospital length of stay	MD -0.13 [-0.25, -0.01] (3/2083)	High	Bolling 2018	Critically Low	CEB	
**Prophylactic antibiotics (Timing)**	Antibiotics before cord clamping	After cord clamping	Sepsis	RR 0.77 [0.52, 1.13] (8/3690)	Moderate	Bolling 2018	Critically Low	IE	
**Prophylactic antibiotics (Timing)**	Antibiotics before cord clamping	After cord clamping	Suspected sepsis	RR 0.90 [0.70, 1.17] (7/2069)	Moderate	Bolling 2018	Critically Low	IE	
**Prophylactic antibiotics (Timing)**	Antibiotics before cord clamping	After cord clamping	Infection with an antibiotic resistant bacterium.	RR 0.70 [0.12, 4.14] (2/813)	Low	Bolling 2018	Critically Low	IE	
**Prophylactic antibiotics (Timing)**	Antibiotics before cord clamping	After cord clamping	Pelvic abscess	RR 1.00 [0.06, 15.97] (1/741)	Low	Mackeen 2014	HIGH	IE	
**Prophylactic antibiotics (Timing)**	Antibiotics before cord clamping	After cord clamping	Febrile illness	RR 0.79 [0.59, 1.05] (8/4607)	Moderate	Zeng 2023	LOW	IE	
**Prophylactic antibiotics (Timing)**	Antibiotics before cord clamping	After cord clamping	Neonatal sepsis	RR 0.76 [0.51, 1.13] (5/2907)	Moderate	Mackeen 2014	HIGH	IE	
**Prophylactic antibiotics (Timing)**	Antibiotics before cord clamping	After cord clamping	Neonatal sepsis	RR 0.83 [0.61, 1.14] (9/5130)	Moderate	Zeng 2023	LOW	IE	
**Prophylactic antibiotics (Timing)**	Antibiotics before cord clamping	After cord clamping	Infection (other)	RR 0.93 [0.52, 1.64] (1/302)	Low	Mackeen 2014	HIGH	IE	
**Prophylactic antibiotics (Timing)**	Antibiotics before cord clamping	After cord clamping	Neonatal antibiotic treatment	RR 0.68 [0.21, 2.16] (2/872)	Moderate	Bolling 2018	Critically Low	IE	
**Prophylactic antibiotics (Timing)**	Antibiotics before cord clamping	After cord clamping	Neonatal fever	RR 0.67 [0.28, 1.62] (1/953)	Low	Bolling 2018	Critically Low	IE	
**Prophylactic antibiotics (Timing)**	Antibiotics before cord clamping	After cord clamping	Intensive care unit admission	RR 0.89 [0.74, 1.08] (11/5294)	Moderate	Bolling 2018	Critically Low	IE	

**Prophylactic antibiotics (Timing)**	Antibiotics before cord clamping	After cord clamping	NICU admission	RR 0.94 [0.79, 1.12] (13/7131)	High	Zeng 2023	LOW	CEND	
**Prophylactic antibiotics (Timing)**	Antibiotics before cord clamping	After cord clamping	Length of intensive care unit stay	MD -1.33 [-5.32, 2.66] (3/1731)	Low	Bolling 2018	Critically Low	IE	
**Oxygen inspiration fraction**	High inspiration oxygen fraction: 60% to 90% oxygen perioperatively	Lower inspiration oxygen fraction: oxygen 30% to 40% oxygen perioperatively	Maternal mortality (overall)	RR 1.07 [0.87, 1.33] (8/4918)	Low	Wetterslev 2015	HIGH	IE	
**Oxygen inspiration fraction**	High inspiration oxygen fraction: 60% to 90% oxygen perioperatively	Lower inspiration oxygen fraction: oxygen 30% to 40% oxygen perioperatively	Surgical site infection	RR 0.87 [0.71, 1.07] (15/7219)	Low	Wetterslev 2015	HIGH	IE	
**Oxygen inspiration fraction**	High inspiration oxygen fraction: 60% to 90% oxygen perioperatively	Lower inspiration oxygen fraction: oxygen 30% to 40% oxygen perioperatively	Surgical site infection	RR 1.21 [0.91, 1.6] (4/1719)	Low	Wetterslev 2015	HIGH	IE	
**Oxygen inspiration fraction**	High inspiration oxygen fraction: 60% to 90% oxygen perioperatively	Lower inspiration oxygen fraction: oxygen 30% to 40% oxygen perioperatively	Respiratory insufficiency	RR 1.25 [0.79, 1.99] (3/1588)	Low	Wetterslev 2015	HIGH	IE	
**Oxygen inspiration fraction**	High inspiration oxygen fraction: 60% to 90% oxygen perioperatively	Lower inspiration oxygen fraction: oxygen 30% to 40% oxygen perioperatively	Serious adverse events	RR 0.96 [0.65, 1.43] (3/3588)	very low	Wetterslev 2015	HIGH	IE	
**Oxygen inspiration fraction**	High inspiration oxygen fraction: 60% to 90% oxygen perioperatively	Lower inspiration oxygen fraction: oxygen 30% to 40% oxygen perioperatively	Length of stay after surgery	MD -0.06 [-0.44, 0.32] (7/4702)	Low	Wetterslev 2015	HIGH	IE	
**Procedures to improve neonatal outcomes**									-
**Corticosteroids for preventing neonatal RDS**	Antenatal corticosteroids (betamethasone) at term	Usual care at term	Respiratory distress syndrome	RR 0.34 [0.07, 1.65] (1/942)	Low	Sotiriadis 2021	HIGH	IE	
**Corticosteroids for preventing neonatal RDS**	Antenatal corticosteroids (betamethasone)	Usual care (Birth before 38 + 0 weeks)	Respiratory distress syndrome	RR 0.33 [0.04, 3.09] (1/145)	Low	Sotiriadis 2021	HIGH	IE	

	(Birth before 38 + 0 weeks)								
**Corticosteroids for preventing neonatal RDS**	Antenatal corticosteroids (betamethasone) (Birth 38 + 0 to 38 + 6 weeks)	Usual care (Birth 38 + 0 to 38 + 6 weeks)	Respiratory distress syndrome	RR 0.32 [0.01, 7.80] (1/429)	Low	Sotiriadis 2021	HIGH	IE	
**Corticosteroids for preventing neonatal RDS**	Antenatal corticosteroids (betamethasone) (Birth at or after 39 + 0 weeks)	Usual care (Birth at or after 39 + 0 weeks)	Respiratory distress syndrome	RR 0.37 [0.02, 8.96] (1/368)	Low	Sotiriadis 2021	HIGH	IE	
**Corticosteroids for preventing neonatal RDS**	Antenatal corticosteroids (betamethasone) at term	Usual care at term	Transient tachypnoea of the neonate	RR 0.52 [0.25, 1.11] (1/942)	Low	Sotiriadis 2021	HIGH	IE	
**Corticosteroids for preventing neonatal RDS**	Antenatal corticosteroids (betamethasone) (Birth before 38 + 0 weeks)	Usual care (Birth before 38 + 0 weeks)	Transient tachypnoea of the neonate	RR 3.95 [0.45, 34.45] (1/145)	Low	Sotiriadis 2021	HIGH	IE	
**Corticosteroids for preventing neonatal RDS**	Antenatal corticosteroids (betamethasone) (Birth 38 + 0 to 38 + 6 weeks)	Usual care (Birth 38 + 0 to 38 + 6 weeks)	Transient tachypnoea of the neonate	RR 0.18 [0.05, 0.61] (1/429)	Moderate	Sotiriadis 2021	HIGH	CEB	
**Corticosteroids for preventing neonatal RDS**	Antenatal corticosteroids (betamethasone) (Birth at or after 39 + 0 weeks)	Usual care (Birth at or after 39 + 0 weeks)	Transient tachypnoea of the neonate	RR 1.65 [0.28, 9.78] (1/368)	Low	Sotiriadis 2021	HIGH	IE	
**Corticosteroids for preventing neonatal RDS**	Antenatal corticosteroids (betamethasone) at term	Usual care at term	Admission to neonatal special care (all levels) for respiratory morbidity	RR 0.45 [0.22, 0.90] (1/942)	Moderate	Sotiriadis 2021	HIGH	CEB	
**Corticosteroids for preventing neonatal RDS**	Antenatal corticosteroids (betamethasone) (Birth before 38 + 0 weeks)	Usual care (Birth before 38 + 0 weeks)	Admission to neonatal special care (all levels) for respiratory morbidity	RR 0.49 [0.16, 1.57] (1/145)	Low	Sotiriadis 2021	HIGH	IE	
**Corticosteroids for preventing neonatal RDS**	Antenatal corticosteroids (betamethasone) (Birth 38 + 0 to 38 + 6 weeks)	Usual care (Birth 38 + 0 to 38 + 6 weeks)	Admission to neonatal special care (all levels) for respiratory morbidity	RR 0.44 [0.17, 1.14] (1/429)	Low	Sotiriadis 2021	HIGH	IE	

**Corticosteroids for preventing neonatal RDS**	Antenatal corticosteroids (betamethasone) (Birth at or after 39 + 0 weeks)	Usual care (Birth at or after 39 + 0 weeks)	Admission to neonatal special care (all levels) for respiratory morbidity	RR 0.37 [0.04, 3.50] (1/368)	Low	Sotiriadis 2021	HIGH	IE	?
**Corticosteroids for preventing neonatal RDS**	Antenatal corticosteroids (betamethasone) at term	Usual care at term	Admission to neonatal intensive care unit for respiratory morbidity	RR 0.15 [0.03, 0.64] (1/942)	Low	Sotiriadis 2021	HIGH	PB	✓
**Corticosteroids for preventing neonatal RDS**	Antenatal corticosteroids (betamethasone) at term	Usual care at term	Need for mechanical ventilation	RR 4.07 [0.46, 36.27] (1/942)	Very low	Sotiriadis 2021	HIGH	IE	?
**Corticosteroids for preventing neonatal RDS**	Antenatal corticosteroids (betamethasone) at term	Usual care at term	Maternal development of postpartum infection	Not estimable (1/942)	Very low	Sotiriadis 2021	HIGH	IE	?
**Corticosteroids for preventing neonatal RDS**	Antenatal corticosteroids (betamethasone) (Birth before 38 + 0 weeks)	Usual care (Birth before 38 + 0 weeks)	Admission to neonatal special care (all levels) for any indication	RR 0.69 [0.28, 1.71] (1/145)	Very low	Sotiriadis 2021	HIGH	IE	?
**Corticosteroids for preventing neonatal RDS**	Antenatal corticosteroids (betamethasone) (Birth 38 + 0 to 38 + 6 weeks)	Usual care (Birth 38 + 0 to 38 + 6 weeks)	Admission to neonatal special care (all levels) for any indication	RR 0.78 [0.38, 1.58] (1/429)	Very low	Sotiriadis 2021	HIGH	IE	?
**Corticosteroids for preventing neonatal RDS**	Antenatal corticosteroids (betamethasone) (Birth at or after 39 + 0 weeks)	Usual care (Birth at or after 39 + 0 weeks)	Admission to neonatal special care (all levels) for any indication	RR 1.10 [0.36, 3.36] (1/368)	Very low	Sotiriadis 2021	HIGH	IE	?
**Corticosteroids for preventing neonatal RDS**	Antenatal corticosteroids (betamethasone) at term	Usual care at term	Neonatal infectious morbidity	Not estimable (1/942)	Very low	Sotiriadis 2021	HIGH	IE	?
**Corticosteroids for preventing neonatal RDS**	Antenatal corticosteroids (betamethasone) at term	Usual care at term	Perinatal deaths	Not estimable (1/942)	Very low	Sotiriadis 2021	HIGH	IE	?
**Corticosteroids for preventing neonatal RDS**	Antenatal corticosteroids (betamethasone) at term	Usual care at term	Length of stay in neonatal intensive care unit (days)	MD -2.14 [-2.50, -1.78] (1/942)	Low	Sotiriadis 2021	HIGH	PB	✓

**Corticosteroids for preventing neonatal RDS**	Antenatal corticosteroids (betamethasone) at term	Usual care at term	Readmission for respiratory problems after initial discharge	RR 0.66 [0.35, 1.25] (1/407)	Very low	Sotiriadis 2021	HIGH	IE	
**Corticosteroids for preventing neonatal RDS**	Antenatal corticosteroids (betamethasone) at term	Usual care at term	Cognitive impairment	RR 0.81 [0.49, 1.35] (1/407)	Very low	Sotiriadis 2021	HIGH	IE	
**Corticosteroids for preventing neonatal RDS**	Antenatal corticosteroids (betamethasone) at term	Usual care at term	Emotional and behavioral problems: measured with Strengths and difficulties questionnaire	MD 0.18 [-1.12, 1.48] (1/407)	Very low	Sotiriadis 2021	HIGH	IE	
**Corticosteroids for preventing neonatal RDS**	Antenatal corticosteroids (betamethasone) at term	Usual care at term	Adverse maternal effects of therapy	RR 15.26 [0.87, 266.36] (1/942)	Very low	Sotiriadis 2021	HIGH	IE	
**Prostaglandins**	Prostaglandin E2 gel	Placebo	Respiratory distress	RR 0.33 [0.01, 7.68] (1/36)	Low	Motaze 2013	HIGH	IE	
**Skin to skin contact**	Immediate or Early skin-to-skin	Standard contact for healthy infants	Breastfeeding 1 month to 4 months post birth	RR 1.22 [1.04, 1.44] (2/220)	Low	Moore 2016	HIGH	IE	
**Skin to skin contact**	Immediate or Early skin-to-skin	Standard contact for healthy infants	Exclusive breastfeeding at hospital discharge to 1 month post birth	RR 1.0 [0.53, 1.88] (1/34)	Low	Moore 2016	HIGH	IE	
**Skin to skin contact**	Immediate or Early skin-to-skin	Standard contact for healthy infants	Exclusive breastfeeding 6 weeks to 6 months post birth	RR 1.16 [0.95, 1.43] (2/144)	Very low	Moore 2016	HIGH	IE	
**Skin to skin contact**	Immediate or Early skin-to-skin	Standard contact for healthy infants	Success of the first breastfeeding (IBFAT score)	RR 1.37 [0.12, 2.62] (2/124)	Very low	Moore 2016	HIGH	IE	
**Skin to skin contact**	Immediate or Early skin-to-skin	Standard contact for healthy infants	Respiratory rate 75 minutes - 2 hours post birth	MD -4.48 [-9.20, 0.24] (1/32)	Low	Moore 2016	HIGH	IE	
**Skin to skin contact**	Immediate or Early skin-to-skin	Standard contact for healthy infants	Maternal pain 4 hours post-cesarean birth	MD -1.38 [-2.79, 0.03] (1/35)	Low	Moore 2016	HIGH	IE	
**Skin to skin contact**	Immediate or Early skin-to-skin	Standard contact for healthy infants	Maternal state anxiety 8 hours to 3 days post birth	MD -2.70 [-6.06, 0.66] (1/60)	Very low	Moore 2016	HIGH	IE	
**Postoperative recovery**									
**Time to fluids and food**	Early oral intake	Delay oral intake	Ileus symptom	RR 0.98 [0.71, 1.36] (8/1449)	Very low	Hsu 2013	CRIT LOW	IE	

**Time to fluids and food**	Early oral intake	Delay oral intake	Diarrohea	MD 0.62 [0.26, 1.45] (3/571)	Very low	Hsu 2013	CRIT LOW	IE	?
**Time to fluids and food**	Early oral intake	Delay oral intake	Time to first food (h)	MD -7.20 [-13.26, -1.14] (1/118)	Moderate	Mangesi 2002	LOW	CEB	✓
**Time to fluids and food**	Early oral intake -Anaesthesia mixed or not defined	Delay oral intake - Anaesthesia mixed or not defined	Postoperative nausea	RR 0.97 [0.49, 1.91] (1/118)	Low	Mangesi 2002	LOW	IE	?
**Time to fluids and food**	Early oral intake -Regional Analgesia	Delay oral intake - Regional Analgesia	Abdominal distension	RR 0.70 [0.40, 1.22]	Very low	Mangesi 2002	LOW	IE	?
**Time to fluids and food**	Early oral intake -Anaesthesia mixed or not defined	Delay oral intake - Anaesthesia mixed or not defined	Abdominal distension	RR 0.81 [0.52,1.27]	Moderate	Mangesi 2002	LOW	IE	?
**Time to fluids and food**	Early oral intake	Delay oral intake	Analgesic doses postoperatively	MD -0.50 [-1.83, 0.82]	Low	Mangesi 2002	LOW	IE	?
**Time to fluids and food**	Early oral intake -Regional Analgesia	Delay oral intake - Regional Analgesia	Analgesic doses postoperatively	MD -0.80 [-2.25, 0.65]	Low	Mangesi 2002	LOW	IE	?
**Time to fluids and food**	Early oral intake -Anaesthesia mixed or not defined	Delay oral intake - Anaesthesia mixed or not defined	Analgesic doses postoperatively	MD 1[-2.27, 4.27]	Very low	Mangesi 2002	LOW	IE	?
**Time to fluids and food**	Early oral intake -Regional anaesthesia	Delay oral intake - Regional anaesthesia	Time to passing flatus (h)	MD -4.04 [-10.41, 2.34] (5/1888)	Low	Guo 2015	LOW	IE	?
**Time to fluids and food**	Early oral intake -General anaesthesia	Delay oral intake - General anaesthesia	Time to passing flatus (h)	MD -15.91 [-23.32, -8.50] (4/711)	Moderate	Guo 2015	LOW	CEB	✓
**Time to fluids and food**	Early oral intake -Anaesthesia mixed or not defined	Delay oral intake - Anaesthesia mixed or not defined	Time to passing flatus (h)	MD 5.63 [-9.78, -1.48] (3/356)	Low	Guo 2015	LOW	PB	✓
**Time to fluids and food**	Early oral intake -Regional anaesthesia	Delay oral intake - Regional anaesthesia	Time to bowel movement (h)	MD -12.85 [-28.08, 2.39] (4/714)	Low	Guo 2015	LOW	IE	?
**Time to fluids and food**	Early oral intake -General anaesthesia	Delay oral intake - General anaesthesia	Time to bowel movement (h)	MD -14.06 [-15.36, -12.75] (3/511)	High	Guo 2015	LOW	CEB	✓
**Time to fluids and food**	Early oral intake -Regional anaesthesia	Delay oral intake - Regional anaesthesia	Length of hospital stay (h)	MD -13.16 [-19.97, -6.35] (9/2508)	Moderate	Guo 2015	LOW	CEB	✓

**Time to fluids and food**	Early oral intake -General anaesthesia	Delay oral intake - General anaesthesia	Length of hospital stay (h)	MD -26.47 [-43.78, -9.15] (5/893)	Moderate	Guo 2015	LOW	CEB	✓
**Time to fluids and food**	Early oral intake -Anaesthesia mixed or not defined	Delay oral intake - Anaesthesia mixed or not defined	Length of hospital stay (h)	MD -5.99 [-10.19, -1.78] (2/499)	Moderate	Guo 2015	LOW	CEB	✓
**Time to fluids and food**	Early oral intake	Delay oral intake	Score for patient satisfaction	MD 22.00 [-13.18, 57.18] (2/400)	Low	Guo 2015	LOW	IE	?
**Time to fluids and food**	Early oral intake -Regional anaesthesia	Delay oral intake - Regional anaesthesia	Score for patient satisfaction	MD 22.00 [-13.18, 57.18] (2/400)	Low	Guo 2015	LOW	IE	?
**Time to fluids and food**	Early oral intake	Delay oral intake	Time to first breastfeeding (h)	MD -9.81 [-15.35, -4.26] (3/519)	Moderate	Guo 2015	LOW	CEB	✓
**Time to fluids and food**	Early oral intake	Delay oral intake	Time to stop IV fluids	MD -8.88 [-16.65, -1.11] (2/1252)	Moderate	Kim 2021	LOW	CEB	✓
**Time to fluids and food**	Early oral intake	Delay oral intake	Return to bowel movement	MD -2.41 [-3.8, -1.02] (6/2332)	Moderate	Chen 2024	LOW	CEB	✓
**Time to fluids and food**	Early oral intake	Delay oral intake	Passage of flatus	MD -3.55 [-6.36, -0.75] (6/2242)	Moderate	Chen 2024	LOW	CEB	✓
**Time to fluids and food**	Early oral intake	Delay oral intake	Passage of faeces after surgery	MD -2.19 [-6.01, 1.62] (3/532)	Moderate	Chen 2024	Criticall y Low	IE	?
**Time to fluids and food**	Early oral intake	Delay oral intake	Vomiting	RR 1.08 [0.74, 1.57] (7/2282)	Low	Chen 2024	Criticall y Low	IE	?
**Time to fluids and food**	Early oral intake	Delay oral intake	Nausea	RR 1.21 [0.83, 1.77] (5/1743)	Low	Chen 2024	Criticall y Low	IE	?
**Time to fluids and food**	Early oral intake	Delay oral intake	Abdominal distension	RR 0.76 [0.31, 1.89] (4/1706)	Low	Chen 2024	Criticall y Low	IE	?
**Time to fluids and food**	Early oral intake	Delay oral intake	Ileus symptom	RR 0.91 [0.40,2.06] (2/1214)	Low	Chen 2024	Criticall y Low	IE	?
**Time to fluids and food**	Early oral intake	Delay oral intake	Fever	RR 0.87[0.53,1.44] (3/1514)	Moderate	Chen 2024	Criticall y Low	IE	?
**Time to fluids and food**	Early oral intake	Delay oral intake	Wound infection	RR 2.64 [0.71, 9.85] (4/1706)	Low	Chen 2024	Criticall y Low	IE	?
**Time to fluids and food**	Early oral intake	Delay oral intake	Urinary tract infection	RR 0.56 [0.12, 2.58] (2/340)	Low	Chen 2024	Criticall y Low	IE	?

**Time to fluids and food**	Early oral intake	Delay oral intake	Readmission	RR 1.75 [0.52, 5.91] (3/1514)	Very low	Chen 2024	Criticall y Low	IE	
**Time to fluids and food**	Early oral intake	Delay oral intake	Time to ambulation after surgery	MD -0.96 [-1.8, -0.13] (3/532)	High	Chen 2024	Criticall y Low	CEB	
**Time to fluids and food**	Early oral intake	Delay oral intake	Time to removal of catheter	MD -15.18 [-25.61, -4.74] (2/1510)	Moderate	Chen 2024	Criticall y Low	CEB	
**Time to fluids and food**	Early oral intake	Delay oral intake	Start regular diet	MD -7.03 [-13.13, -0.92] (3/738)	Moderate	Chen 2024	Criticall y Low	CEB	
**Time to fluids and food**	Early oral intake	Delay oral intake	Length of hospital stay	MD -4.57 [-9.63, 0.49] (5/1906)	Low	Chen 2024	Criticall y Low	IE	
**Time to fluids and food**	Chewing gum	Control	Time to first passage of flatus (h)	MD -7.09 [-9.27, -4.91] (13/2399)	Very low	Pereira Gomes Morais 2016	HIGH	IE	
**Time to fluids and food**	Chewing gum - More than 1 hour/day	Control	Time to first passage of flatus (h)	MD -7.3 [-10.24, -4.37] (9/1806)	Very low	Pereira Gomes Morais 2016	HIGH	IE	
**Time to fluids and food**	Chewing gum - Up to 1 hour/day	Control	Time to first passage of flatus (h)	MD -6.83 [-11.48, -2.17] (3/540)	Very low	Pereira Gomes Morais 2016	HIGH	IE	
**Time to fluids and food**	Chewing gum - No information on time chewing per day	Control	Time to first passage of flatus (h)	MD -5.67 [-10.35, -0.99] (1/53)	Very low	Pereira Gomes Morais 2016	HIGH	IE	
**Time to fluids and food**	Chewing gum	Control – early feeding	Time to first passage of flatus (h)	MD -5.9 [-10.67, -1.12] (2/450)	Very low	Pereira Gomes Morais 2016	HIGH	IE	
**Time to fluids and food**	Chewing gum	Control - conventional feeding	Time to first passage of flatus (h)	MD -7.78 [-11.09, -4.47] (8/1589)	Very low	Pereira Gomes Morais 2016	HIGH	IE	

**Time to fluids and food**	Chewing gum	Control - No information on comparator	Time to first passage of flatus (h)	MD -6.02 [-7.14, -4.9] (3/360)	Low	Pereira Gomes Morais 2016	HIGH	PB	
**Time to fluids and food**	Chewing gum - Elective CS	Control	Time to first passage of flatus (h)	MD -6.82 [-10.06, -3.58] (6/1233)	Very low	Pereira Gomes Morais 2016	HIGH	IE	
**Time to fluids and food**	Chewing gum - Non-elective CS	Control	Time to first passage of flatus (h)	MD -5.89 [-7.13, -4.65] (3/499)	Low	Pereira Gomes Morais 2016	HIGH	PB	
**Time to fluids and food**	Chewing gum – No information on type of CS	Control	Time to first passage of flatus (h)	MD -8.49 [-14.48, -2.5] (4/667)	Very low	Pereira Gomes Morais 2016	HIGH	IE	
**Time to fluids and food**	Chewing gum - initiation of gum chewing Immediately after CS	Control	Time to first passage of flatus (h)	MD 6.04 [-9.31, -2.77] (5/1086)	Very low	Pereira Gomes Morais 2016	HIGH	IE	
**Time to fluids and food**	Chewing gum - initiation of gum chewing to 5 h after CS	Control	Time to first passage of flatus (h)	MD -9.11 [-13.75, -4.47] (5/980)	Very low	Pereira Gomes Morais 2016	HIGH	IE	
**Time to fluids and food**	Chewing gum - initiation of gum chewing 6 h or more after CS	Control	Time to first passage of flatus (h)	MD -5.27 [-7.07, -3.47] (3/333)	Very low	Pereira Gomes Morais 2016	HIGH	IE	
**Time to fluids and food**	Chewing gum	Control	Proportion of participants with ileus	RR 0.39 [0.19, 0.8] (4/1139)	Moderate	Pereira Gomes Morais 2016	HIGH	CEB	
**Time to fluids and food**	Chewing gum - Elective CS	Control	Proportion of participants with ileus	RR 0.38 [0.02, 9.29] (1/200)	Very low	Pereira Gomes Morais 2016	HIGH	IE	

**Time to fluids and food**	Chewing gum - Non-elective CS	Control	Proportion of participants with ileus	RR 0.55 [0.34, 0.88] (2/439)	Moderate	Pereira Gomes Morais 2016	HIGH	CEB	✔
**Time to fluids and food**	Chewing gum - No info on type of CS	Control	Proportion of participants with ileus	RR 0.21 [0.08, 0.54] (1/500)	Low	Pereira Gomes Morais 2016	HIGH	PB	✔
**Time to fluids and food**	Chewing gum - Immediately after caesarean section	Control	Proportion of participants with ileus	RR 0.37 [0.15, 0.95] (2/886)	Very low	Pereira Gomes Morais 2016	HIGH	IE	?
**Time to fluids and food**	Chewing gum	Control	Proportion of participants with ileus 2 to 5 h after caesarean section	RR 0.38 [0.02, 9.29] (1/200)	Very low	Pereira Gomes Morais 2016	HIGH	IE	?
**Time to fluids and food**	Chewing gum	Control	Proportion of participants with ileus 6 h or more after caesarean section	Not estimable (1/53)	Very low	Pereira Gomes Morais 2016	HIGH	IE	?
**Time to fluids and food**	Chewing gum	Control	Time to passage of faeces (h)	MD -9.22 [-11.49, -6.95] (11/2016)	Very low	Pereira Gomes Morais 2016	HIGH	IE	?
**Time to fluids and food**	Chewing gum	Control	Duration of hospital stay (days)	MD -0.36 [-0.53, -0.18] (7/1489)	Low	Pereira Gomes Morais 2016	HIGH	PB	✔
**Time to fluids and food**	Chewing gum	Control	Need for additional analgesics/antiemetics.	RR 0.5 [0.12, 2.13] (3/726)	Very low	Pereira Gomes Morais 2016	HIGH	IE	?
**Time to fluids and food**	Chewing gum	Control	Time to first hearing of normal intestinal sounds (h)	MD -4.56 [-6.18, -2.93] (9/1729)	Low	Pereira Gomes Morais 2016	HIGH	PB	✔

**Use of Abdominal binder**	Use of the abdominal binder	Non-users of the abdominal binder	VAS score after 24 h	MD -1.76 [-3.14, -0.39] (6/702)	Moderate	Abd-ElGawad 2020	HIGH	CEB	
**Use of Abdominal binder**	Use of the abdominal binder	Non-users of the abdominal binder	VAS score after 48 h	MD -1.21 [-1.51, -0.90] (3/422)	High	Abd-ElGawad 2020	HIGH	CEB	
**Use of Abdominal binder**	Use of the abdominal binder	Non-users of the abdominal binder	Distress score after 24 h	MD -1.87 [-3.01, -0.73] (4/506)	Moderate	Abd-ElGawad 2020	HIGH	CEB	
**Use of Abdominal binder**	Use of the abdominal binder	Non-users of the abdominal binder	Distress score after 48 h	MD -1.87 [-3.07, -0.67] (4/506)	Moderate	Abd-ElGawad 2020	HIGH	CEB	
**Discharge and post-discharge care**									
**Time to discharge**	Early discharge	Standard discharge	Infants readmitted for neonatal morbidity within 7 days	RR 1.15 [0.42, 3.16] (2/247)	Very low	Jones 2021	HIGH	IE	
**Time to discharge**	Early discharge	Standard discharge	Infants readmitted for neonatal morbidity within 28 days	RR 1.59 [1.27, 1.98] (10/6918)	Very low	Jones 2021	HIGH	IE	
**Time to discharge**	Early discharge	Standard discharge	Infants readmitted for neonatal morbidity within 28 days: Caesarean birth	RR 1.57 [1.24, 1.99] (4/3605)	Moderate	Jones 2021	HIGH	CEH	
**Time to discharge**	Early discharge (< 24h)	Standard discharge (>24h)	Infants readmitted for neonatal morbidity within 28 days	RR 1.64 [1.28, 2.09] (3/3770)	Moderate	Jones 2021	HIGH	CEH	
**Time to discharge**	Early discharge (< 24h)	Standard discharge (>24h)	Infants readmitted for neonatal morbidity within 28 days	RR 1.28 [0.73, 2.25] (6/3060)	Very low	Jones 2021	HIGH	IE	
**Time to discharge**	Early discharge (< 24h)	Standard discharge (>24h)	Infants readmitted for neonatal morbidity within 28 days	RR 5.00 [0.25, 101.25] (1/88)	Very low	Jones 2021	HIGH	IE	
**Time to discharge**	Early discharge - CS	Standard discharge	Women readmitted within six weeks	RR 2.03 [0.37, 10.95] (6/459)	Low	Jones 2021	HIGH	IE	
**Time to discharge**	Early discharge - CS	Standard discharge	Women probably depressed within six months	RR 0.80 [0.46, 1.42] (5/4333)	Low	Jones 2021	HIGH	IE	
**Time to discharge**	Early discharge (< 24h)	Standard discharge (>24h)	Women probably depressed within six months: subgroups < 24 h	RR 0.62 [0.18, 2.16] (3/3770)	Very Low	Jones 2021	HIGH	IE	
**Time to discharge**	Early discharge	Standard discharge	Women breastfeeding (exclusively or partially) at six weeks postpartum	RR 1.04 [0.96, 1.13] (10/7156)	Low	Jones 2021	HIGH	PEND	

**Time to discharge**	Early discharge - CS	Standard discharge	Women breastfeeding (exclusively or partially) at six weeks postpartum	RR 0.99 [0.83, 1.18] (2/334)	Low	Jones 2021	HIGH	PEND	=
**Time to discharge**	Early discharge (< 24h)	Standard discharge (>24h)	Women breastfeeding (exclusively or partially) at six weeks postpartum	RR 1.04 [0.96, 1.13] (10/7156)	Very low	Jones 2021	HIGH	IE	?
**Time to discharge**	Early discharge	Standard discharge	Women breastfeeding (exclusively or partially) at 12 weeks postpartum	RR 1.21 [1.03, 1.41] (1/430)	Moderate	Jones 2021	HIGH	CEB	✔
**Time to discharge**	Early discharge (< 24h)	Standard discharge (>24h)	Women breastfeeding (partially or exclusively) at six months postpartum	RR 1.01 [0.90, 1.15] (3/3770)	Very low	Jones 2021	HIGH	IE	?
**Time to discharge**	Early discharge (< 24h)	Standard discharge (>24h)	Women breastfeeding (partially or exclusively) at six months postpartum	RR 1.12 [0.77, 1.63] (4/2886)	Very low	Jones 2021	HIGH	IE	?
**Time to discharge**	Early discharge (< 24h)	Standard discharge (>24h)	Women breastfeeding (partially or exclusively) at six months postpartum (	RR 1.10 [0.90, 1.34] (3/520)	Very low	Jones 2021	HIGH	IE	?
**Time to discharge**	Early discharge	Standard discharge	Women breastfeeding (partially or exclusively) at six months postpartum	RR 1.11 [0.87, 1.43] (3/973)	Low	Jones 2021	HIGH	IE	?
**Time to discharge**	Early discharge	Standard discharge	Infant mortality within 28 days	RR 0.39 [0.04, 3.74] (2/4884)	Very Low	Jones 2021	HIGH	IE	?
**Time to discharge**	Early discharge	Standard discharge	Infant mortality within one year	RR 0.45 [0.07, 2.77] (2/1986)	Very low	Jones 2021	HIGH	IE	?
**Time to discharge**	Early discharge	Standard discharge	Number of contacts with healthcare professionals regarding infant health issues within four weeks of birth	RR 0.88 [0.67, 1.16] (6/639)	Moderate	Jones 2021	HIGH	IE	?
**Time to discharge**	Early discharge	Standard discharge	Number of contacts with healthcare professionals regarding maternal health issues within six weeks of birth	RR 0.72 [0.43, 1.20] (2/464)	Moderate	Jones 2021	HIGH	IE	?
**Time to discharge**	Early discharge	Standard discharge	Women reporting health problems (including perineal pain, perineal infection, breast soreness, breast infection, caesarean wound pain, caesarean Surgical site infections (SSI)) in the first six weeks postpartum	RR 0.25 [0.11, 0.59] (1/200)	Low	Jones 2021	HIGH	PB	✔
**Time to discharge**	Early discharge	Standard discharge	Women reporting infant feeding problems	RR 0.89 [0.43, 1.86] (2/2405)	Very low	Jones 2021	HIGH	IE	?
**Time to discharge**	Early discharge	Standard discharge	Women satisfied with postnatal care - dichotomous data	RR 1.10 [0.95, 1.29] (4/3908)	Very Low	Jones 2021	HIGH	IE	?

**Time to discharge**	Early discharge	Standard discharge	Satisfaction with postnatal care - continuous data	RR 0.74 [0.50, 0.98] (2/306)	Very low	Jones 2021	HIGH	IE	?
**Time to discharge**	Early discharge	Standard discharge	Women who perceive their length of hospital stay as too short	RR 2.00 [0.19, 21.21] (1/82)	Very low	Jones 2021	HIGH	IE	?
**Time to discharge**	Early discharge	Standard discharge	Women perceive their length of hospital stay as too long	RR 0.56 [0.20, 1.52] (1/82)	Very low	Jones 2021	HIGH	IE	?
**Time to discharge**	Early discharge	Standard discharge	Maternal readmission within 6 weeks	RR 1.10 [0.8, 1.32] (6/4119)	Moderate	Chaaranti 2024	HIGH	IE	?
**Time to discharge**	Early discharge (24-28hs)	Standard discharge (>=48hs)	Maternal readmission within 6 weeks	RR 1.14 [0.81,1.59] (4/3777)	Moderate	Chaaranti 2024	HIGH	IE	?
**Post-discharge controls**	Schedules involving more home visits (more than 4	Fewer home visits- (more than 4 visits)	Maternal mortality within 42 days	RR 0.39 [0.02, 9.41] (1/225)	Very low	Yonemoto 2021	HIGH	IE	?
**Post-discharge controls**	Schedules involving more	Fewer home visits	Neonatal mortality	R 0.99 [0.26, 3.69] (3/1281)	Very low	Yonemoto 2021	HIGH	IE	?
**Post-discharge controls**	Home visits	No home visits	Neonatal mortality	RR 3.06 [0.37, 25.39] (2/873)	Very Low	Yonemoto 2021	HIGH	IE	?
**Post-discharge controls**	Schedules involving more home visits (4 or more)	Fewer home visits (less than 4)	Neonatal mortality	RR 0.48 [0.09, 2.60] (1/408)	Very Low	Yonemoto 2021	HIGH	IE	?
**Post-discharge controls**	Schedules involving more	Fewer home visits	Severe maternal morbidity	RR 0.96 [0.81, 1.15] (3/1228)	Very Low	Yonemoto 2021	HIGH	IE	?
**Post-discharge controls**	Home visits	No home visits	Severe maternal morbidity	RR 0.97 [0.80, 1.17] (2/876)	Low	Yonemoto 2021	HIGH	PEND	=
**Post-discharge controls**	Schedules involving more home visits (4 or more)	Fewer home visits (less than 4)	Severe maternal morbidity	RR 0.90 [0.52, 1.54] (1/352)	Low	Yonemoto 2021	HIGH	IE	?
**Post-discharge controls**	Home visits	No home visits	PPH	RR 0.78 [0.49, 1.26] (2/873)	Very low	Yonemoto 2021	HIGH	IE	?
**Post-discharge controls**	Home visits	No home visits	Abdominal pain up to 42 days	RR 1.06 [0.83, 1.34] (2/869)	Very low	Yonemoto 2021	HIGH	IE	?
**Post-discharge controls**	Home visits	No home visits	Back pain up to 42 days	RR 0.96 [0.83, 1.11] (2/871)	Low	Yonemoto 2021	HIGH	PEND	=

**Post-discharge controls**	Home visits	No home visits	Urinary tract complications up to 42	RR 0.83 [0.63, 1.10] (2/876)	Very low	Yonemoto 2021	HIGH	IE	
**Post-discharge controls**	Home visits	No home visits	Maternal fever up to 42 days postpartum	RR 1.30 [0.93, 1.82] (2/876)	Very low	Yonemoto 2021	HIGH	IE	
**Post-discharge controls**	Schedules involving more home visits	Fewer home visits	Dyspareunia	RR 1.18 [0.90, 1.55] (2/869)	Very low	Yonemoto 2021	HIGH	IE	
**Post-discharge controls**	Schedules involving more home visits	Fewer home visits	Maternal satisfaction with postnatal care	RR 0.96 [0.90, 1.02] (2/862)	Low	Yonemoto 2021	HIGH	PEND	
**Post-discharge controls**	Schedules involving more home visits (more than 4	Fewer home visits- (more than 4 visits)	Maternal satisfaction score with postnatal care	RR 14.70 [8.43, 20.97] (1/280)	Low	Yonemoto 2021	HIGH	PB	
**Post-discharge controls**	Home visits	No home visits	Infant jaundice	RR 1.04 [0.85, 1.26] (2/861)	Very low	Yonemoto 2021	HIGH	IE	
**Post-discharge controls**	Schedules involving more	Fewer home visits	Infant respiratory tract infection within 42 days	RR 0.99 [0.84, 1.17] (3/1217)	Low	Yonemoto 2021	HIGH	PEND	
**Post-discharge controls**	Home visits	No home visits	Infant respiratory tract infection within 42 days	RR 1.01 [0.89, 1.15] (2/865)	Very low	Yonemoto 2021	HIGH	IE	
**Post-discharge controls**	Schedules involving more home visits (more than 4	Fewer home visits- (more than 4 visits)	Infant respiratory tract infection within 42 days	RR 0.39 [0.12, 1.22] (1/352)	Very low	Yonemoto 2021	HIGH	IE	
**Post-discharge controls**	Home visits	No home visits	Infant diarrhoea up to 42 days postpartum	RR 0.85 [0.74, 0.98] (2/861)	Low	Yonemoto 2021	HIGH	PB	
**Post-discharge controls**	Schedules involving more	Fewer home visits	Exclusive breastfeeding (last assessment up to 6 weeks)	RR 1.17 [1.01, 1.36] ((3/960)	Low	Yonemoto 2021	HIGH	PB	
**Post-discharge controls**	Home visits	No home visits	Exclusive breastfeeding (last assessment up to 6 weeks)	RR 1.80 [1.00, 3.23] (1/60)	Very low	Yonemoto 2021	HIGH	IE	
**Post-discharge controls**	Schedules involving more home visits (4 or more)	Fewer home visits (less than 4)	Exclusive breastfeeding (last assessment up to 6 weeks)	RR 1.13 [1.05, 1.22] (1/352)	Low	Yonemoto 2021	HIGH	PB	
**Post-discharge controls**	Schedules involving more home visits (more than 4	Fewer home visits- (more than 4 visits)	Exclusive breastfeeding (last assessment up to 6 weeks)	RR 1.16 [0.89, 1.51] (1/548)	Low	Yonemoto 2021	HIGH	IE	

**Post-discharge controls**	Schedules involving more	Fewer home visits	Exclusive breastfeeding (last assessment up to 6 months)	RR 1.38 [1.10, 1.73] (4/1309)	Low	Yonemoto 2021	HIGH	PB	
**Post-discharge controls**	Home visits	No home visits	Exclusive breastfeeding (last assessment up to 6 months)	RR 1.50 [1.15, 1.94] (3/816)	Low	Yonemoto 2021	HIGH	PB	
**Post-discharge controls**	Schedules involving more home visits (more than 4	Fewer home visits- (more than 4 visits)	Exclusive breastfeeding (last assessment up to 6 months)	RR 1.06 [0.66, 1.69] (1/439)	Low	Yonemoto 2021	HIGH	IE	
**Post-discharge controls**	Flexible schedule	Routine visits	Neonatal mortality	RR 1.80 [0.16, 19.79] (1/2064)	Very low	Yonemoto 2021	HIGH	IE	
**Post-discharge controls**	Home visit	Telephone screen	Neonatal morbidity up to 28 days	RR 0.97 [0.85, 1.12] (1/696)	Moderate	Yonemoto 2021	HIGH	CEND	
**Post-discharge controls**	Breastfeeding promotion	Routine visits	Exclusive breastfeeding (last assessment up to 6 months)	RR 1.47 [0.81, 2.69] (1/656)	Low	Yonemoto 2021	HIGH	IE	
**Post-discharge controls**	Home	Facility visits	Severe maternal morbidity (emergency health care visits)	RR 1.04 [0.82, 1.33] (3/3242)	Low	Yonemoto 2021	HIGH	IE	
**Post-discharge controls**	Home	Facility visits	Severe maternal morbidity (hospital readmissions)	RR 1.32 [0.46, 3.82] (3/2690)	Very low	Yonemoto 2021	HIGH	IE	
**Post-discharge controls**	Home	Facility visits	Maternal satisfaction with postnatal care	RR 1.36 [1.14, 1.62] (3/2368)	Low	Yonemoto 2021	HIGH	PB	
**Post-discharge controls**	Home	Facility visits	Mean satisfaction score with postnatal care	MD -0.10 [-0.88, 0.68] (1/513)	Low	Yonemoto 2021	HIGH	IE	
**Post-discharge controls**	Home	Facility visits	Exclusive breastfeeding (last assessment up to 6 weeks)	RR 1.05 [0.93, 1.18] (1/513)	Moderate	Yonemoto 2021	HIGH	CEND	
**Post-discharge controls**	Home	Facility visits	Non prespecified - Infant emergency health care visits	RR 1.15 [0.95, 1.38] (3/3257)	Low	Yonemoto 2021	HIGH	IE	
**Post-discharge controls**	Home	Facility visits	Non prespecified - Infant hospital readmissions	RR 1.16 [0.57, 2.36] (3/2690)	Low	Yonemoto 2021	HIGH	IE	
**Time to resume physical activity**	-	-	-	-	-	-	-	no SR	
**Time to resume sexual activity**	-	-	-	-	-	-	-	no SR	
**Wound care**	-	-	-	-	-	-	-	no SR	

**Time to stich removal**	-	-	-	-	-	-	-	no SR	⊘
**Health system/ Other (to decide on title)**									
**Education/ information**	Information video	Control	Postoperative anxiety	MD -3.10 [-4.48, -1.73] (6/827)	Moderate	Abuzaid 2024	Critically Low	CEB	✔
**Education/ information**	Information video	Control	Postoperative satisfaction	RR 1.37 [0.76, 2.50] (6/790)	Low	Abuzaid 2024	Critically Low	IE	?
**Protocols**	-	-	-	-	-	-	-	no SR	⊘
**Companionship**	-	-	-	-	-	-	-	no SR	⊘
**Thromboprophylaxis**	Heparin (LMWH or UFH)	No treatment/placebo	Maternal death	Not estimable(1/300)	Very low	Middleton 2021	HIGH	IE	?
**Thromboprophylaxis**	Heparin (LMWH or UFH)	No treatment/placebo	Symptomatic thromboembolic events	RR 1.30 [0.39, 4.27] (1/840)	Very low	Middleton 2021	HIGH	IE	?
**Thromboprophylaxis**	Heparin (LMWH or UFH)	No treatment/placebo	Symptomatic pulmonary embolism	RR 1.10 [0.25, 4.87] (4/840)	Very low	Middleton 2021	HIGH	IE	?
**Thromboprophylaxis**	Heparin (LMWH or UFH)	No treatment/placebo	Symptomatic deep vein thrombosis	RR 1.30 [0.24, 6.94] (5/1140)	Very low	Middleton 2021	HIGH	IE	?
**Thromboprophylaxis**	Heparin (LMWH or UFH)	No treatment/placebo	Blood transfusion	RR 0.24 [0.03, 2.13] (3/266)	Very low	Middleton 2021	HIGH	IE	?
**Thromboprophylaxis**	Heparin (LMWH or UFH)	No treatment/placebo	Major bleeding	Not estimable (1/76)	Very low	Middleton 2021	HIGH	IE	?
**Thromboprophylaxis**	Heparin (LMWH or UFH)	No treatment/placebo	Major bruising	Not estimable (1/76)	Very low	Middleton 2021	HIGH	IE	?
**Thromboprophylaxis**	Heparin (LMWH or UFH)	No treatment/placebo	Bleeding complications	RR 5.03 [2.49, 10.18] (2/714)	Very low	Middleton 2021	HIGH	IE	?
**Thromboprophylaxis**	Heparin (LMWH or UFH)	No treatment/placebo	Bleeding/bruising reported at discharge	RR 6.17 [0.76, 49.96] (1/140)	Very low	Middleton 2021	HIGH	IE	?
**Thromboprophylaxis**	Heparin (LMWH or UFH)	No treatment/placebo	Blood loss < 500 mL	RR 1.50 [0.63, 3.59] (1/50)	Very low	Middleton 2021	HIGH	IE	?

**Thromboprophylaxis**	Heparin (LMWH or UFH)	No treatment/placebo	Blood loss 500-1000 mL	RR 0.81 [0.50, 1.31] (1/50)	Very low	Middleton 2021	HIGH	IE	
**Thromboprophylaxis**	Heparin (LMWH or UFH)	No treatment/placebo	Blood loss 1000-1500 mL	RR 0.50 [0.05, 5.17] (1/50)	Very low	Middleton 2021	HIGH	IE	
**Thromboprophylaxis**	Heparin (LMWH or UFH)	No treatment/placebo	Blood loss 1500-2000 mL	RR 2.00 [0.19, 20.67] (1/50)	Very low	Middleton 2021	HIGH	IE	
**Thromboprophylaxis**	Heparin (LMWH or UFH)	No treatment/placebo	Major wound disruption	Not estimable (1/126)	Very low	Middleton 2021	HIGH	IE	
**Thromboprophylaxis**	Heparin (LMWH or UFH)	No treatment/placebo	Surgical site infections (SSI)	RR 2.30 [0.34, 15.53] (2/216)	Very low	Middleton 2021	HIGH	IE	
**Thromboprophylaxis**	Heparin (LMWH or UFH)	No treatment/placebo	Adverse effects sufficient to stop treatment	Not estimable (1/140)	Very low	Middleton 2021	HIGH	IE	
**Thromboprophylaxis**	Heparin (LMWH or UFH)	No treatment/placebo	Adverse effects not sufficient to stop treatment	Not estimable (1/140)	Very low	Middleton 2021	HIGH	IE	
**Thromboprophylaxis**	Hydroxyethyl starch (HES)	Heparin (UFH)	Asymptomatic thromboembolic events	RR 0.76 [0.27, 2.11] (1/270)	Very low	Middleton 2021	HIGH	IE	
**Thromboprophylaxis**	Hydroxyethyl starch (HES)	Heparin (UFH)	Blood transfusion	RR 0.50 [0.05, 5.48] (1/207)	Very low	Middleton 2021	HIGH	IE	
**Thromboprophylaxis**	Hydroxyethyl starch (HES)	Heparin (UFH)	Bleeding episodes	RR 0.40 [0.08, 2.03] (1/207)	Very low	Middleton 2021	HIGH	IE	
**Thromboprophylaxis**	Hydroxyethyl starch (HES)	Heparin (UFH)	Serious wound complications	RR 0.67 [0.25, 1.82] (1/207)	Very low	Middleton 2021	HIGH	IE	
**Thromboprophylaxis**	Heparin (LMWH)	Heparin (UFH)	Symptomatic thromboembolic events	Not estimable (3/217)	Very low	Middleton 2021	HIGH	IE	
**Thromboprophylaxis**	Heparin (LMWH)	Heparin (UFH)	Symptomatic pulmonary embolism	Not estimable (3/217)	Very low	Middleton 2021	HIGH	IE	
**Thromboprophylaxis**	Heparin (LMWH)	Heparin (UFH)	Symptomatic deep vein thrombosis	RR 0.33 [0.01, 7.99] (3/217)	Very low	Middleton 2021	HIGH	IE	
**Thromboprophylaxis**	Heparin (LMWH)	Heparin (UFH)	Bleeding episodes "haemorrhagic event"	RR 0.33 [0.01, 7.99] (3/217)	Very low	Middleton 2021	HIGH	IE	

**Thromboprophylaxis**	Heparin (LMWH)	Heparin (UFH)	Major bleeding	Not estimable (1/17)	Very low	Middleton 2021	HIGH	IE	?
**Thromboprophylaxis**	Heparin (LMWH)	Heparin (UFH)	Post-surgical haemorrhage	Not estimable (1/100)	Very low	Middleton 2021	HIGH	IE	?
**Thromboprophylaxis**	Heparin (LMWH)	Heparin (UFH)	Adverse effects not sufficient to stop treatment	Not estimable (1/100)	Very low	Middleton 2021	HIGH	IE	?
**Thromboprophylaxis**	Heparin (LMWH)	Heparin (UFH)	Thrombocytopenia	Not estimable (1/100)	Very low	Middleton 2021	HIGH	IE	?

RR: Risk Ratio; **MD:** Mean difference; **CEB:** Clear evidence of benefit; **PB:** Possible benefit; **CEH:** Clear evidence of harm; **PH:** Possible evidence of harm; **CEND:** Clear evidence of no difference; **PEND:** Possible evidence of no difference; **IE:** Insufficient evidence; **SR:** Systematic review; **CS:** Caesarean section; **h:** hours; **RDS:** Respiratory Distress Syndrome; **LMWH:** Low Molecular Weight Heparin; **UFH:** Unfractionated Heparin; **HES:** Hydroxyethyl Starch
