# Supplementary material for: Evidence-based medical procedures to optimise caesarean outcomes: an overview of systematic reviews
Source: eClinicalMedicine. 2025 Apr 30;83:103212. doi: 10.1016/j.eclinm.2025.103212 (PMC12076788; doi:10.1016/j.eclinm.2025.103212)
Supplement: Abstract in Spanish [file mmc2.docx]

***The following translations in Spanish were submitted by the authors and we reproduce them as supplied. They have not been peer reviewed. Our editorial processes have only been applied to the original abstract in English, which should serve as reference for this manuscript***

**Abstract in Spanish**

**Resumen**

**Introducción:** El uso de cesárea está aumentando a niveles sin precedentes en todo el mundo. Cómo cualquier otra cirugía, tiene riesgos, y comprender la base de la evidencia para las intervenciones involucradas en un cesárea es esencial para optimizar resultados y generar recomendaciones. Realizamos una revisión de revisiones sistemáticas (RS) de ensayos controlados aleatorizados (ECA) para resumir la evidencia de los procedimientos médicos utilizados en las cesáreas.

**Métodos:** Se realizaron búsquedas en la Base de Datos Cochrane de Revisiones sistemáticas, PubMed, EMBASE, LILACS y CINAHL sin restricciones de fecha o idioma desde el inicio de la base de datos hasta el 31 de enero de 2024, con una búsqueda actualizada realizada el 24 de enero de 2025. Se incluyeron RS de ECA que examinaron la eficacia y la seguridad de los procedimientos médicos utilizados en la cesárea. Se utilizaron AMSTAR 2 y GRADE para evaluar la calidad metodológica de las RS y la certeza de la evidencia a nivel de resultados, respectivamente. Clasificamos cada par procedimiento-resultado en una de ocho categorías según las estimaciones del efecto y la certeza de la evidencia. La revisión se registró en PROSPERO (CRD42023208306).

**Resultados:** Identificamos 29 RS (15 Cochrane y 14 No Cochrane) publicadas entre 2002 y 2024 que involucraron 408 ECA únicos con más de 116.000 participantes. La mayoría de las revisiones incluyeron ensayos de países de ingresos bajos y medios (n=21, 72.4%), combinaron cesárea electiva y de emergencia (n=19, 65.5%), y fueron de alta calidad (n=18, 62%), mientras 24.3% (n=7) fueron de baja y 13.7% (n=4) fueron de muy baja calidad. Obtuvimos 512 comparaciones procedimiento-resultado de las RS (271 procedimiento vs procedimiento), 241 procedimiento versus ningún tratamiento/placebo). Hubo evidencia insuficiente o no concluyente para 350 comparaciones (68.4%), evidencia clara de beneficio para 97 (18.9%), posible beneficio para 48 (9.3%), clara o posible ausencia de diferencia de efecto para 9 (1.8%), evidencia clara de daño para 4 (0.8%) y posible daño para 4 (0.8%). No encontramos RS para 13 procedimientos preespecificados. El sondaje vesical permanente y su extracción inmediata, la limpieza vaginal con solución antiséptica, la profilaxis antibiótica, la ingesta oral temprana y las fajas abdominales se asocian con beneficios para alguno de los resultados. No hay RS sobre el cuidado de heridas post-cesárea, la extracción de puntos o el tiempo para reanudar la actividad sexual o física, entre otros.

**Interpretación:** Existen numerosas lagunas en la evidencia disponible sobre los procedimientos médicos utilizados en cesárea que requieren investigación adicional. Existe una necesidad urgente de recomendaciones internacionales para guiar a los proveedores de atención médica y a los responsables de la formulación de políticas para garantizar una atención más segura basada en evidencia para las mujeres que se somete a una cesárea

Financiamiento: Programa Especial de Investigación, Desarrollo y Capacitación en Investigación en Reproducción Humana (HRP) del PUND-UNFPA-UNICEF-OMS-Banco Muncial, un programa copatrocinado y ejecutado por la Organización Mundial de la Salud (OMS)
