# Supplementary material for: Evidence-based medical procedures to optimise caesarean outcomes: an overview of systematic reviews
Source: eClinicalMedicine. 2025 Apr 30;83:103212. doi: 10.1016/j.eclinm.2025.103212 (PMC12076788; doi:10.1016/j.eclinm.2025.103212)
Supplement: Abstract in Portuguese [file mmc3.docx]

***The following translations in Portuguese were submitted by the authors and we reproduce them as supplied. They have not been peer reviewed. Our editorial processes have only been applied to the original abstract in English, which should serve as reference for this manuscript***

**Resumo**

**Introdução**: O parto por cesariana (CS) está aumentando em todo o mundo e alcançou níveis sem precedentes. Como qualquer cirurgia, a CS tem riscos. Para poder otimizar os resultados desse tipo de parto e fazer recomendações, é importante entender as evidências sobre as intervenções usadas em uma CS. Fizemos uma Overview de revisões sistemáticas (RS) de ensaios clínicos randomizados (ECRs) para resumir as evidências sobre os procedimentos médicos usados durante uma CS.

**Metodologia:** Fizemos buscas nas bases de dados Cochrane Database of Systematic Reviews, PubMed, EMBASE, LILACS e CINAHL sem restrições de data ou idioma, do início da base até 31/01/2024 e atualizamos a busca em 24/01/2025. Incluímos ECRs que avaliaram a eficácia e segurança de procedimentos médicos usados em CS. Usamos o AMSTAR 2 e o GRADE para avaliar a qualidade metodológica das RSs e a certeza da evidência para cada desfecho, respectivamente. Classificamos cada par de procedimento-desfecho em uma dentre oito possíveis categorias conforme a estimativa de efeito e a certeza da evidência. Registramos o protocolo da Overview na plataforma PROSPERO (CRD 42023208306).

**Resultados:** Encontramos 29 RSs (15 Cochrane e 14 não-Cochrane) publicadas em 2002-2024, que incluíam 408 ECRs únicos com mais de 116.000 participantes. A maioria das revisões incluía ensaios clínicos conduzidos em países de baixa e média renda (n=21, 72,4%), envolvia tanto CSs eletivas como de emergência (n=19, 65,5%) e era de alta qualidade metodológica (n=18, 62%), porém 24.3% (n=7) eram de baixa qualidade e 13.7% (n=4) eram de qualidade metodológica muito baixa. As RSs apresentavam 512 comparações procedimentos-desfechos (271 procedimentos versus procedimentos e 241 procedimentos versus nenhum tratamento/placebo). Para 350 comparações (68,4%), a evidência era insuficiente ou inconclusiva, para 97 (18,9%) a evidência indicava um claro benefício do procedimento, para 48 (9,3%) a evidência indicava um possível benefício, para 9 (1,8%) a evidência indicava possível ou clara ausência de diferença de efeito, para 4 (0,8%) a evidência indicava um claro dano e para 4 (0,8%) outras comparações a evidência indicava um possível dano do procedimento. Para 13 procedimentos pré-especificados, não encontramos nenhuma RS. Procedimentos como o uso de cateteres vesicais de demora e sua remoção imediata, solução antisséptica para o preparo vaginal, antibióticos profiláticos, ingestão oral precoce e cintas abdominais estão associado a benefícios para vários desfechos. Não encontramos nenhuma RS sobre vários procedimentos, como cuidados da cicatriz de CS, retirada dos pontos ou tempo para retomar atividade física ou sexual, entre outros.

**Interpretação:** Há várias lacunas nas evidências disponíveis sobre procedimentos médicos usados em CSs que exigem pesquisas adicionais. Existe uma necessidade urgente de recomendações internacionais sobre CSs para orientar os profissionais de saúde e os formuladores de políticas e para garantir que as mulheres que dão à luz por esta via tenham um atendimento mais seguro e baseado em evidências.

**Financiamento:** UNDP-UNFPA-UNICEF-WHO-World Bank Special Programme of Research, Development and Research Training in Human Reproduction (HRP), um programa copatrocinado executado pela Organização Mundial da Saúde (OMS).

**Palavras chave:** Cesárea, procedimento médico, saúde pública, revisão sistemática, saúde materna
