# Supplementary material for: Evidence-based medical procedures to optimise caesarean outcomes: an overview of systematic reviews
Source: eClinicalMedicine. 2025 Apr 30;83:103212. doi: 10.1016/j.eclinm.2025.103212 (PMC12076788; doi:10.1016/j.eclinm.2025.103212)
Supplement: Abstract in French [file mmc4.docx]

***The following translations in French were submitted by the authors and we reproduce them as supplied. They have not been peer reviewed. Our editorial processes have only been applied to the original abstract in English, which should serve as reference for this manuscript***

**Abstact in French**

**Résumé**

**Contexte :** L'utilisation des césariennes atteint des niveaux sans précédent à l'échelle mondiale. Comme toute intervention chirurgicale, elle comporte des risques, et il est essentiel de comprendre les données des interventions impliquées dans une césarienne afin d’optimiser les résultats et d’orienter les recommandations. Nous avons réalisé une étude des revues systématiques d'essais contrôlés randomisés afin de résumer les résultats obtenus sur les procédures médicales utilisées lors des césariennes.

**Méthodes :** Des recherches ont été effectuées dans la Cochrane Database of Systematic Reviews, PubMed, EMBASE, LILACS et CINAHL, sans restriction de date ni de langue, depuis la création de la base de données jusqu'au 31 janvier 2024, avec une recherche mise à jour effectuée le 24 janvier 2025. Nous avons inclus les revues systématiques d'essais contrôlés randomisés qui ont examiné l'efficacité et la sécurité des procédures médicales utilisées dans les césariennes. Les outils AMSTAR 2 et GRADE ont été utilisés pour évaluer respectivement la qualité méthodologique des revues systématiques et le niveau de certitude des preuves pour les résultats obtenus. Nous avons classé chaque paire de procédure-résultat en huit catégories selon les estimations des effets et la certitude des preuves. Cette revue générale a été enregistrée sur PROSPERO (CRD 42023208306).

**Résultats :** Nous avons identifié 29 revues systématiques (15 Cochrane et 14 non-Cochrane) publiées entre 2002 et 2024, comprenant 408 essais contrôlés randomisés uniques incluant plus de 116 000 participants. La plupart des revues incluaient des essais menés dans des pays à revenu faible ou intermédiaire (n=21, 72.4%), combinaient les césariennes électives et en urgence (n=19, 65.5%), et étaient de haute qualité (n=18, 62%), alors que 24,3 % (n=7) étaient de faible qualité et 13,7 % (n=4) étaient de qualité critique. Les revues systématiques ont présenté 512 comparaisons de procédure-résultat (271 comparaisons entre procédures et 241 comparaisons entre procédure et absence de traitement/placebo).

Il y avait des preuves insuffisantes ou non concluantes pour 350 comparaisons (68.4 %), des preuves claires de bénéfice pour 97 (18.9%), un bénéfice possible pour 48 (9.3%), une absence d’effet clair ou possible pour 9 (1.8%), des preuves claires de préjudice pour 4 (0.8%) et un préjudice possible pour 4 (0.8%). Aucune revue systématique n’a été trouvée pour 13 procédures préalablement spécifiées.

L’utilisation d’un cathéter vésical et son retrait immédiat, la préparation vaginale avec une solution antiseptique, la prophylaxie antibiotique, l’alimentation orale précoce et l’utilisation de ceintures abdominales sont associées à des bénéfices pour certains critères de jugement. Il n'existe cependant aucune revue systématique sur les soins des plaies après une césarienne, le retrait des sutures ou le délai recommandé avant la reprise de l’activité sexuelle ou physique, entre autres.

**Interprétation :** De nombreuses lacunes existent quant à l’efficacité des procédures médicales utilisées lors des césariennes, ce qui justifie des recherches supplémentaires. Il est urgent d'établir des recommandations internationales pour guider les prestataires de soins de santé et les décideurs politiques afin d’assurer une prise en charge plus sûre et fondée sur des preuves pour les femmes qui accouchent par césarienne.
